# High-dimensional machine learning models for prediction of heart failure in more than 400 000 men and women from the UK Biobank

**DOI:** 10.1093/ehjdh/ztaf118

**Published:** 2025-10-06

**Authors:** Thomas F Kok, Navin Suthahar, Jesse H Krijthe, Rudolf A de Boer, Eric Boersma, Isabella Kardys

**Affiliations:** Department of Cardiology, Erasmus MC, Thorax Centre, Cardiovascular Institute, P.O. Box 2040, 3000 CA, Rotterdam, The Netherlands; Department of Cardiology, Erasmus MC, Thorax Centre, Cardiovascular Institute, P.O. Box 2040, 3000 CA, Rotterdam, The Netherlands; Pattern Recognition and Bioinformatics, TU Delft, Faculty of Electrical Engineering, Mathematics and Computer Science, van Mourik Broekmanweg 6, 2628XE, Delft, The Netherlands; Department of Cardiology, Erasmus MC, Thorax Centre, Cardiovascular Institute, P.O. Box 2040, 3000 CA, Rotterdam, The Netherlands; Department of Cardiology, Erasmus MC, Thorax Centre, Cardiovascular Institute, P.O. Box 2040, 3000 CA, Rotterdam, The Netherlands; Department of Cardiology, Erasmus MC, Thorax Centre, Cardiovascular Institute, P.O. Box 2040, 3000 CA, Rotterdam, The Netherlands

**Keywords:** Machine learning, Heart failure, Survival analysis, General population, Risk assessment, Big data

## Abstract

**Aims:**

We aimed to compare performances of conventional survival models with machine learning (ML) survival models for incident heart failure (HF) in men and women without prevalent HF, cardiomyopathy (CM) or ischaemic heart disease (IHD), and to identify potential high-risk precursors overlooked by conventional survival models.

**Methods and results:**

We predicted 10-year risk of incident HF in 266 306 women (2894 events) and 212 061 men (4213 events). We constructed multivariable Cox models, first using ∼ 400 baseline characteristics, and subsequently only those remaining after LASSO stability selection. We also used Random Survival Forest (RSF) and eXtreme Gradient Survival Boosting (XGBoost). Performances were assessed using internal cross validation and hold-out sets, with C-indices, calibration curves and net-benefit analyses. Model performances were comparable during internal validation: XGBoost (*C*-index ± SE) (men: 0.79 ± 0.0040, women: 0.83 ± 0.0023) showed similar performance to the multivariable Cox model (men: 0.80 ± 0.0031, women: 0.83 ± 0.0022) and Cox models after LASSO stability selection, while RSF showed numerically slightly lower performance (men: 0.78 ± 0.0025, women: 0.81 ± 0.0015). Findings were similar in the hold-out sets. Age, cystatin-C, lifetime treatments/medications, other heart disease, systolic blood pressure, and spirometry measures were identified as high-risk factors in both model types for both sexes. Additionally, sex-specific and model-specific risk factors were identified.

**Conclusion:**

Machine learning models and Cox proportional hazard models performed well and similarly for 10-year incident HF risk prediction in the general population. However, sex-specific and model-specific risk predictors were found. Spirometry measures, rarely included in existing models, were identified as important risk factors. Our results suggest that ML models for HF prediction in the general population reveal insights that would otherwise remain unnoticed.

## Graphical Abstract

**Graphical Abstract:**
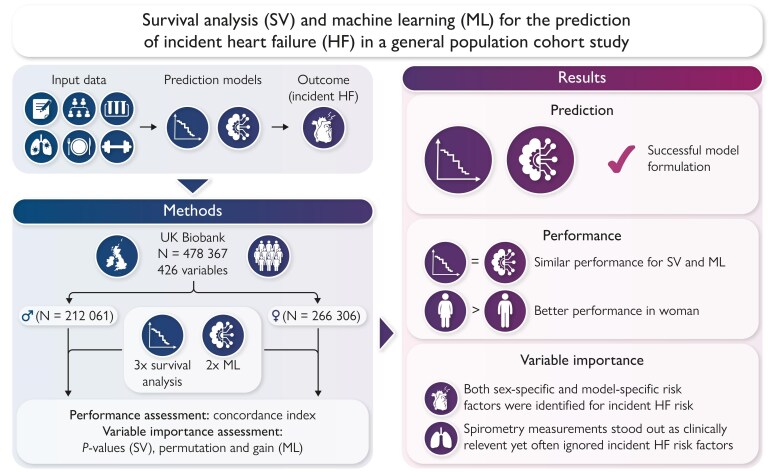

## Introduction

While several risk prediction models have been developed for heart failure (HF), their performance still leaves room for improvement, partly due to the multifactorial aetiology and heterogeneous nature of HF. A systematic review^1^ observed 58 HF risk prediction models, published between 2012 and 2018. The authors concluded that models were limited by a lack of methodological information, high risk of bias and absence of either internal- or external validation, and postulated the potential of machine learning (ML) tools for HF prediction.

Risk models are typically developed as multivariable regression models that use small numbers of well-established predictor variables, and usually assume simple linear relationships between (log-scaled) predictor variables and outcomes.^2^ The inclusion of a larger number of variables provides opportunities to identify potential lesser-known HF risk factors and herewith improve model performance. Although it has been suggested that characteristics of a prediction model are less important than the type and number of used predictor variables in predicting HF outcome,^3^ previous studies exploring benefits of ML techniques for HF prediction carried limitations, such as variable preselection and use of relatively small datasets.^4^

Moreover many existing models pay insufficient attention to sex differences. Sex is often included as determinant of outcome, yet interactions between sex and other characteristics are frequently neglected. Although age-related risk of HF and in-hospital HF mortality have shown similarities between sexes, sex differences can become apparent when examining how traditional risk factors influence risk in either sex.^5,6^ Women tend to be older at time of first in-hospital diagnosis and are more inclined to develop HF with preserved ejection fraction (HFpEF), compared with men who are more prone to develop HF with reduced ejection fraction (HFrEF).^7^ Diabetes and hypertension, both strong traditional risk factors, show greater risk of HF development in women than men, as shown in the Framingham Heart Study.^8^ Obesity has been described as a significant risk factor for HFpEF development, especially in postmenopausal women.^9^ Because of these underlying differences, prediction models, which take sex into account properly, are useful.

In this study, we developed sex-specific supervised ML prediction models for incident HF in the general UK Biobank population using a hypothesis-free approach. We compared prediction performance to traditional Cox proportional hazard (PH) models and investigated differences between sex-specific models. Moreover, we aimed to identify potential high-risk precursors otherwise ignored by conventional survival models.

## Methods

### Study design

The UK Biobank dataset is a large-scale population cohort featuring 502 407 participants living in the UK. The study design has been previously described.^10^ Participants aged 37 years and over were recruited between 2006 and 2010 and sent to 1 of 22 assessment centres in England, Scotland and Wales. Written consent was provided by participants prior to enrolment. Participants underwent physical examinations, provided blood and urine samples, completed automated questionnaires and answered lifestyle, medical history, and nutritional questions during an interview with a professional nurse. The full UK Biobank protocol can be found online.^11^ Approval was obtained from the North West Multi-centre Research Ethics Committee and the Community Health Index Advisory Group. Furthermore, the Quality and Information Security Management systems at UK Biobank have been approved by the British Standards Institution and are certified to ISO 9001:2015 and ISO 27001:2013 for the collection, processing, storing and analysis of genetic and environmental information respectively. Follow-up hospitalization data was obtained through mapping of hospital episode records across England, Scotland and Wales, combined with linking to national death and cancer registries and hospital admission data with linkage to primary care.^12^

**Figure 1:**
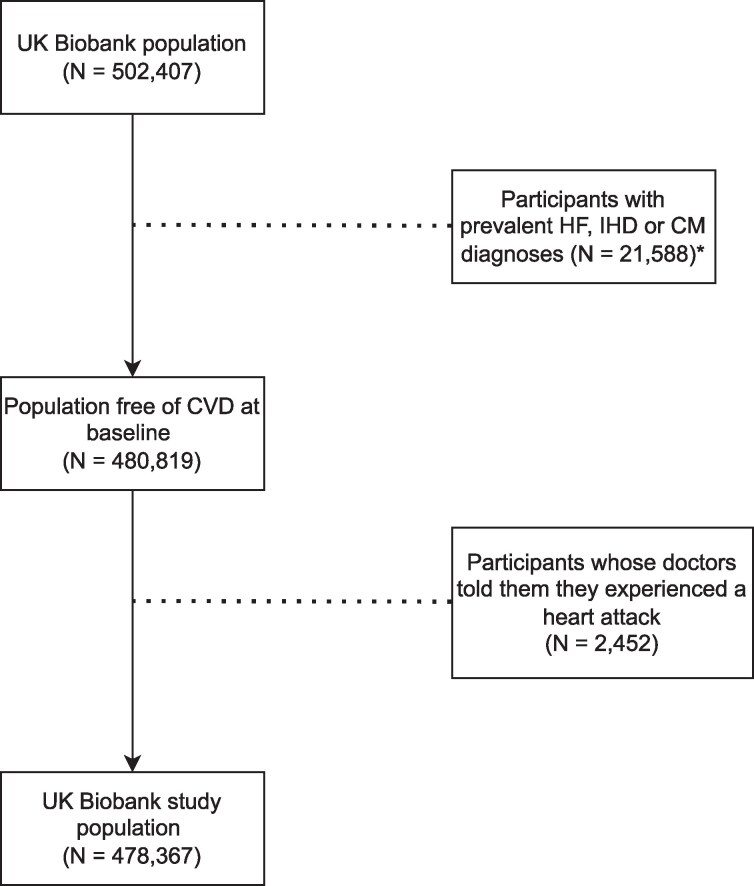
Flowchart of study population selection. *Prevalent diagnoses for heart failure, ischaemic heart disease and cardiomyopathy were stored as either ICD-10 or ICD-9 codes.

For this study, participants were excluded who had prevalent inpatient hospital records of HF, cardiomyopathy (CM) or ischaemic heart disease (IHD) reflected by codes ICD-10 or ICD-9 (*n* = 21 588); or who were told by their doctor they had experienced a heart attack (*n* = 2452). After exclusion, 2 datasets containing 266 306 women and 212 061 men remained. A flow chart of participant selection is shown in *Figure 1*.

### Study endpoint

The primary outcome was incident ICD10 in-hospital diagnosis of I50 (congestive HF, left ventricular failure, and unspecified HF). We censored for all-cause mortality. Survival time was the time in days between the date of UK Biobank assessment visit and an in-hospital diagnosis of incident HF event or, for patients without incident HF, time until the reported day of death or censoring (reaching the final observation date). Censoring occurred at 10 years of follow-up.

### Statistical analysis

An overview of the data flow during the analysis process, can be found in the Supplementary material online, *Figure S1*. Below, each analytical step is described in further detail.

### Data preprocessing

We included 426 variables in men and 437 in women. Baseline variables are described in the Supplementary material online, *Tables S1*–*S12*, and include early life factors, family history, medical history, lifestyle and environment, physical measures, psychosocial factors, socio-demographics, verbal interview, measurements from biological samples, and female-specific factors. Additionally, we included a set of prevalent history variables for all available ICD10 data (see Supplementary material online, *Table S13*).

Details on data preprocessing, aggregation, and preparation can be found in the Supplementary material.

### Data splitting and imputation

Each sex specific dataset was split in a train (80%) set, in which models were internally validated through stratified five-fold cross-validation, and a hold-out dataset (20%) used for external validation. Stratified sampling was used to create train- and hold-out sets with comparable proportions of the primary endpoint. Supplementary material online, *Table S14* shows numbers of participants and incident HF cases for both train- and hold-out sets.

Additionally, we formulated subsets for variable importance assessment, to account for the low prevalence of the primary endpoint in the study population, i.e. class imbalance. Subsets included all incident HF cases and four times the amount of randomly selected participants without incident HF. This resulted in a female subset (*n* = 14 470) and a male subset (*n* = 21 065), which were also split in 80% train- and 20% hold-out sets. Multiple imputation by chained equations with random forests, using predictive mean matching, featured in the *MiceRanger (1.5.0)* package, was used to impute missing data. Missing data was imputed in five iterations ensuring proper convergence. To prevent data leakage between train- and hold-out sets, imputation was performed after stratified splitting had occurred. Furthermore, to prevent data leakage during internal model validation, train data was split in five stratified folds of which, iteratively, four-folds were combined and imputed (train set) while the fifth was imputed separately (test set). Details about missing data, stratified by sex, can be found in Supplementary material online, *Table S15*.

### Traditional survival models

We formulated three types of Cox PH models for incident HF. First we ran a multivariable Cox PH model, featuring all baseline variables. The two other Cox PH models only contained baseline variables that remained after least absolute shrinkage and selection operator (LASSO) stability selection and randomized LASSO stability selection. LASSO stability selection combines stability selection^13^ with LASSO regularization.^14^ Stability selection identifies important predictor variables by repeatedly running variable selection over subsampled datasets. Variables present in at least 80% of created subsampled selection sets, were included in the Cox PH models. LASSO stability selection uses regularization parameter *λ* while random LASSO stability selection allows *λ* to vary between *λ* and *λ*/*α* where *α* is a weakness term, borrowed from the terminology of weak greedy algorithms.^15^ We used α=1 for LASSO stability selection and α=0.8 for randomized LASSO stability selection.

### Machine learning models

Random Survival Forest (RSF)^16^ is an ensemble tree-based ML method for the analysis of right-censored survival data and built as an extension on random forest analysis which only performs regression and classification.^17^ RSF explores non-linear relationships between predictor variables and outcome in high-dimensional data using many independent decision trees. Extreme Gradient Boosting (XGBoost)^18^ is a flexible ML method which combines a gradient boosting framework with a decision tree ensemble method similar to RSF. Unlike RSF, iteratively, one decision tree is added at a time, to correct the errors of the ensemble of decision trees up to that point, until optimal outcome prediction is reached. To facilitate survival analysis, the XGBoost learning objective for Cox regression for right censored survival data was used.

Both ML techniques went through hyperparameter optimization. Definitions and range of explored parameters are shown in Supplementary material online, *Table S16*. For RSF, hyperparameter optimization was done using a grid search in which the optimal out-of-sample error was found using the *C*-index as evaluation metric. Permutation importance was used to assess variable importance in the RSF models.

For XGBoost, optimal hyperparameters were identified using five-fold cross validation and evaluated using *C*-indices. Iterations were halted when the *C*-index of the validation set did not significantly increase for 30 iterations. Variable importance was assessed using the gain parameter.

Variable importance assessment was only done for the sex-specific subsets, using all available train data. For both ML methods, standard errors of *C*-indices in the hold-out sets were not readily available. Standard errors were calculated using an approximation based on the Mann–Whitney statistic.^19^ Furthermore, pairwise correlation between high-risk variables, identified in any model, was assessed to account for collinearity.

### Model performance assessment

We evaluated discriminative ability by means of Harrell’s concordance index (*C*-index).^20^ Moreover, for further performance assessment in the subsets, net-benefit- and calibration curves were constructed. Apart from performance assessment of our full models as specified above, we also compared performance of ML models that incorporated all available covariates, to ML models incorporating only variables that remained after LASSO stability selection. Additionally, we applied the established Pooled Cohort Equations to Prevent HF (PCP-HF) score^21^ to our study population, and compared its performance to that of our models.

### *R* and package information

All models were fitted in *R (4.2.3)*. Cox models were fitted using the *coxph* function in the *survival (3.5-5)* package. LASSO stability selection was done using the *randLassoStabSel* function found in the *monaLisa (1.7.1)* Bioconductor package. Random Survival Forest was performed using the *RandomForestSRC (3.2.2)* package. XGBoost was performed using the *xgboost (1.7.5.1)* package. Hyperparameter optimization was done for RSF using the *tune()* function, as described in the *RandomForestSRC (3.2.2)* package, and for XGBoost using the *caret (6.0-94)* package combined with the *xgbTree* method. Calibration plots were generated using the *CalPlot()* function from the *pec (2023.04.12)* package and net-benefit analysis plots were generated using the *dca()* function from the *dcurves (0.5.0)* package.

## Results

### Baseline characteristics

A total of 266 306 women and 212 061 men were included. Baseline characteristics are shown in *Table 1* and Supplementary material online, *Table S17* for the total participant population and subsets, respectively. In the total population, median (25th–75th percentile) age was similar between men [58.0 (50.0–63.0) years] and women [57.0 (50.0–63.0)]. Men were more likely to be current smokers (13.0% vs. 8.9%), had larger waist circumference [96.0 (89.0–103.0) vs. 83.0 (75.0–92.0) cm] and had higher forced expiratory volume in 1 second (FEV1) measures [3.34 (2.84–3.83) vs. 2.41 (2.05–2.76) L] than women. Men were also more likely to have prevalent diabetes (2.1% vs. 1.3%), other heart disease (defined as among others pericardial disease, valve disorders, and arrhythmias) (2.0% vs. 1.2%) and more often used blood pressure (BP) medication (21% vs. 17%) than women.

**Table 1:** Baseline characteristics for the total UK Biobank population (*n* = 478 367)

Characteristic	Men, *n* = 212 061	Women, *n* = 266 306
Age (years)	58 (50, 63)	57 (50, 63)
Ethnic background		
Any other white background	5945 (2.8%)	9882 (3.7%)
British	187 520 (88.4%)	234 443 (88.0%)
Indian	2704 (1.3%)	2835 (1.1%)
Irish	5868 (2.8%)	6701 (2.5%)
Other	10 024 (4.7%)	12 445 (4.7%)
Waist circumference (cm)	96 (89, 103)	83 (75, 92)
BMI (kg/m^2^)	27.2 (24.9, 29.9)	26.1 (23.4, 29.6)
Best FEV1 measure (L)	3.34 (2.84, 3.83)	2.41 (2.05, 2.76)
Systolic blood pressure (mmHg)	141 (130, 154)	135 (122, 150)
Diastolic blood pressure (mmHg)	84 (77, 91)	80 (73, 88)
Smoking status		
Current	26 595 (12.5%)	23 689 (8.9%)
Never	106 405 (50.2%)	159 480 (59.9%)
Previous	79 061 (37.3%)	83 137 (31.2%)
Albumin (g/L)	45.55 (43.85, 47.25)	44.94 (43.25, 46.67)
Cystatin-C (mg/L)	0.91 (0.84, 1.00)	0.85 (0.78, 0.95)
CRP (mg/L)	1.28 (0.66, 2.53)	1.37 (0.65, 2.97)
Creatinine (mmol/L)	79.8 (72.4, 88.0)	63.0 (57.0, 69.9)
Cholesterol (mmol/L)	5.53 (4.81, 6.28)	5.84 (5.11, 6.62)
HDL cholesterol (mmol/L)	1.25 (1.07, 1.47)	1.56 (1.32, 1.83)
Haemoglobin concentration (g/dL)	15.02 (14.40, 15.68)	13.50 (12.90, 14.10)
HbA1c (mmol/mol)	35.2 (32.7, 37.9)	35.1 (32.7, 37.7)
RDW (%)	13.30 (12.90, 13.80)	13.36 (12.90, 13.90)
Presence of other heart disease	4325 (2.0%)	3160 (1.2%)
Presence of diabetes	4437 (2.1%)	3351 (1.3%)
Blood pressure medication	44 482 (21%)	44 034 (17%)

BMI, body mass index; FEV1, forced expiratory volume in 1 s; CRP, C-reactive protein; HDL, high-density lipoprotein; HbA1c, glycated haemoglobin; RDW, red blood cell distribution width.

### Incident heart failure

**Figure 2:**
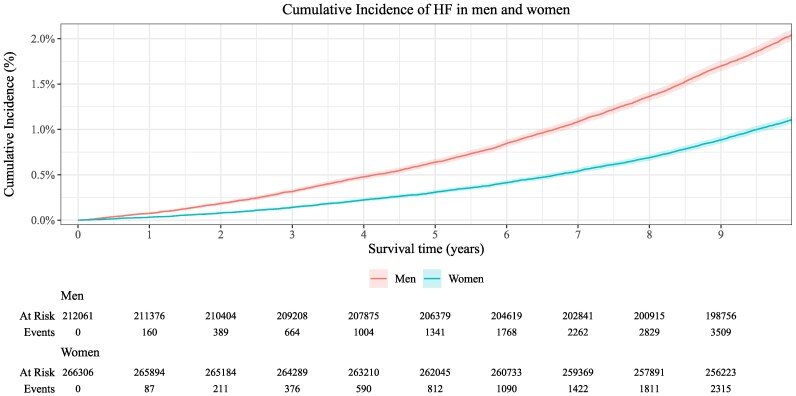
Cumulative incidence of heart failure with 95% confidence intervals in the UK Biobank population, stratified by sex. Survival time is given in years.

Follow-up was censored at 10 years, and during median follow-up times of 10.0 years (25th–75th percentile: 10.0, 10.0) in women and 10.0 years (25th–75th percentile: 10.0, 10.0) in men, there were 2894 (1.09%) incident HF events in women and 4213 (1.99%) in men. Cumulative incidence of HF is shown in *Figure 2*.

Baseline characteristics, stratified by incident HF status, are shown in Supplementary material online, *Table S18* for the full cohort and in Supplementary material online, *Tables S19* and *S20* for men and women. Overall, individuals who developed HF, were generally older at baseline [median (interquartile range) age, 63 (59–67) years vs. 57 (50–63) years], more often male (59% vs. 41%) and were more likely to have a medical history of other heart disease (8.2% vs. 1.4%), diabetes (7.1% vs. 1.5%), and BP medication use (43% vs. 18%).

### Model performance

Overall, traditional survival models and ML models showed similarities in performance, both for internal five-fold cross validation and for validation in the hold-out sets (*Table 2*). During training, using the full data, *C*-statistics ranged between 0.78 and 0.80 in men and 0.81 and 0.83 in women. In the hold-out sets, *C*-statistics varied between 0.78 and 0.80 in men and 0.80 and 0.82 in women. Model performance was numerically higher in women than in men for all five models. *C*-indices in the subsets were generally somewhat lower than in the full datasets, and the aforementioned sex-difference persisted. Overall, model performances numerically outperformed the PCP-HF score (see Supplementary material online, *Table S21*). Net benefit analyses (see Supplementary material online, *Figures S2* and *S3*) showed that net benefit of the Cox models was generally lower than that of both ML methods in both men and women. Net benefit was highest for RSF at low threshold probabilities, and highest for XGBoost at high threshold probabilities. Net benefit differences were less pronounced in women than men. The calibration plots (see Supplementary material online, *Figures S4* and *S5*) showed that, at high predicted event probabilities, the ML models tended to underestimate the risk of HF, while the Cox models tended to overestimate. Overall, Cox models showed better calibration compared with XGBoost and RSF.

**Table 2:** Model performance overview

Data	Sex	Cox PH model		Randomized LASSO stability selection		LASSO stability selection		RSF		XGBoost	
		Train	H.o-set	Train	H.o-set	Train	H.o-set	Train	H.o-set	Train	H.o-set
Full set	Men	0.80 ± 0.0031	0.80 ± 0.0077	0.78 ± 0.0024	0.79 ± 0.0078	0.78 ± 0.0022	0.79 ± 0.0078	0.78 ± 0.0025	0.78 ± 0.0079	0.79 ± 0.0040	0.80 ± 0.0077
	Women	0.83 ± 0.0022	0.81 ± 0.0092	0.82 ± 0.0022	0.81 ± 0.0090	0.82 ± 0.0025	0.82 ± 0.0091	0.81 ± 0.0015	0.80 ± 0.0093	0.83 ± 0.0023	0.81 ± 0.0091
Subset	Men	0.75 ± 0.0022	0.77 ± 0.0077	0.76 ± 0.0025	0.76 ± 0.0079	0.77 ± 0.0018	0.76 ± 0.0079	0.76 ± 0.0027	0.75 ± 0.0093	0.77 ± 0.0033	0.78 ± 0.0089
	Women	0.77 ± 0.0053	0.77 ± 0.0088	0.78 ± 0.0066	0.79 ± 0.0084	0.78 ± 0.0065	0.79 ± 0.0085	0.78 ± 0.0050	0.79 ± 0.010	0.80 ± 0.0062	0.80 ± 0.010

*C*-statistics for sex-specific traditional Cox models and ML methods for incident HF prediction in the full data and subsets. Train *C*-statistics were obtained through five-fold internal cross-validation while holdout *C*-statistics were obtained through external validation.

*C*-statistics are defined as *C*-index ± SE.

Cox PH, Cox proportional hazard; LASSO, least absolute shrinkage and selection operator; RSF, Random Survival Forest; XGBoost, eXtreme Gradient Boosting; H.o-set, hold-out set; SE, standard error.

### Variable importance and sex differences

We identified 21 high-risk variables in both sexes, 13 in only men and 20 in only women.

**Figure 3:**
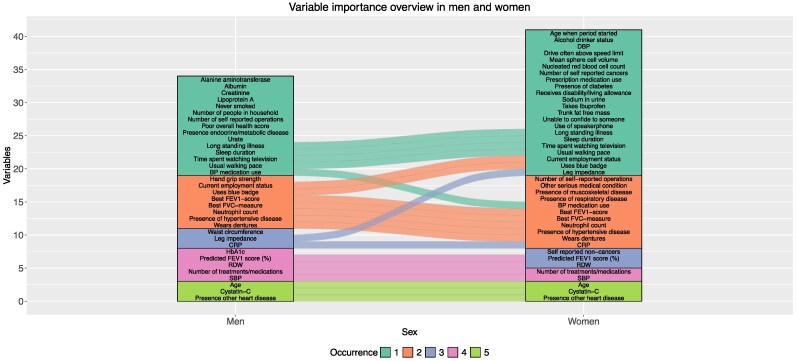
The alluvial plot shows the number of occurrences of high-risk variables in each of the models, for the 15 most important predictor variables. There are 34 distinct predictors identified in men and 41 in women with an overlap of 21 variables in both sexes. The flows show how occurrence varies between men and women. Absence of flow means that this variable was only present in one sex.

**Figure 4:**
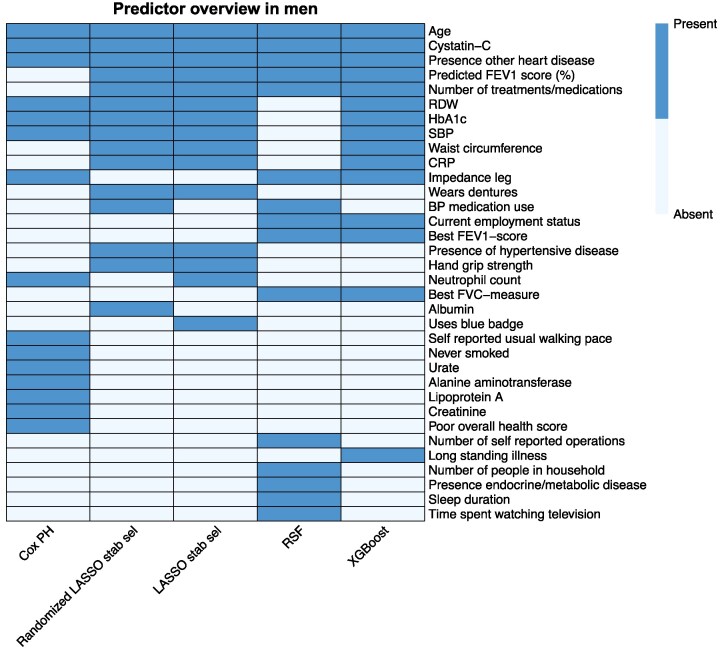
The heatmap shows, for each tested prediction model, which predictor variables were part of the 15 most important predictor variables in men.

**Figure 5:**
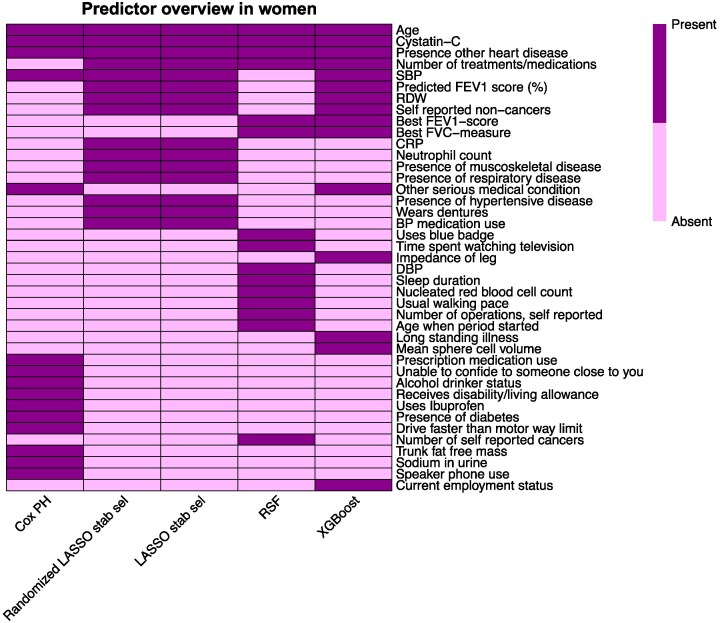
The heatmap shows, for each tested prediction model, which predictor variables were part of the 15 most important predictor variables in women.

Top 15 variable importance is shown for men and women in *Tables 3* and *4*, respectively. The scores used to construct both tables, can be found in the Supplementary material online, *Tables S22* and *S23* and, additionally, the importance metrics of the ML models are visualized in the Supplementary material online, *Figures S6* and *S7*. *Figure 3* provides an overview of model occurrences of high-risk variables, stratified by sex. *Figures 4* and *5* display heat maps of presence and absence of high-risk variables in each model. Correlation information between high-risk predictor variables, can be found in the Supplementary material online, *Figures S8* and *S9*. Positive correlation was strongest between several spirometry metrics and between cystatin-C and creatinine. Negative correlation was strongest between age and spirometry metrics. Adiposity metrics showed both strong positive-and negative correlations.

**Table 3:** The 15 most important predictor variables in men

Cox PH model	Randomized LASSO stability selection	LASSO stability selection	RSF model	XGBoost survival model
Presence other heart disease	Waist circumference	Waist circumference	Age	Age
Age	Number of treatments/medications	Number of treatments/medications	Number of treatments/medications	Number of treatments/medications
SBP	Age	**Predicted FEV1 score (%)**	Number of people in household	Cystatin-C
**Never smoked**	RDW	Age	Presence of other heart disease	Best FEV1 score
RDW	Cystatin-C	RDW	Cystatin-C	Predicted FEV1-score (%)
Usual walking pace	HbA1c	Cystatin-C	Best FEV1-score	Presence of other heart disease
**Alanine aminotransferase**	Presence other heart disease	HbA1c	Best FVC measure	Current employment status
HbA1c	BP medication use	Uses blue badge^a^	Use blue badge^a^	Best FVC measure
Creatinine	Presence of hypertensive disease	Presence of other heart disease	Sleep duration	Long standing illness, disability or infirmity
Urate	Wears dentures	Presence of hypertensive disease	Leg fat impedance	SBP
Poor overall health score	**Predicted FEV1 score (%)**	Wears dentures	Predicted FEV1 score (%)	CRP
Lipoprotein A	SBP	SBP	Current employment status	Waist circumference
Cystatin-C	Hand grip strength	Hand grip strength	Presence of other endocrine/metabolic disease	HbA1c
Neutrophil count	CRP	CRP	Number of self reported operations	RDW
Leg fat impedance	**Albumin**	Neutrophil count	Time spent watching television	Leg fat impedance

Most relevant incident HF predictors for men in the subset data. For Cox PH and the (randomized) LASSO stability selection, the first 15 variables are shown and variables are ordered from lowest to highest *P*-value. Reduced risk (HR <1) factors in the Cox PH models are written using a bold font. Features in ML models are ordered by permutation importance in RSF and gain in XGBoost.

Cox PH, Cox proportional hazard; LASSO, least absolute shrinkage and selection operator; RSF, Random Survival Forest; XGBoost, eXtreme Gradient Boosting; SBP, systolic blood pressure; Age, age in years when attending assessment centre; HbA1c, glycated haemoglobin; RDW, red blood cell distribution width; CRP, C-reactive protein; FEV1, forced expiratory volume in 1 s; FVC, forced vital capacity; BP, blood pressure.

^a^Blue badge is a service allowing people with severe mobility problems to park close to their destinations.

**Table 4:** The 15 most important predictor variables in women

Cox PH model	Randomized LASSO stability selection	LASSO stability selection	RSF model	XGBoost survival model
Presence other heart disease	Age	Age	Presence other heart disease	Cystatin-C
Age	Presence other heart disease	Presence of other heart disease	Age	Age
SBP	Number of treatments/medications.	**Predicted FEV1 score (%)**	Sleep duration	Best FEV1-score
Prescription medication use	Number of self-reported non-cancer illnesses.	Cystatin-C	Number of treatments/medications	Number of treatments/medications
Other serious medical condition/disability diagnosed by doctor	Cystatin-C	Number of treatments/medications	Number of self-reported operations	Presence other heart disease
Presence of diabetes	**Predicted FEV1 score (%)**	Number of self-reported non-cancer illnesses.	Number of self reported cancers	Predicted FEV1 score (%)
Trunk fat free mass	Presence of respiratory disease	Presence of respiratory disease	Usual walking pace	Long standing illness, disability or infirmity
Cystatin-C	Wears dentures	SBP	Cystatin-C	SBP
Takes Ibuprofen	SBP	Wears dentures	Age when period started	Leg fat impedance
Unable to confide to someone close	CRP	RDW	Uses blue badge^a^	Number of self reported non-cancer illnesses
Sodium in urine	RDW	Neutrophil count	Time spent watching television	Other serious medical condition/disability diagnosed by doctor
Receives disability/living allowance	Presence of muscoskeletal disease	Presence of hypertensive disease	Nucleated red blood cell count	Best FVC measure
Alcohol drinker status	Neutrophil count	BP medication use	Best FEV1-score	RDW
Uses speakerphone	Presence of hypertensive disease	Presence of muscoskeletal disease	DBP	Mean sphered cell volume
Often drive faster than motorway limit	BP medication use	CRP	Best FVC measure	Current employment status

Most relevant incident HF predictors for women in the subset data. For Cox PH and the (randomized) LASSO stability selection, the first 15 variables are shown and variables are ordered from lowest to highest *P*-value. Reduced risk (HR <1) factors in the Cox PH models are written using a bold font. Features in ML models are ordered by permutation importance in RSF and gain in XGBoost.

Cox PH, Cox proportional hazard; LASSO, least absolute shrinkage and selection operator; RSF, Random Survival Forest; XGBoost, eXtreme Gradient Boosting; SBP, systolic blood pressure; DBP, diastolic blood pressure; Age, age in years when attending assessment centre; HbA1c, glycated haemoglobin; RDW, red blood cell distribution width; CRP, C-reactive protein; FEV1, forced expiratory volume in 1 s; FVC, forced vital capacity; BP, blood pressure.

^a^Blue badge is a service allowing people with severe mobility problems to park close to their destinations.

In both sexes, age, cystatin-C, and the presence of other heart disease were the only three predictor variables present in all five models. The importance metrics plots show that, while these three predictor variables were indeed strongly associated with incident HF in both sexes, they did not cancel out other predictor variables (see Supplementary material online, *Figures S6* and *S7*). Furthermore, the effect of age was weaker in women than men. The number of self-reported lifetime treatments/medications was important in four models in both sexes, while diastolic BP (DBP) was present in the fifth model in women instead.

Sex differences were also present between the models; glycated haemoglobin (HbA1c) was important in four models in men but none in women. The number of self-reported non-cancers was important in three models in women but none in men. Red blood cell distribution width (RDW) and predicted FEV1 score (%) were present in four models in men and three in women. Leg impedance was present in three models in men and one in women. Detailed model-specific importance characteristics are described below.

In men, the ordinary multivariable Cox PH model was the only model to identify smoking, urate, lipoprotein A, creatinine, a poor overall health score, alanine aminotransferase, and walk metrics as a major risk for incident HF development, while prescription medication use, alcohol drinker status, inability to confide in someone close, use of ibuprofen, the presence of diabetes, trend to drive faster than motor way speed limit, trunk fat free mass, sodium in urine, speaker phone use, and receiver of disability/living allowance was only identified by this model in women.

LASSO stability selection reduced the number of variables from 426 down to 15 (randomized LASSO) and 18 (LASSO) in men, and from 437 down to 18 (randomized LASSO) and 18 (LASSO) in women. Further details are provided in Supplementary material online, *Table S24*.

For both stability selection models, waist circumference was the most important risk factor in men, followed by the number of treatments/medications. In women, age and presence of other heart disease were most important in both models; the third to seventh most important variables were predicted FEV1 score (%), cystatin-C, number of treatments/medications, number of self-reported non-cancer illnesses, and presence of respiratory disease. Rankings showed similarities in men, although HbA1c, RDW, and presence of hypertensive disease, were more important.

The LASSO stability selection models were the only models to identify hand grip strength, dentures use, blue badge use, presence of hypertensive disease and serum albumin as high-risk variables in men. At the same time, they were the only ones to identify C-reactive protein (CRP), neutrophil count, presence of muscoskeletal-, hypertensive- or respiratory disease, denture use, and BP medication use, as important in women. Beta estimates of the ordinary Cox model and both LASSO stabilized Cox models are shown in Supplementary material online, *Tables S25*–*S27*.

The ML models were the only models to select current (i.e. baseline) employment status, best FEV1-scores, and best forced vital capacity (FVC) measures as high-risk factors in both sexes. Random Survival Forest was the only model to identify sleep duration and time spent watching television as high-risk factors in both sexes. In men, RSF was the only model to identify the number of self-reported operations, number of people in household, and presence of endocrine/metabolic disease as high-risk factors. At the same time, it was the only model to consider DBP, nucleated red blood cell count, walking metrics, age period started, number of self-reported operations and self-reported cancers as important in women. XGBoost was the only model to identify long standing illness as a risk factor in both sexes and mean sphere cell volume in women. When only the predictor variables, that remained after LASSO stability selection, were used to train the ML models, their performances were comparable to the ML models trained on all available predictor variables (see Supplementary material online, *Table S28*).

## Discussion

We used over 400 predictor variables, measured in over 450 000 UK individuals, to compare traditional and contemporary Cox PH models, and two ML survival models, for incident HF prediction in men and women without a history of HF, CM and IHD. Machine learning survival models performed similar to Cox PH models although both sex-specific and model-specific risk predictors could be identified. In general, age, cystatin-C, and presence of other heart disease, were the most consistently important predictor variables. In men, waist circumference and HbA1c were more important, while medical disease history was more important in women. The ML models showed higher priority for spirometry metrics and employment status, while LASSO stability selection models prioritized denture use, neutrophil count, and medical disease history.

Performances of Cox PH models and ML models were similar in terms of the *C*-index, with XGBoost showing slight numerical performance superiority to RSF. Furthermore, overall, XGBoost showed the best net-benefit curves. For RSF, net benefit decreased drastically as threshold probability increased. Unlike the Cox PH models and XGBoost, which showed skewed predicted risk distributions, the predicted risk for RSF showed a sigmoid distribution. This probably contributed to the shape of the net benefit curve. Also, RSF and XGBoost tended to underestimate the risk of incident HF, while the Cox models were prone to overestimation; overall, the Cox models showed the most stable calibration.

Despite performance similarities, the ordinary Cox PH model showed the largest deviations in predictor importance compared with the other models. Different methodologies for variable importance assessment, the ability of ML to model non-linear relationships between predictors and endpoint, and the large number of available covariates may have contributed to these findings.

A retrospective multicohort analysis, which compared conventional survival models and ML models for 10-year incident HF risk prediction, showed superior performance of ML models, particularly when ML models were stratified by race.^22^ Also, previous research in the prediction of breast cancer survival demonstrated similar performances of Cox models and multiple ML models with XGBoost outperforming all models.^23^ Research on prediction of survival in pancreatic cancer found that Cox models performed slightly better than RSF.^24^ A meta-analysis^25^ investigated performance and reliability of 202 statistical- and 78 ML models for predicting all-cause mortality and all-cause readmission in HF patients, and found no apparent superiority of either model types. While our findings agree with these observations, this meta-analysis did not consider incident HF as an endpoint. Overall, our findings can be used to (i) extend the overall notion that ML and traditional Cox models have similar performances for incident HF risk in a general population and (ii) ML models imply additional determinants of incident HF, not found by traditional survival analysis.

Spirometry metrics (FEV1, FVC) were considered important in both the LASSO stability selection Cox PH models, and ML models, in both sexes. Spirometry metrics are used to check lung performance and support diagnosis and severity classification of chronic lung diseases like asthma and chronic obstructive pulmonary disease (COPD). Chronic obstructive pulmonary disease and HF often coexist in clinical practice, yet recognizing HF in the presence of COPD is complicated by symptom similarities like old age and tobacco smoking.^26^ A previous study concluded that FEV1 is just as important in the prediction of IHD mortality as cholesterol.^27^ Our results relate these findings to HF, and imply that spirometry measures, even in the absence of asthma or COPD, hold prognostic value for incident HF development. Our correlation analyses showed strong negative correlations between age and various spirometry metrics; still, both age and spirometry metrics were considered important in the models. Furthermore, because smoking was treated as a categorical (never, previous, current) rather than a continuous variable, the relationship between spirometry metrics and HF may have been influenced, potentially becoming more pronounced, as spirometry could partially reflect the cumulative effect of smoking.

The most important blood biomarkers, in both sexes, were cystatin-C, CRP and RDW while HbA1c was important only in men. Cystatin-C has been identified as a strong, independent prognostic marker of incident HF in the elderly general population.^28^ Cystatin-C serves as a marker of renal impairment, which is strongly associated with HF. Moreover, higher cystatin-C levels may be associated with HF risk factors such as hyperhomocysteinemia.^29^ In our study, cystatin-C was a better predictor of HF than creatinine. This could be due to the fact that cystatin-C reflects kidney function more accurately, independent of muscle mass, unlike creatinine, which is influenced by muscle tissue. Moreover, cystatin-C increases early in kidney dysfunction, providing a sensitive marker. Additionally, cystatin-C is linked to inflammation and cardiovascular risk.^30^ CRP is a strong predictor of incident HF and adverse events in established HF, in both healthy populations and among patients with various comorbidities such as atherosclerosis, acute myocardial infarction (MI) or diabetes.^31^ In the Rotterdam Study, CRP was found to be a strong independent HF predictor, although this relationship was more pronounced in men.^32^ These observations contradict our findings which suggest similar importance of CRP in both sexes. Red blood cell distribution width, which reflects the rate of anisocytosis, has been associated with various cardiovascular outcomes including HF over the last decade.^33,34^ HbA1c is closely associated with diabetes mellitus^35^ and our results suggest that HbA1c is only associated with incident HF in men. Previous research has suggested that HbA1c tends to underestimate fasting glucose in men and, consequently, results in a larger liability of diabetes compared with women.^36^ The same study suggested that similar levels of HbA1c represent higher levels of glycaemia in men compared with women, which in turn can lead to underdiagnosis and undertreatment. Diabetes in turn confers a higher absolute risk of coronary heart disease in men than women despite similar relative risks.^36^

When expanding the scope beyond the top 15 predictors, reduced serum albumin levels were much more important for incident HF risk in men than women, occurring in three models in men vs. zero in women. Reduced albumin levels have been associated with an increased risk for hospitalization and mortality in chronic HF patients^37,38^ although these studies did not find significant differences between sexes. Studies on the Atherosclerosis Risk in Communities study found associations of albuminuria^39^ and an inverse relationship between albumin volume^40^ on incident HF risk. Neither studies considered sex differences, thus our findings extend these results with the notion that reduced serum albumin appears to be more important in men than women for incident HF risk.

Hypertension is a well-known risk factor for HF in women. In the Framingham heart study,^8^ although the prevalence of hypertension was similar amongst sexes, associated HF risk was greater in hypertensive women than men. This concurs with our study where, even though men have overall higher systolic blood pressure (SBP) values and more often use BP medication, BP was associated with incident HF risk in both sexes.

Our study has several strengths and limitations. The UK Biobank is well-established, clearly documented, features a large general population and contains an extensive number of predictor variables. Another strength is the inclusion of LASSO stability selection which, to our knowledge, has found little use in the incident HF setting and is easy to incorporate in clinical practice. A limitation is the absence of measurements of N-terminal pro B-type natriuretic peptide (NT-proBNP) and troponin, both well-known prognostic markers of HF. Including these markers could have influenced our results. Furthermore, we were limited by the parameters available in the UK Biobank; although this is a rather extensive set. The UK Biobank population also suffers from a ‘healthy’ participant bias^41^ and is predominantly white. Moreover, we were unable to examine time-dependent variable effects on incident HF risk. We also could not discern between HF phenotypes (HFrEF vs. HFpEF), since in the UK Biobank data, type of HF was not provided. Finally, since we censored participants at the moment mortality occurred, we did not account for the competing risk of death, potentially overestimating the incidence of nonfatal HF. However, in the context of several ML models, including XGBoost, incorporating competing risks is challenging due to the complexity of model adaptation and the need for specialized techniques. Although our results should be interpreted while keeping this issue in mind, they still offer additional insights into factors associated with risk of HF.

## Conclusions

Machine learning models showed similar performance to traditional Cox PH models for HF prediction in women and men without a history of HF, IHD and CM at baseline. However, both sex-specific and model-specific risk predictors were found. Spirometry metrics, not commonly included in existing models, were identified as important risk factors. As such, the ML models indicate the potential value of HF risk predictors normally excluded from traditional HF risk prediction models.

## Lead author biography


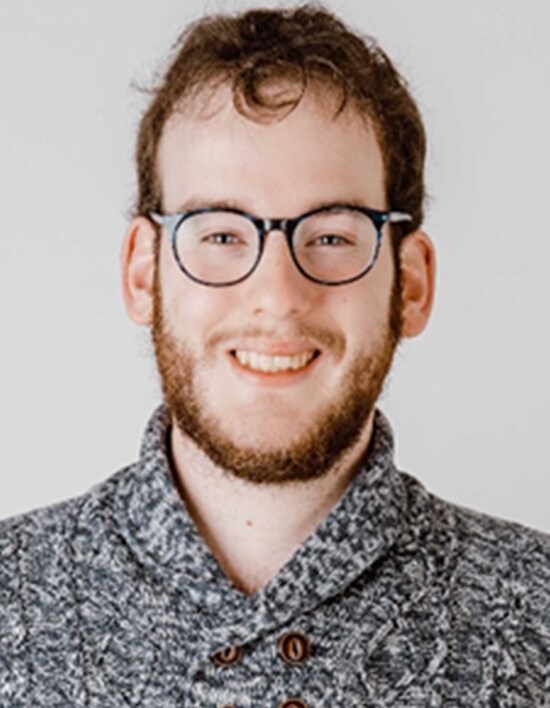


Thomas F. Kok is a PhD student at the Department of Cardiology at the Erasmus Medical Center in Rotterdam, the Netherlands. His main research interests are clinical epidemiology of heart failure (HF); the early assessment of HF risk in general population cohort studies, as well as risk of adverse events in patients with HF; the role of serially measured blood biomarkers in HF; and application of machine learning techniques.

## Supplementary Material

ztaf118_Supplementary_Data

## Data Availability

Access to the data can be requested directly at the UK Biobank, via the route as installed by the UK Biobank. If the request is approved, all necessary data will be shared as provided by the UK Biobank. Prevalent medical history variables, obtained through available ICD9 and ICD10 diagnoses codes, can be accessed upon request.

## Code availability

Any code pertaining to data cleaning, hyperparameter optimization, model building and validation, can be accessed upon request. The beta-coefficients and 10 year HF baseline hazard risks of both the ordinary and LASSO stabilized Cox models can be found at the end of the Supplementary material.
