# Supplementary material for: High-dimensional machine learning models for prediction of heart failure in more than 400 000 men and women from the UK Biobank
Source: Eur Heart J Digit Health. 2025 Oct 6;6(6):1234–45. doi: 10.1093/ehjdh/ztaf118 (PMC12629653; doi:10.1093/ehjdh/ztaf118)
Supplement: ztaf118_Supplementary_Data [file ztaf118_supplementary_data.docx]

Supplement: Table of Contents

**Missingness and data aggregation** 2

**Recategorization** 2

**Calibration details** 2

**Variable overview** 2

Table S1: Overview of included additional exposures variables. 3

Table S2: Overview of biological sample variables. 4

Table S3: Overview of early life factor variables. 6

Table S4: Overview of family history variables. 6

Table S5: Overview of health and medical history variables. 6

Table S6: Overview of lifestyle and environment variables. 9

Table S7: Overview of physical measures variables. 12

Table S8: Overview of population characteristics variables. 14

Table S9: Overview of psychosocial factor variables. 14

Table S10: Overview of female specific factor variables. 15

Table S11: Overview of sociodemographics variables. 16

Table S12: Overview of verbal interview variables. 17

Table S13: Overview of available prevalent ICD10-diagnosis codes, stored in
field p41280_i0 and their assigned respective binary data-column,
used as medical history variables. 17

Table S14: Participant number of all train- and hold-out sets for men and women. 18

Table S15: Missing data, stratified by sex, for each predictor variable. 19

**Hyper parameter tuning in the ML models.** 28

Table S16: Explored hyperparameter values for Random Survival Forest and XGBoost. 28

Table S17: Baseline characteristics of the subset population (N = 35,535),
used for variable importance assessment. 29

Table S18: UK Biobank baseline characteristics, stratified by incident HF event status**.** 30

Table S19: Baseline characteristics of men, stratified by incident HF event status. 31

Table S20: Baseline characteristics of women, stratified by incident HF event status. 32

Table S21: Performance scores for UK Biobank data in PCP-HF score. 33

Table S22: P-values and importance scores for the 15 most important predictor variables in subset men. 33

Table S23: P-values and importance scores for the 15 most important predictor variables in subset women. 33

Table S24: Remaining numbers of predictors after LASSO stability selection in the full datasets and subsets. 34

Table S25: Beta estimates of LASSO Cox models. 34

Table S26: Beta estimates of randomized LASSO Cox models. 35

Table S27: Beta estimates of ordinary Cox models. 36

Table S28: Performance metrices of ML models trained on variables remaining after LASSO stability selection. 58

Figure S1: Overview of data flow. 59

Figure S2: Net benefit analysis for men in the subset. 60

Figure S3: Net benefit analysis for women in the subset 60

Figure S4: Calibration plots for subset men. 61

Figure S5: Calibration plots for subset women. 61

Figure S6: Permutation importance for RSF, stratified by sex, in the subset data. 62

Figure S7: Permutation importance for XGBoost, stratified by sex, in the subset data. 63

Figure S8: Correlation plots of high-risk variables as identified in men. 64

Figure S9: Correlation plots of high-risk variables as identified in women. 65

**Supplement**

**Missingness and data aggregation**

Data was extracted from the Research Analysis Platform, enabled by DNAnexus technology and powered by Amazon Web Services (AWS). A total of 25,576 data columns were extracted in separate data frames of various sizes, ranging from single- to thousand column datasets. The extraction included baseline- and repeat assessment visit data as well as imaging project data.

Many variables showed large missing data percentages. We sought to define a missing data cutoff percentage for which as many informative variables would remain. For this purpose, we defined a subgroup of UK Biobank participants ( n = 63,840) that included all participants with inpatient hospital records, both in the primary and secondary position, with an ICD10 code related to angina pectoris (I20), acute- or subsequent myocardial infarction (I21-I22), complications following acute myocardial infarction (I23), other acute ischemic heart diseases (I24) and chronic ischemic heart disease (I25). The subgroup also included all participants with cardiomyopathy (I42), cardiomyopathy in diseases classified elsewhere (I43) and HF (I50).

Missing data percentages were calculated for all data columns in the subset. Variables with less than 50% missing data were selected and the corresponding missing percentages assessed in the full dataset. A maximum allowed missingness criterion of 54% was found to ensure that no informative variables were lost. After manual review, this criterion was lowered to 50% since only 3 additional variables were removed which were not considered informative.

All separate datasets were aggregated in one final dataset. Despite adhering to a missing data criterion, a fraction of remaining variables had to be removed due to their data structure, complexity or lack of predictive relevance.

**Recategorization**

To lower data complexity for imputation purposes, variables that consisted of cells with multiple available answers, have been simplified. As an example, we take variable p6179_i0, which describes whether participants used mineral or other dietary supplements. This data column consisted of 66 unique categories, even though only 8 options were possible. This stems from the fact that participants could provide multiple answers. These variables have been reworked such that, for each option, a separate binary column is created where 0 = no and 1 = yes for that particular dietary supplement. The following variables were recategorized like this: p6139_i0, p6146_i0,p6149_i0, p6150_i0, p6152_i0, p6153_i0_i0, p6154_i0, p6155_i0, p6159_i0, p6160_i0, p6162_i0, p6164_i0, p6177_i0, and p6179_i0.

Furthermore, variable p6138_i0 (Education) and p6142_i0 (Employment) were recategorized, yet kept as single columns. For p6138_i0, only the highest selected education was included. For p6142_i0, paid employment was prioritized, followed by retirement and unemployment. Because the UK Biobank is predominantly white, variable p21000_i0 (Ethnicity) was restructured so that ethnic categories, that made up less than 1% of the sex-specific study population, were combined into a group called “Other”. Smoking status (p20116_i0) was treated, as provided by the UK Biobank, as a tri-status variable with “Never”, “Previous” and “Current” serving as possible answers.

Finally, for many variables, participants could provide “Prefer not to answer” or “Do not know” as an answer. These answers were considered as missing and, subsequently, imputed.

**Calibration details**

Calibration plots were based on estimated event probabilities. For the Cox PH models and the XGBoost models, event probabilities were determined using a baseline hazard function based on the Kaplan-Meier estimator. For RSF, estimated event probabilities are based on an ensemble survival function derived from bootstrapped Kaplan-Meier estimators.

**Variable overview**

Each table in this overview shows variables of a particular category. The Field ID, representing how the data is originally stored in the UK Biobank dataset, is given in the first column. The second column displays the name assigned to the particular data-field during analysis. The third column provides a brief description of each variable.

Readers who wish to know more about specific variables, are recommended to search for the Field ID in the UK Biobank search bar <https://biobank.ndph.ox.ac.uk/ukb/search.cgi> which elaborates on the variable in question.

**Table S1: Overview of additional exposures variables.**

| Field ID | Code | Variable |
| --- | --- | --- |
| p24501_i0 | Domestic_garden_percentage_buffer_1000m | Domestic garden percentage, buffer 1000 m |
| p24502_i0 | Water_percentage_buffer_1000m | Water percentage, buffer 1000 m |
| p24503_i0 | Greenspace_percentage_buffer_300m | Greenspace percentage, buffer 300 m |
| p24504_i0 | Domestic_garden_percentage_buffer_300m | Domestic garden percentage, buffer 300 m |
| p24505_i0 | Water_percentage_buffer_300m | Water percentage, buffer 300 m |
| p24506_i0 | Natural_environment_percentage_buffer_1000m | Natural environment percentage, buffer 1000 m |
| p24507_i0 | Natural_environment_percentage_buffer_300m | Natural environment percentage, buffer 300 m |
| p24508_i0 | Euclidian_distance_coast | Distance (Euclidian) to coast |
| p24003 | Nitrogen_dioxide_air_pollution | Nitrogen dioxide air pollution; 2010 |
| p24004 | Nitrogen_oxides_air_pollution | Nitrogen oxides air pollution; 2010 |
| p24005 | Particulate_matter_air_pollution_pm10 | Particulate matter air pollution (pm10); 2010 |
| p24006 | Particulate_matter_air_pollution_pm2 | Particulate matter air pollution (pm2.5); 2010 |
| p24007 | Particulate_matter_air_pollution_pm2_absorbance | Particulate matter air pollution (pm2.5) absorbance |
| p24008 | Particulate_matter_air_pollution_2um_10um | Particulate matter air pollution (2.5-10 um); 2010 |
| p24009 | Traffic_intensity_nearest_road | Traffic intensity on nearest road |
| p24010 | Inverse_distance_nearest_road | Inverse distance to the nearest road |
| p24011 | Traffic_intensity_nearest_major_road | Traffic intensity on the nearest major road |
| p24012 | Inverse_distance_nearest_major_road | Inverse distance to the nearest major road |
| p24013 | Total_traffic_load_major_roads | Total traffic load on major roads |
| p24014 | Close_to_major_road | Close to major road |
| p24015 | Sum_road_length_major_roads | Sum of road length of major roads within 100m |
| p24016 | Nitrogen_oxides_air_pollution_2005 | Nitrogen dioxide air pollution; 2005 |
| p24017 | Nitrogen_oxides_air_pollution_2006 | Nitrogen dioxide air pollution; 2006 |
| p24018 | Nitrogen_oxides_air_pollution_2007 | Nitrogen dioxide air pollution; 2007 |
| p24019 | Particulate_matter_air_pollution_pm10_2007 | Particulate matter air pollution (pm10); 2007 |
| p24020 | Avg_daytime_sound_level_noise_pollution | Average daytime sound level of noise pollution |
| p24021 | Avg_evening_sound_level_noise_pollution | Average evening sound level of noise pollution |
| p24022 | Avg_night_time_sound_level_noise_pollution | Average night-time sound level of noise pollution |
| p24023 | Avg_16h_sound_level_noise_pollution | Average 16-hour sound level of noise pollution |
| p24024 | Avg_24h_sound_level_noise_pollution | Average 24-hour sound level of noise pollution |
| p24500_i0 | Greenspace_percentage_buffer_1000m | Greenspace percentage, buffer 1000 m |

**Table S2: Overview of biological sample variables.**

| Field ID | Code | Variable |
| --- | --- | --- |
| p30600_i0 | Albumin | Albumin (g/L) |
| p30610_i0 | Alkaline_phosphate | Alkaline phosphate (U/L) |
| p30620_i0 | Alanine_aminotransferase | Alanine aminotransferase (U/L) |
| p30630_i0 | Apoliprotein_A | Apoliprotein A (g/L) |
| p30640_i0 | Apoliprotein_B | Apoliprotein B (g/L) |
| p30650_i0 | Aspartate_aminotransferase | Aspartate aminotransferase (U/L) |
| p30660_i0 | Direct_bilirubin | Direct bilirubin (umol/L) |
| p30670_i0 | Urea | Urea (mmol/L) |
| p30680_i0 | Calcium | Calcium (mmol/L) |
| p30690_i0 | Cholesterol | Cholesterol (mmol/L) |
| p30700_i0 | Creatinine | Creatinine (umol/L) |
| p30710_i0 | C_reactive_protein | C-reactive protein (mg/L) |
| p30720_i0 | Cystatin_C | Cystatin C (mg/L) |
| p30730_i0 | Gamma_glutamyltransferase | Gamma glutamyltransferase (U/L) |
| p30740_i0 | Glucose | Glucose (mmol/L) |
| p30750_i0 | Glycated_haemoglobin | Glycated haemoglobin (HbA1c) (mmol/mol) |
| p30760_i0 | HDL_cholesterol | HDL cholesterol (mmol/L) |
| p30770_i0 | IGF_1 | IGF-1 (nmol/L) |
| p30780_i0 | LDL_direct | LDL direct (mmol/L) |
| p30790_i0 | Lipoprotein_A | Lipoprotein A (nmol/L) |
| p30810_i0 | Phosphate | Phosphate (mmol/L) |
| p30830_i0 | SHGB | SHBG (nmol/L) |
| p30840_i0 | Total_bilirubin | Total bilirubin (umol/L) |
| p30850_i0 | Testosterone | Testosterone (nmol/L) |
| p30860_i0 | Total_protein | Total protein (g/L) |
| p30870_i0 | Triglycerides | Triglycerides (mmol/L) |
| p30880_i0 | Urate | Urate (umol/L) |
| p30890_i0 | Vitamin_D | Vitamin D (nmol/L) |
| p30000_i0 | White_blood_cell_count | White blood cell (leukocyte) count (10^9^ cells/L) |
| p30010_i0 | Red_blood_cell_count | Red blood cell (erythrocyte) count (10^12^ cells/L) |
| p30020_i0 | Haemoglobin_concentration | Haemoglobin concentration (g/dL) |
| p30030_i0 | Haematocrit_percentage | Haematocrit concentration (%) |
| p30040_i0 | Mean_corpuscular_volume | Mean corpuscular volume (10^-15^ L) |
| p30050_i0 | Mean_corpuscular_haemoglobin | Mean corpuscular haemoglobin count (10^-12^ g) |
| p30060_i0 | Mean_corpuscular_haemoglobin_conc | Mean corpuscular haemoglobin concentration (g/dL) |
| p30070_i0 | Red_blood_cell_distribution_width | Red blood cell (erythrocyte) distribution width (%) |
| p30080_i0 | Platelet_count | Platelet count (10^9^ cells/L) |
| p30090_i0 | Platelet_crit | Platelet crit (%) |
| p30100_i0 | Mean_platelet_volume | Mean platelet (thrombocyte) volume (10^-15^ L) |
| p30110_i0 | Platelet_distribution_width | Platelet distribution width (%) |
| p30120_i0 | Lymphocyte_count | Lymphocyte count (10^9^ cells/L) |
| p30130_i0 | Monocyte_count | Monocyte count (10^9^ cells/L) |
| p30140_i0 | Neutrophil_count | Neutrophil count (10^9^ cells/L) |
| p30150_i0 | Eosinophil_count | Eosinophil count (10^9^ cells/L) |
| p30160_i0 | Basophil_count | Basophil count (10^9^ cells/L) |
| p30170_i0 | Nucleated_red_blood_cell_count | Nucleated red blood cell count (10^9^ cells/L) |
| p30180_i0 | Lymphocyte_percentage | Lymphocyte percentage (%) |
| p30190_i0 | Monocyte_percentage | Monocyte percentage (%) |
| p30200_i0 | Neutrophil_percentage | Neutrophil percentage (%) |
| p30210_i0 | Eosinophil_percentage | Eosinophil percentage (%) |
| p30220_i0 | Basophil_percentage | Basophil percentage (%) |
| p30230_i0 | Nucleated_red_blood_cell_percentage | Nucleated red blood cell percentage (%) |
| p30240_i0 | Reticulocyte_percentage | Reticulocyte percentage (%) |
| p30250_i0 | Reticulocyte_count | Reticulocyte count (10^12^ cells/L) |
| p30260_i0 | Mean_reticulocyte_volume | Mean reticulocyte volume (10^-15^ L) |
| p30270_i0 | Mean_sphered_cell_volume | Mean sphered cell volume (10^-15^ L) |
| p30280_i0 | Immature_reticulocyte_fraction | Immature reticulocyte fraction |
| p30290_i0 | High_light_scatter_reticulocyte_percentage | High light scatter reticulocyte percentage (%) |
| p30300_i0 | High_light_scatter_reticulocyte_count | High light scatter reticulocyte count (10^12^ cells/L) |
| p74_i0 | Fasting_time | Fasting time |
| p30510_i0 | Creatinine_in_urine | Creatinine (enzymatic) in urine (mmol/L) |
| p30520_i0 | Potassium_in_urine | Potassium in urine (mmol/L) |
| p30530_i0 | Sodium_in_urine | Sodium in urine (mmol/L) |

**Table S3: Overview of early life factor variables.**

| Field ID | Code | Variable |
| --- | --- | --- |
| p1647_i0 | Country_of_birth | Country of birth (UK/elsewhere) |
| p1677_i0 | Breastfed_as_baby | Breastfed as a baby |
| p1687_i0 | Comp_body_size_age10 | Comparative body size at age 10 |
| p1697_i0 | Comp_height_size_age10 | Comparative height size at age 10 |
| p1707_i0 | Handedness | Handedness (chirality/laterality) |
| p1767_i0 | Adopted_as_child | Adopted as a child |
| p1777_i0 | Part_of_multiple_birth | Part of a multiple birth |
| p1787_i0 | Maternal_smoking_around_birth | Maternal smoking around birth |

**Table S4: Overview of family history variables.**

| Field ID | Code | Variable |
| --- | --- | --- |
| p1797_i0 | Father_still_alive | Father still alive |
| p1807_i0 | Father_age_at_death | Fathers age at death |
| p1835_i0 | Mother_still_alive | Mother still alive |
| p1873_i0 | Full_brothers | Number of full brothers |
| p1883_i0 | Full_sisters | Number of full sisters |
| p3526_i0 | Mothers_age_at_death | Mother's age at death |

**Table S5: Overview of health and medical history variables.**

| Field ID | Code | Variable |
| --- | --- | --- |
| p2316_i0 | Wheeze_whistle_chest_1y | Wheeze or whistling in the chest in last year |
| p2345_i0 | Ever_had_bowel_cancer_screening | Ever had bowel cancer screening |
| p2335_i0 | Chest_pain | Chest pain or discomfort |
| p2207_i0 | Wears_glasses_contacts | Wears glasses or contact lenses |
| p2217_i0 | Age_started_wearing_glass_contacts | Age started wearing glasses or contact lenses |
| p2227_i0 | Other_eye_problems | Other eye problems |
| p2178_i0 | Overall_health_rating | Overall health rating |
| p2296_i0 | Falls_last_year | Falls in the last year |
| p2306_i0 | Weight_change_comp_1y | Weight change compared to 1 year ago |
| p2188_i0 | Long_standing_illness | Long-standing illness, disability or infirmity |
| p2247_i0 | Hearing_difficulty | Hearing difficulty/problems |
| p2257_i0 | Hearing_difficulty_background_noise | Hearing difficulty/problems with background noise |
| p3393_i0 | Hearing_aid_user | Hearing aid user |
| p2443_i0 | Diabetes_diag_by_doctor | Diabetes diagnosed by doctor |
| p2453_i0 | Cancer_diag_by_doctor | Cancer diagnosed by doctor |
| p2463_i0 | Fractured_bones_last_5years | Fractured/broken bones in last 5 years |
| p2473_i0 | Other_serious_med_condition_diag_by_doctor | Other medical condition/disability diagnosed by doctor |
| p6150_i0 | p6150_i0Angina | Angina diagnosed by doctor |
| p6150_i0 | p6150_i0Heart_attack | Heart attack diagnosed by doctor |
| p6150_i0 | p6150_i0High_blood_pressure | High blood pressure diagnosed by doctor |
| p6150_i0 | p6150_i0Stroke | Stroke diagnosed by doctor |
| p6152_i0 | p6152_i0Asthma | Asthma diagnosed by doctor |
| p6152_i0 | p6152_i0Blood_clot_in_the_leg_DVT | Blood clot in the leg (DVT) diagnosed by doctor |
| p6152_i0 | p6152_i0Blood_clot_in_the_lung | Blood clot in the lung diagnosed by doctor |
| p6152_i0 | p6152_i0Emphysema_chronic_bronchitis | Emphysema chronic bronchitis diagnosed by doctor |
| p6152_i0 | p6152_i0Hayfever_allergic_rhinitis_or_eczema | Hayfever allergy, rhinitis or eczema diagnosed by doctor |
| p6153_i0 | p6153_i0Blood_pressure_medication | Blood pressure medication use: women only |
| p6153_i0 | p6153_i0Cholesterol_lowering_medication | Cholesterol lowering medication use: women only |
| p6153_i0 | p6153_i0Hormone_replacement_therapy | Hormone replacement therapy use: women only |
| p6153_i0 | p6153_i0Insulin | Insulin use: women only |
| p6153_i0 | p6153_i0Oral_contraceptive_pill_or_minipill | Oral contraceptive pill or mini pill usage: women only |
| p2492_i0 | Taking_other_prescription_medications | Taking other prescription medications |
| p6154_i0 | p6154_i0Aspirin | Aspirin use |
| p6154_i0 | p6154_i0Ibuprofen_eg_Nurofen | Ibuprofen (e.g. Nurofen) use |
| p6154_i0 | p6154_i0Laxatives_eg_Dulcolax__Senokot | Laxatives (e.g. Dulcolax, Senokot) use |
| p6154_i0 | p6154_i0Omeprazole_eg_Zanprol | Omeprazole (e.g. Zanprol) use |
| p6154_i0 | p6154_i0Paracetamol | Paracetamol use |
| p6154_i0 | p6154_i0Ranitidine_eg_Zantac | Rantidine (e.g. Zantac) use |
| p6155_i0 | p6155_i0Folic_acid_or_Folate_Vit_B9 | Folic acid or Folate (Vit B9) use |
| p6155_i0 | p6155_i0Multivitamins___minerals | Multivitamins +/- minerals use |
| p6155_i0 | p6155_i0Vitamin_A | Vitamin A supplement use |
| p6155_i0 | p6155_i0Vitamin_B | Vitamin B supplement use |
| p6155_i0 | p6155_i0Vitamin_C | Vitamin C supplement use |
| p6155_i0 | p6155_i0Vitamin_D | Vitamin D supplement use |
| p6155_i0 | p6155_i0Vitamin_E | Vitamin E supplement use |
| p6179_i0 | p6179_i0Calcium | Calcium supplement use |
| p6179_i0 | p6179_i0Fish_oil_including_cod_liver_oil | Fish oil (including cod liver oil) supplement use |
| p6179_i0 | p6179_i0Glucosamine | Glucosamine supplement use |
| p6179_i0 | p6179_i0Iron | Iron supplement use |
| p6179_i0 | p6179_i0Selenium | Selenium supplement use |
| p6179_i0 | p6179_i0Zinc | Zinc supplement use |
| p6177_i0 | p6177_i0Blood_pressure_medication | Blood pressure medication use: men only |
| p6177_i0 | p6177_i0Cholesterol_lowering_medication | Cholesterol lowering medication use: men only |
| p6177_i0 | p6177_i0Insulin | Insulin use: men only |
| p6149_i0 | p6149_i0Bleeding_gums | Self-reported presence of bleeding gums |
| p6149_i0 | p6149_i0Dentures | Self-reported presence of dentures |
| p6149_i0 | p6149_i0Loose_teeth | Self-reported presence of loose teeth |
| p6149_i0 | p6149_i0Mouth_ulcers | Self-reported presence of mouth ulcers |
| p6149_i0 | p6149_i0Painful_gums | Self-reported presence of painful gums |
| p6149_i0 | p6149_i0Toothache | Self-reported presence of toothache |
| p2844_i0 | Had_other_major_operations | Had other major operations |
| p6159_i0 | p6159_i0Back_pain | Pain experienced in last month: Back pain |
| p6159_i0 | p6159_i0Facial_pain | Pain experienced in last month: Facial pain |
| p6159_i0 | p6159_i0Headache | Pain experienced in last month: Headache pain |
| p6159_i0 | p6159_i0Hip_pain | Pain experienced in last month: Hip pain |
| p6159_i0 | p6159_i0Knee_pain | Pain experienced in last month: Knee pain |
| p6159_i0 | p6159_i0Neck_or_shoulder_pain | Pain experienced in last month: Neck/shoulder pain |
| p6159_i0 | p6159_i0Pain_all_over_the_body | Pain experienced in last month: Pain all over body |
| p6159_i0 | p6159_i0Stomach_or_abdominal_pain | Pain experienced in last month: Stomach/abdominal pain |

**Table S6: Overview of lifestyle and environment variables.**

| Field ID | Code | Variable |
| --- | --- | --- |
| p1558_i0 | Alcohol_intake_frequency | Alcohol intake frequency |
| p1568_i0 | Avg_weekly_red_wine_intake | Average weekly red wine intake |
| p1578_i0 | Avg_weekly_white_wine_intake | Average weekly champagne plus white wine intake |
| p1588_i0 | Avg_weekly_beer_intake | Average weekly beer plus cider intake |
| p1598_i0 | Avg_weekly_spirit_intake | Average weekly spirits intake |
| p1608_i0 | Avg_weekly_fort_wine_intake | Average weekly fortified wine intake |
| p1618_i0 | Alcohol_between_meals | Alcohol usually taken with meals |
| p1628_i0 | Alcohol_intake_vs_10years_ago | Alcohol intake versus 10 years previously |
| p20117_i0 | Alcohol_drinker_status | Alcohol drinker status |
| p1289_i0 | Cooked_vegetable_intake | Cooked vegetable intake |
| p1299_i0 | Salad_raw_vegetable_intake | Salad/raw vegetable intake |
| p1309_i0 | Fresh_fruit_intake | Fresh fruit intake |
| p1319_i0 | Dried_fruit_intake | Dried fruit intake |
| p1329_i0 | Oily_fish_intake | Oily fish intake |
| p1339_i0 | Non_oily_fish_intake | Non-oily fish intake |
| p1349_i0 | Processed_meat_intake | Processed meat intake |
| p1359_i0 | Poultry_intake | Poultry intake |
| p1369_i0 | Beef_intake | Beef intake |
| p1379_i0 | Lamb_mutton_intake | Lamb/mutton intake |
| p1389_i0 | Pork_intake | Pork intake |
| p1408_i0 | Cheese_intake | Cheese intake |
| p1418_i0 | Milk_type_used | Milk type used |
| p1428_i0 | Spread_type | Spread type |
| p1438_i0 | Bread_intake | Bread intake |
| p1448_i0 | Bread_type | Bread type |
| p1458_i0 | Cereal_intake | Cereal intake |
| p1468_i0 | Cereal_type | Cereal type |
| p1478_i0 | Salt_added_to_food | Salt added to food |
| p1488_i0 | Tea_intake | Tea intake |
| p1498_i0 | Coffee_intake | Coffee intake |
| p1508_i0 | Coffee_type | Coffee type |
| p1518_i0 | Hot_drink_temp | Hot drink temperature |
| p1528_i0 | Water_intake | Water intake |
| p1538_i0 | Diet_changes_last_5y | Major dietary changes in the last five years |
| p1548_i0 | Variation_in_diet | Variation in diet |
| p1110_i0 | Length_mobile_phone_use | Length of mobile phone use |
| p1120_i0 | Weekly_use_mobile_phone_3_months | Weekly usage of mobile phone in last 3 months |
| p1130_i0 | Hands_free_speakerphone_use_3_months | Hands-free device/speakerphone use with mobile phone in last three months |
| p1140_i0 | Mobile_phone_use_2_years_diff | Difference in mobile phone use compared to two years previously |
| p1150_i0 | Usual_side_head_phone_use | Usual side of head for mobile phone use |
| p2237_i0 | Plays_computer_games | Plays computer games |
| p22033_i0 | Summed_days_activity | Summed days activity |
| p22034_i0 | Summed_minutes_activity | Summed minutes activity |
| p22037_i0 | MET_minutes_per_week_of_walking | MET minutes per week for walking |
| p22038_i0 | MET_minutes_per_week_of_mod_activity | MET minutes per week for moderate activity |
| p22039_i0 | MET_minutes_per_week_of_vigor_activity | MET minutes per week for vigorous activity |
| p22040_i0 | Summed_MET_minutes_per_week_all_activity | Summed MET minutes per week for all activity |
| p1070_i0 | Time_watching_television | Time spent watching television (TV) |
| p1080_i0 | Time_using_computer | Time spent using computer |
| p1090_i0 | Time_spent_driving | Time spent driving |
| p1100_i0 | Drive_faster_speed_limit | Drive faster than motorway speed limit |
| p864_i0 | Days_week_walked_10_min | Number of days/week walked 10+ minutes |
| p874_i0 | Duration_walks | Duration of walks |
| p884_i0 | Days_per_week_mod_phys_activity_10_min | Number of days/week of moderate physical activity 10+ minutes |
| p894_i0 | Duration_moderate_activity | Duration of moderate activity |
| p904_i0 | Days_per_week_vigor_phys_activity_10_min | Number of days/week of vigorous physical activity 10+ minutes |
| p914_i0 | Duration_vigorous_activity | Duration of vigorous activity |
| p924_i0 | Usual_walking_pace | Usual walking pace |
| p943_i0 | Freq_stair_climbing | Frequency of stair climbing in last 4 weeks |
| p971_i0 | Freq_walking_pleasure | Frequency of walking for pleasure in last 4 weeks |
| p981_i0 | Duration_walking_pleasure | Duration walking for pleasure |
| p6162_i0 | p6162_i0Car_motor_vehicle | Types of transport used (excluding work): Car/motor |
| p6162_i0 | p6162_i0Cycle | Types of transport used (excluding work): Cycle |
| p6162_i0 | p6162_i0Public_transport | Types of transport used (excluding work): Public transport |
| p6162_i0 | p6162_i0Walk | Types of transport used (excluding work): Walk |
| p6164_i0 | p6164_i0Heavy_DIY_eg_weeding__lawn  _mowing__carpentry__digging | Type of physical activity in last 4 weeks: Heavy DIY |
| p6164_i0 | p6164_i0Light_DIY_eg_pruning__  watering_the_lawn | Type of physical activity in last 4 weeks: Light DIY |
| p6164_i0 | p6164_i0Other_exercises_eg_  swimming__cycling__keep_fit__bowling | Type of physical activity in last 4 weeks: Other exercises |
| p6164_i0 | p6164_i0Strenuous_sports | Type of physical activity in last 4 weeks: Strenuous sports |
| p6164_i0 | p6164_i0Walking_for_pleasure_not_as  _a_means_of_transport | Type of physical activity in last 4 weeks: Walking for pleasure |
| p2129_i0 | Answered_sexual_hist_questions | Answered sexual history questions |
| p2139_i0 | Age_first_sex_intercourse | Age first had sexual intercourse |
| p2149_i0 | Lifetime_sexual_partners | Lifetime number of sexual partners |
| p2159_i0 | Ever_had_same_sex_intercourse | Ever had same-sex intercourse |
| p1160_i0 | Sleep_duration | Average sleep duration |
| p1170_i0 | Getting_up_morning | Ease of getting up in morning |
| p1180_i0 | Morning_evening_person | Morning/evening person (chronotype) |
| p1190_i0 | Nap_during_day | Nap during day |
| p1200_i0 | Insomnia | Sleeplessness/insomina |
| p1210_i0 | Snoring | Snoring |
| p1220_i0 | Daytime_sleeping | Daytime dozing/sleeping (narcolepsy) |
| p1239_i0 | Current_tobacco_smoking | Current tobacco smoking |
| p1249_i0 | Past_tobacco_smoking | Past tobacco smoking |
| p1259_i0 | Smoking_smokers_household | Smoking/smokers in household |
| p1269_i0 | Exposure_to_tobacco_at_home | Exposure to tobacco smoke at home |
| p1279_i0 | Exposure_to_tobacco_outside_home | Exposure to tobacco smoke outside home |
| p20116_i0 | Smoking_status | Smoking status |
| p1050_i0 | Time_outdoors_summer | Time spend outdoors in summer |
| p1060_i0 | Time_outdoors_winter | Time spend outdoors in winter |
| p1717_i0 | Skin_colour | Skin colour |
| p1727_i0 | Ease_skin_tanning | Ease of skin tanning |
| p1737_i0 | Childhood_sunburn_occasions | Childhood sunburn occasions |
| p1757_i0 | Facial_ageing | Facial ageing |
| p2267_i0 | Use_of_sun_protection | Use of sun/uv protection |
| p2277_i0 | Freq_use_sunlamp | Frequency of solarium/sunlamp use |
| p1747_i0 | Hair_colour_natural | Hair colour (natural, before greying) |

**Table S7: Overview of physical measures variables.**

| Field ID | Code | Variable |
| --- | --- | --- |
| p4079_i0_a0 | Diastolic_blood_pressure | Diastolic blood pressure, automated reading |
| p4080_i0_a0 | Systolic_blood_pressure | Systolic blood pressure, automated reading |
| p23098_i0 | Weight_impedance_measurement | Weight |
| p23099_i0 | Body_fat_percentage | Body fat percentage |
| p23100_i0 | Whole_body_fat_mass | Whole body fat mass |
| p23101_i0 | Whole_body_fat_free_mass | Whole body fat-free mass |
| p23102_i0 | Whole_body_water_mass | Whole body water mass |
| p23104_i0 | Body_mass_index | Body mass index (BMI) |
| p23105_i0 | Basal_metabolic_rate | Basal metabolic rate |
| p23106_i0 | Impedance_whole_body | Impedance of whole body |
| p23107_i0 | Impedance_right_leg | Impedance of leg (right) |
| p23108_i0 | Impedance_left_leg | Impedance of leg (left) |
| p23109_i0 | Impedance_right_arm | Impedance of arm (right) |
| p23110_i0 | Impedance_left_arm | Impedance of arm (left) |
| p23111_i0 | Right_leg_fat_percentage | Leg fat percentage (right) |
| p23112_i0 | Right_leg_fat_mass | Leg fat mass (right) |
| p23113_i0 | Right_leg_fatfree_mass | Let fat-free mass (right) |
| p23114_i0 | Right_leg_predicted_mass | Leg predicted mass (right) |
| p23115_i0 | Left_leg_fat_percentage | Leg fat percentage (left) |
| p23116_i0 | Left_leg_fat_mass | Leg fat mass (left) |
| p23117_i0 | Left_leg_fatfree_mass | Leg fat-free mass (left) |
| p23118_i0 | Left_leg_predicted_mass | Leg predicted mass (left) |
| p23119_i0 | Right_arm_fat_percentage | Arm fat percentage (right) |
| p23120_i0 | Right_arm_fat_mass | Arm fat mass (right) |
| p23121_i0 | Right_arm_fatfree_mass | Arm fat-free mass (right) |
| p23122_i0 | Right_arm_predicted_mass | Arm predicted mass (right) |
| p23123_i0 | Left_arm_fat_percentage | Arm fat percentage (left) |
| p23124_i0 | Left_arm_fat_mass | Arm fat mass (left) |
| p23125_i0 | Left_arm_fatfree_mass | Arm fat-free mass (left) |
| p23126_i0 | Left_arm_predicted_mass | Arm predicted mass (left) |
| p23127_i0 | Trunk_fat_percentage | Trunk fat percentage |
| p23128_i0 | Trunk_fat_mass | Trunk fat mass |
| p23129_i0 | Trunk_fatfree_mass | Trunk fat-free mass |
| p23130_i0 | Trunk_predicted_mass | Trunk predicted mass |
| p20015_i0 | Sitting_height | Sitting height |
| p21001_i0 | BMI | Body mass index (BMI) |
| p21002_i0 | Weight | Weight |
| p48_i0 | Waist_circumference | Waist circumference |
| p49_i0 | Hip_circumference | Hip circumference |
| p50_i0 | Standing_height | Standing height |
| p51_i0 | Seated_height | Seated height |
| p3143_i0 | Ankle_space_width | Ankle spacing width |
| p3146_i0 | Speed_of_sound_through_heel | Speed of sound through heel |
| p3148_i0 | Heel_bone_mineral_density | Heel bone mineral density (BMD) |
| p78_i0 | Heel_bone_mineral_density_T_score | Heel bone mineral density (BMD) T-score, automated |
| p3144_i0 | Heel_broadband_ultrasound_attenuation | Heel Broadband ultrasound attenuation, direct entry |
| p3147_i0 | Heel_quantitative_ultrasound_index | Heel quantitative ultrasound index (QUI), direct entry |
| p46_i0 | Hand_grip_strength_left | Hand grip strength (left) |
| p47_i0 | Hand_grip_strength_right | Hand grip strength (right) |
| p20150_i0 | FEV1_score_best_measure | Forced expiratory volume in 1-second (FEV1) Best measure |
| p20153_i0 | FEV1_score_predicted | Forced expiratory volume in 1-second (FEV1) |
| p20256 | FEV1_Z_score | Forced expiratory volume in 1-second (FEV1) Z-score |
| p20258 | FEV1_FVC_ratio_Z_score | FEV1/FVC ratio Z-score |
| p3061_i0_a0 | Acceptability_blow_result | Acceptability of each blow result |
| p3062_i0_a0 | Forced_vital_capacity | Forced vital capacity (FVC) |
| p3063_i0_a0 | Forced_expiratory_volume_in_1_second | Forced expiratory volume in 1-second (FEV1) |
| p3064_i0_a0 | Peak_expiratory_flow | Peak expiratory flow (PEF) |
| p3088_i0 | Contra_indications_for_spirometry | Contra-indications for spirometry |
| p3089_i0 | Caffeine_drink_within_last_hour | Caffeine drink within last hour |
| p3090_i0 | Used_inhaler_last_hour | Used an inhaler for chest within last hour |
| p20151_i0 | FVC_best_measure | Forced vital capacity (FVC), best score |
| p20154_i0 | FEV1_score_predicted_percentage | Forced expiratory volume in 1-second (FEV1), predicted % |
| p20257 | FVC_Z_score | Forced vital capacity (FVC), Z-score |

**Table S8: Overview of population characteristics variables.**

| Field ID | Code | Variable |
| --- | --- | --- |
| p21022 | Age_recruitment | Age at recruitment |
| p31 | Sex | Sex |
| p34 | Year_of_birth | Year of Birth |
| p26410 | Index_Multiple_Deprivation_England | Index of Multiple Deprivation (England) |
| p26411 | Income_score_England | Income score (England) |
| p26412 | Employment_score_England | Employment score (England) |
| p26413 | Health_score_England | Health score (England) |
| p26414 | Education_score_England | Education score (England) |
| p26415 | Housing_score_England | Housing score (England) |
| p26416 | Crime_score_England | Crime score (England) |
| p26417 | Living_environment_score_England | Living environment score (England) |
| p21003_i0 | Age_attending_assess_centre | Age when attended assessment centre |

**Table S9: Overview of psychosocial factor variables.**

| Field ID | Code | Variable |
| --- | --- | --- |
| p1920_i0 | Mood_swings | Mood swings |
| p1930_i0 | Miserableness | Miserableness |
| p1940_i0 | Irritability | Irritability |
| p1950_i0 | Sensitivity | Sensitivity/hurt feelings |
| p1960_i0 | Fed_up_feelings | Fed-up feelings |
| p1970_i0 | Nervous_feelings | Nervous feelings |
| p1980_i0 | Worrier_anxious_feelings | Worrier/anxious feelings |
| p1990_i0 | Tense | Tense/'highly strung' |
| p2000_i0 | Worry_long_after_embass | Worry too long after embarrassment |
| p2010_i0 | Suffer_from_nerves | Suffer from 'nerves' |
| p20127_i0 | Neuroticism_score | Neuroticism score |
| p2030_i0 | Guilty_feelings | Guilty feelings |
| p2040_i0 | Risk_taking | Risk taking |
| p2050_i0 | Freq_depressed_moods_2_weeks | Frequency of depressed mood in last 2 weeks |
| p2060_i0 | Freq_disinterest_2_weeks | Frequency of enthusiasm/disinterest in last 2 weeks |
| p2070_i0 | Freq_tenseness_2_weeks | Frequency of tenseness/restlessness in last 2 weeks |
| p2080_i0 | Freq_tiredness_2_weeks | Frequency of tiredness/lethargy in last 2 weeks |
| p2090_i0 | Seen_doctor_for_nerves_depression | Seen doctor (GP) for nerves, anxiety, tension or depression |
| p2100_i0 | Seen_psych_for_nerves_depression | Seen a psychiatrist for nerves, anxiety, tension or depression |
| p2020_i0 | Loneliness_isolation | Do you often feel lonely? |
| p6145_i0 | p6145_i0Death_of_a_close_relative | Death of a close relative in last 2 years |
| p6145_i0 | p6145_i0Death_of_a_spouse_or_partner | Death of a spouse/partner in last 2 years |
| p6145_i0 | p6145_i0Financial_difficulties | Financial difficulties in last 2 years |
| p6145_i0 | p6145_i0Marital_separation_divorce | Marital separation/divorce in last 2 years |
| p6145_i0 | p6145_i0Serious_illness__injury_or_  assault_of_close_relative | Serious illness, injury or assault on close relative in last 2 years |
| p6145_i0 | p6145_i0Serious_illness__injury_or_  assault_to_yourself | Serious illness, injury or assault on yourself in last 2 years |
| p1031_i0 | Freq_friend_family_visit | Frequency of friend/family visit |
| p2110_i0 | Able_to_confide | Able to confide |
| p6160_i0 | p6160_i0Adult_education_class | Attend adult education class at least once a week |
| p6160_i0 | p6160_i0Other_group_activity | Attend other group activity at least once a week |
| p6160_i0 | p6160_i0Pub_or_social_club | Attend pub or social club at least once a week |
| p6160_i0 | p6160_i0Religious_group | Attend religious group at least once a week |
| p6160_i0 | p6160_i0Sports_club_or_gym | Attend sports club or gym at least once a week. |

**Table S10: Overview of female specific factor variables.**

| Field ID | Code | Variable |
| --- | --- | --- |
| p2674_i0 | Ever_had_breast_cancer_screening | Ever had breast cancer screening/mammogram |
| p2694_i0 | Ever_had_cervical_smear_test | Ever had cervical smear test |
| p2714_i0 | Age_when_period_started | Age when periods started (menarche) |
| p2724_i0 | Had_menopause | Had menopause |
| p2734_i0 | Number_of_live_births | Number of live births |
| p2784_i0 | Ever_had_oral_contraceptive_pill | Ever taken oral-contraceptive pill |
| p2814_i0 | Ever_used_HRT | Ever used hormone replacement therapy (HRT) |
| p2834_i0 | Bilateral_oophorectomy | Bilateral oophorectomy (both ovaries removed) |
| p2774_i0 | Ever_had_stillbirth_miscarriage_abortion | Ever had stillbirth, spontaneous miscarriage or termination |

**Table S11: Overview of sociodemographics variables.**

| Field ID | Code | Variable |
| --- | --- | --- |
| p6138_i0 | Qualifications | Qualifications on education |
| p845_i0 | Age_comp_fulltime_education | Age completed full-time education |
| p6142_i0 | Current_employment_status | Current employment status |
| p757_i0 | Time_at_current_job | Time employed in main current job |
| p767_i0 | Length_work_week | Length of working week for main job |
| p777_i0 | Freq_travel_home_to_job | Frequency of travelling from home to job workplace |
| p806_i0 | Job_major_walking_standing | Job involves mainly walking or standing |
| p816_i0 | Job_involves_heavy_labor | Job involves heavy manual or physical work |
| p826_i0 | Job_involves_shifts | Job involves shift work |
| p21000_i0 | Ethnic_background | Ethnic background |
| p6139_i0Gas | p6139_i0Gas | A gas hob or gas cooker |
| p6139_i0Fire | p6139_i0Fire | A gas fire that you use regularly in winter time |
| p6139_i0Solid | p6139_i0Solid | An open solid fuel fire that you use regularly in winter |
| p670_i0 | Type_of_accomodation_lived_in | Type of accomodation lived in |
| p680_i0 | Own_or_rent | Own or rent accommodation lived in |
| p699_i0 | Length_time_current_adress | Length of time at current adress |
| p709_i0 | People_in_household | Number in household |
| p728_i0 | Vehicles_household | Number of vehicles in household |
| p738_i0 | Avg_tot_income | Average total household income before tax |
| p6146_i0 | p6146_i0Attendance_allowance | Do you receive attendance allowance |
| p6146_i0 | p6146_i0Blue_badge | Do you receive blue badge |
| p6146_i0 | p6146_i0Disability_living_allowance | Do you receive a disability living allowance |

**Table S12: Overview of verbal interview variables.**

| Field ID | Code | Variable |
| --- | --- | --- |
| p120_i0 | Birth_weight_known | Birth weight known |
| p20022_i0 | Birth_weight | Birth weight |
| p134_i0 | Self_reported_cancers | Number of self-reported cancers |
| p135_i0 | Self_reported_non_cancers | Number of self-reported non-cancers illnesses |
| p3140_i0 | Pregnant | Are you currently pregnant? |
| p137_i0 | Treatments_medications | Number of treatments/medications taken |
| p136_i0 | Self_reported_operations | Numbers of operations, self-reported |
| p3079_i0 | Pace_maker | Do you wear a pace-maker ? |

**Table S13: Overview of available prevalent ICD10-diagnosis codes, stored in field p41280_i0 and their assigned respective binary data-column, used as medical history variables.**

| ICD 10 codes | Assigned variable name |
| --- | --- |
| A00-A99, B00-B99 | Pres_infections_parasitic_diseases |
| C00-C99, D00-D49 | Pres_neoplasms |
| D50-D89 | Pres_blood_bloodorgan_immune_disease |
| E00-E09 | Pres_thyroid_disorder |
| E10-E14 | Pres_diabetes |
| E15-E65, E67-E99 | Pres_other_endocrine_metabolic_disease |
| E66 | Pres_obesity |
| F00-F99 | Pres_mental_disease |
| G00-G99 | Pres_nervous_system_disease |
| H00-H59 | Pres_eye_adnea_disease |
| H60-H99 | Pres_ear_mastoid_disease |
| I00-I09, I26-I28, I90-I99 | Pres_other_circulatory_diseases |
| I10-I15 | Pres_hypertensive_disease |
| I20-I25 | Pres_ischaemic_heart_disease |
| I30-I52 | Pres_other_heart_disease |
| J00-J99 | Pres_respiratory_disease |
| K00-K99 | Pres_digestive_disease |
| L00-L99 | Pres_skin_disease |
| M00-M99 | Pres_muscoskeletal_disease |
| N00-N99 | Pres_genitourinary_disease |
| O00-O99 | Pres_pregnancy_complications |

**Table S14: Participant number of all train- and hold-out sets for men and women.**

| **Dataset** | | **Participants (n)** | **Incident HF (n)** | **Incident HF (%)** |
| --- | --- | --- | --- | --- |
| Full set | Train set women | 212,799 | 2,330 | 1.09% |
|  | Hold-out set women | 53,507 | 564 | 1.05% |
|  | Train set men | 169,460 | 3,366 | 1.99% |
|  | Hold-out set men | 42,601 | 847 | 1.99% |
| Subsets | Train set women | 11,588 | 2,299 | 19.8% |
|  | Hold-out set women | 2,882 | 595 | 20.6% |
|  | Train set men | 16,870 | 3,375 | 20.0% |
|  | Hold-out set men | 4,195 | 838 | 20.0% |

**Legend:** The fourth column features percentages of people with incident HF in each dataset. Full data included the total sex-stratified UK Biobank population and subsets are the sex-specific datasets with a 1 to 4 HF case: non HF case population.

**Table S15: Missing data, stratified by sex, for each predictor variable.**

| **Code** | **Variable** | **Missing men** | **Missing women** |
| --- | --- | --- | --- |
| p1031_i0 | Frequency of friend/family visit | 3554 | 3913 |
| p1050_i0 | Time spend outdoors in summer | 11524 | 20334 |
| p1060_i0 | Time spend outdoors in winter | 11859 | 20129 |
| p1070_i0 | Time spent watching television (TV) | 1874 | 3141 |
| p1080_i0 | Time spent using computer | 3818 | 4945 |
| p1090_i0 | Time spent driving | 3750 | 5505 |
| p1100_i0 | Drive faster than motorway speed limit | 6044 | 9324 |
| p1110_i0 | Length of mobile phone use | 2973 | 4510 |
| p1120_i0 | Weekly usage of mobile phone in  last 3 months | 35972 | 44492 |
| p1130_i0 | Hands-free device/speakerphone  use with mobile phone in last three months | 35632 | 43107 |
| p1140_i0 | Difference in mobile phone use  compared to two years previously | 35665 | 43132 |
| p1150_i0 | Usual side of head for mobile phone use | 35035 | 42474 |
| p1160_i0 | Sleep duration | 1461 | 2431 |
| p1170_i0 | Getting up in morning | 2529 | 2831 |
| p1180_i0 | Morning/evening person (chronotype) | 24537 | 30948 |
| p1190_i0 | Nap during day | 792 | 998 |
| p120_i0 | Birth weight known | 422 | 374 |
| p1200_i0 | Sleeplessness/insomina | 630 | 792 |
| p1210_i0 | Snoring | 15738 | 19523 |
| p1220_i0 | Daytime dozing/sleeping (narcolepsy) | 1576 | 1976 |
| p1239_i0 | Current tobacco smoking | 532 | 713 |
| p1249_i0 | Past tobacco smoking | 17716 | 22339 |
| p1259_i0 | Smoking/smokers in household | 18678 | 23626 |
| p1269_i0 | Exposure to tobacco smoke at home | 21618 | 27117 |
| p1279_i0 | Exposure to tobacco smoke outside  home | 33986 | 42965 |
| p1289_i0 | Cooked vegetable intake | 3201 | 4019 |
| p1299_i0 | Salad/raw vegetable intake | 3256 | 4185 |
| p1309_i0 | Fresh fruit intake | 1376 | 1682 |
| p1319_i0 | Dried fruit intake | 2971 | 3611 |
| p1329_i0 | Oily fish intake | 1738 | 2125 |
| p1339_i0 | Non-oily fish intake | 1575 | 1946 |
| p134_i0 | Number of self-reported cancers | 422 | 374 |
| p1349_i0 | Processed meat intake | 938 | 1166 |
| p135_i0 | Number of self-reported non-  cancers illnesses | 422 | 374 |
| p1359_i0 | Poultry intake | 873 | 1084 |
| p136_i0 | Numbers of operations, self-reported | 422 | 374 |
| p1369_i0 | Beef intake | 1400 | 1752 |
| p137_i0 | Number of treatments/medications taken | 422 | 374 |
| p1379_i0 | Lamb/mutton intake | 1838 | 2424 |
| p1389_i0 | Pork intake | 1809 | 2255 |
| p1408_i0 | Cheese intake | 6034 | 7690 |
| p1418_i0 | Milk type used | 618 | 825 |
| p1428_i0 | Spread type | 2402 | 3127 |
| p1438_i0 | Bread intake | 4499 | 5750 |
| p1448_i0 | Bread type | 9549 | 12044 |
| p1458_i0 | Cereal intake | 1322 | 1627 |
| p1468_i0 | Cereal type | 38436 | 47701 |
| p1478_i0 | Salt added to food | 481 | 598 |
| p1488_i0 | Tea intake | 900 | 1178 |
| p1498_i0 | Coffee intake | 938 | 1222 |
| p1508_i0 | Coffee type | 49976 | 62405 |
| p1518_i0 | Hot drink temperature | 525 | 647 |
| p1528_i0 | Water intake | 2044 | 2547 |
| p1538_i0 | Major dietary changes in the last five  years | 989 | 1278 |
| p1548_i0 | Variation in diet | 3004 | 3851 |
| p1558_i0 | Alcohol intake frequency | 653 | 786 |
| p1568_i0 | Average weekly red wine intake | 66911 | 83665 |
| p1578_i0 | Average weekly champagne plus  white wine intake | 67013 | 83825 |
| p1588_i0 | Average weekly beer plus cider  intake | 66567 | 83312 |
| p1598_i0 | Average weekly spirits intake | 67118 | 84020 |
| p1608_i0 | Average weekly fortified wine intake | 66682 | 83426 |
| p1618_i0 | Alcohol usually taken with meals | 49082 | 61221 |
| p1628_i0 | Alcohol intake versus 10 years  previously | 19341 | 24125 |
| p1647_i0 | Country of birth (UK/elsewhere) | 758 | 960 |
| p1677_i0 | Breastfed as a baby | 49959 | 63161 |
| p1687_i0 | Comparative body size at age 10 | 4625 | 5864 |
| p1697_i0 | Comparative height size at age 10 | 4344 | 5542 |
| p1707_i0 | Handedness (chirality/laterality) | 502 | 631 |
| p1717_i0 | Skin colour | 3492 | 4357 |
| p1727_i0 | Ease of skin tanning | 7890 | 9957 |
| p1737_i0 | Childhood sunburn occasions | 53549 | 67225 |
| p1747_i0 | Hair colour (natural, before greying) | 896 | 1129 |
| p1757_i0 | Facial ageing | 18983 | 24138 |
| p1767_i0 | Adopted as a child | 1098 | 1282 |
| p1777_i0 | Part of a multiple birth | 3869 | 4851 |
| p1787_i0 | Maternal smoking around birth | 29283 | 37053 |
| p1797_i0 | Father still alive | 6244 | 7811 |
| p1807_i0 | Fathers age at death | 57200 | 71648 |
| p1835_i0 | Mother still alive | 4046 | 5108 |
| p1873_i0 | Number of full brothers | 3999 | 4975 |
| p1883_i0 | Number of full sisters | 3947 | 4944 |
| p1920_i0 | Mood swings | 6142 | 7525 |
| p1930_i0 | Miserableness | 4337 | 5566 |
| p1940_i0 | Irritatbility | 10356 | 13009 |
| p1950_i0 | Sensitivity/hurt feelings | 6900 | 8662 |
| p1960_i0 | Fed-up feelings | 5283 | 6666 |
| p1970_i0 | Nervous feelings | 6348 | 7889 |
| p1980_i0 | Worrier/anxious feelings | 6371 | 7833 |
| p1990_i0 | Tense/'highly strung' | 8495 | 10416 |
| p2000_i0 | Worry too long after embarassment | 9503 | 11814 |
| p20015_i0 | Sitting height | 2774 | 3087 |
| p20022_i0 | Birth weight | 110894 | 100805 |
| p2010_i0 | Suffer from 'nerves' | 8804 | 10860 |
| p20116_i0 | Smoking status | 1270 | 1437 |
| p20117_i0 | Alcohol drinker status | 747 | 798 |
| p20127_i0 | Neuroticism score | 39515 | 56084 |
| p20150_i0 | Forced expiratory volume in 1  -second (FEV1) Best measure | 59319 | 80454 |
| p20151_i0 | Forced vital capacity (FVC), best  score | 59319 | 80454 |
| p20153_i0 | Forced expiratory volume in 1-  second (FEV1) | 102505 | 112073 |
| p20154_i0 | Forced expiratory volume in 1-  second (FEV1), predicted  percentage | 102505 | 112073 |
| p20160_i0 | Ever smoked | 1243 | 1411 |
| p2020_i0 | Loneliness, isolation | 4152 | 5233 |
| p20256 | Forced expiratory volume in 1-  second (FEV1) Z-score | 56417 | 57937 |
| p20257 | Forced vital capacity (FVC), Z-score | 56417 | 57937 |
| p20258 | FEV1/FVC ratio Z-score | 56417 | 57937 |
| p2030_i0 | Guilty feelings | 6475 | 8195 |
| p2040_i0 | Risk taking | 8645 | 10857 |
| p2050_i0 | Frequency of depressed mood in last  2 weeks | 10222 | 12779 |
| p2060_i0 | Frequency of enthusiasm/disinterest in last 2  weeks | 8468 | 10386 |
| p2070_i0 | Frequency of tenseness/restlessness  in last 2 weeks | 8545 | 12313 |
| p2080_i0 | Frequency of tiredness/lethargy in  last 2 weeks | 6906 | 9422 |
| p2090_i0 | Seen doctor (GP) for nerves, anxiety, tension or depression | 1959 | 2759 |
| p2100_i0 | Seen a psychiatrist for nerves, anxiety, tension or depression | 1571 | 1883 |
| p21000_i0 | Ethnic background | 411 | 437 |
| p21001_i0 | Body mass index (BMI) | 1429 | 1378 |
| p21002_i0 | Weight | 1234 | 1285 |
| p21003_i0 | Age when attended assessment  centre | 0 | 0 |
| p21022 | Age at recruitment | 0 | 1 |
| p2110_i0 | Able to confide | 8823 | 8925 |
| p2129_i0 | Answered sexual history questions | 419 | 446 |
| p2139_i0 | Age first had sexual intercourse | 24466 | 34022 |
| p2149_i0 | Lifetime number of sexual partners | 40862 | 50344 |
| p2159_i0 | Ever had same-sex intercourse | 20047 | 29805 |
| p2178_i0 | Overall health rating | 1467 | 1711 |
| p2188_i0 | Long-standing illness, disability or  infirmity | 5111 | 7499 |
| p22033_i0 | Summed days activity | 30239 | 53318 |
| p22034_i0 | Summed minutes activity | 34236 | 60451 |
| p22037_i0 | MET minutes per week for walking | 34236 | 60451 |
| p22038_i0 | MET minutes per week for moderate  activity | 34236 | 60451 |
| p22039_i0 | MET minutes per week for vigorous  activity | 34236 | 60451 |
| p22040_i0 | Summed MET minutes per week for all  activity | 34236 | 60451 |
| p2207_i0 | Wears glasses or contact lenses | 765 | 761 |
| p2217_i0 | Age started wearing glasses or contact lenses | 36336 | 38281 |
| p2227_i0 | Other eye problems | 1134 | 1245 |
| p2237_i0 | Plays computer games | 765 | 824 |
| p2247_i0 | Hearing difficulty/problems | 9076 | 14211 |
| p2257_i0 | Hearing difficulty/problems with  background noise | 4796 | 6334 |
| p2267_i0 | Use of sun/uv protection | 2643 | 2886 |
| p2277_i0 | Frequency of solarium/sunlamp use | 4378 | 5452 |
| p2296_i0 | Falls in the last year | 1223 | 1250 |
| p2306_i0 | Weight change compared to 1 year ago | 4610 | 5117 |
| p23098_i0 | Weight | 3964 | 4746 |
| p23099_i0 | Body fat percentage | 4233 | 4748 |
| p23100_i0 | Whole body fat mass | 4701 | 4822 |
| p23101_i0 | Whole body fat-free mass | 4003 | 4749 |
| p23102_i0 | Whole body water mass | 3967 | 4749 |
| p23104_i0 | Body mass index (BMI) | 3969 | 4749 |
| p23105_i0 | Basal metabolic rate | 3973 | 4755 |
| p23106_i0 | Impedance of whole body | 3974 | 4792 |
| p23107_i0 | Impedance of leg (right) | 3975 | 4764 |
| p23108_i0 | Impedance of leg (left) | 3978 | 4767 |
| p23109_i0 | Impedance of arm (right) | 3982 | 4792 |
| p23110_i0 | Impedance of arm (left) | 3980 | 4773 |
| p23111_i0 | Leg fat percentage (right) | 3979 | 4769 |
| p23112_i0 | Leg fat mass (right) | 3985 | 4772 |
| p23113_i0 | Let fat-free mass (right) | 3991 | 4777 |
| p23114_i0 | Leg predicted mass (right) | 3991 | 4778 |
| p23115_i0 | Leg fat percentage (left) | 3996 | 4783 |
| p23116_i0 | Leg fat mass (left) | 3999 | 4785 |
| p23117_i0 | Leg fat-free mass (left) | 4007 | 4796 |
| p23118_i0 | Leg predicted mass (left) | 4010 | 4799 |
| p23119_i0 | Arm fat percentage (right) | 4019 | 4802 |
| p23120_i0 | Arm fat mass (right) | 4022 | 4831 |
| p23121_i0 | Arm fat-free mass (right) | 4031 | 4828 |
| p23122_i0 | Arm predicted mass (right) | 4033 | 4834 |
| p23123_i0 | Arm fat percentage (left) | 4045 | 4844 |
| p23124_i0 | Arm fat mass (left) | 4061 | 4869 |
| p23125_i0 | Arm fat-free mass (left) | 4073 | 4873 |
| p23126_i0 | Arm predicted mass (left) | 4083 | 4882 |
| p23127_i0 | Trunk fat percentage | 4103 | 4903 |
| p23128_i0 | Trunk fat mass | 4115 | 4915 |
| p23129_i0 | Trunk fat-free mass | 4153 | 4957 |
| p23130_i0 | Trunk predicted mass | 4174 | 4981 |
| p2316_i0 | Wheeze or whistling in the chest in  last year | 5572 | 5470 |
| p2335_i0 | Chest pain or discomfort | 2988 | 3406 |
| p2345_i0 | Ever had bowel cancer screening | 5440 | 3561 |
| p24003 | Nitrogen dioxide air pollution; 2010 | 3194 | 3840 |
| p24004 | Nitrogen oxides air pollution; 2010 | 3194 | 3840 |
| p24005 | Particulate matter air pollution  (pm10); 2010 | 17033 | 22152 |
| p24006 | Particulate matter air pollution  (pm2.5); 2010 | 17033 | 22152 |
| p24007 | Particulate matter air pollution  (pm10) absorbance; 2010 | 17033 | 22152 |
| p24008 | Particulate matter air pollution  (2.5-10 um); 2010 | 17033 | 22152 |
| p24009 | Traffic intensity on nearest road | 3194 | 3840 |
| p24010 | Inverse distance to the nearest road | 3194 | 3840 |
| p24011 | Traffic intensity on the nearest  major road | 3194 | 3840 |
| p24012 | Inverse distance to the nearest  major road | 3194 | 3840 |
| p24013 | Total traffic load on major roads | 3194 | 3840 |
| p24014 | Close to major road | 3194 | 3840 |
| p24015 | Sum of road length of major roads  within 100m | 3194 | 3840 |
| p24016 | Nitrogen dioxide air pollution; 2005 | 3194 | 3840 |
| p24017 | Nitrogen dioxide air pollution; 2006 | 3194 | 3840 |
| p24018 | Nitrogen dioxide air pollution; 2007 | 3194 | 3840 |
| p24019 | Particulate matter air pollution (pm10); 2007 | 3631 | 4442 |
| p24020 | Average daytime sound level of  noise pollution | 3194 | 3840 |
| p24021 | Average evening sound level of  noise pollution | 3194 | 3840 |
| p24022 | Average night-time sound level of  noise pollution | 3194 | 3840 |
| p24023 | Average 16-hour sound level of  noise pollution | 3194 | 3840 |
| p24024 | Average 24-hour sound level of  noise pollution | 3194 | 3840 |
| p2443_i0 | Diabetes diagnosed by doctor | 1249 | 1133 |
| p24500_i0 | Greenspace percentage, buffer 1000 m | 25868 | 32737 |
| p24501_i0 | Domestic garden percentage, buffer 1000 m | 25868 | 32737 |
| p24502_i0 | Water percentage, buffer 1000 m | 25868 | 32737 |
| p24503_i0 | Greenspace percentage, buffer 300 m | 25868 | 32737 |
| p24504_i0 | Domestic garden percentage, buffer 300 m | 25868 | 32737 |
| p24505_i0 | Water percentage, buffer 300 m | 25868 | 32737 |
| p24506_i0 | Natural environment percentage, buffer 1000 m | 2385 | 2387 |
| p24507_i0 | Natural environment percentage, buffer 300 m | 2385 | 2387 |
| p24508_i0 | Distance (Euclidian) to coast | 2385 | 2387 |
| p2453_i0 | Cancer diagnosed by doctor | 1014 | 1565 |
| p2463_i0 | Fractured/broken bones in last 5 years | 1883 | 1709 |
| p2473_i0 | Other serious medical condition/disability diagnosed by doctor | 3635 | 6095 |
| p2492_i0 | Taking other prescription medications | 1454 | 1395 |
| p26410 | Index of Multiple Deprivation (England) | 29246 | 37056 |
| p26411 | Income score (England) | 29246 | 37056 |
| p26412 | Employment score (England) | 29246 | 37056 |
| p26413 | Health score (England) | 29246 | 37056 |
| p26414 | Education score (England) | 29246 | 37056 |
| p26415 | Housing score (England) | 29246 | 37056 |
| p26416 | Crime score (England) | 29246 | 37056 |
| p26417 | Living environment score (England) | 29246 | 37056 |
| p2674_i0 | Ever had breast cancer screening/mammogram | x | 951 |
| p2694_i0 | Ever had cervical smear test | x | 1183 |
| p2714_i0 | Age when periods started (menarche) | x | 8616 |
| p2724_i0 | Had menopause | x | 964 |
| p2734_i0 | Number of live births | x | 799 |
| p2774_i0 | Ever had stillbirth, spontaneous miscarriage or termination | x | 4916 |
| p2784_i0 | Ever taken oral-contraceptive pill | x | 1361 |
| p2814_i0 | Ever used hormone replacement therapy (HRT) | x | 1513 |
| p2834_i0 | Bilateral oophorectomy (both ovaries removed) | x | 790 |
| p2844_i0 | Had other major operations | x | 2945 |
| p30000_i0 | White blood cell (leukocyte) count | 9030 | 14045 |
| p30010_i0 | Red blood cell (Erythrocyte) count | 9027 | 14043 |
| p30020_i0 | Haemoglobin concentration | 9028 | 14042 |
| p30030_i0 | Haematocrit concentration | 9027 | 14043 |
| p30040_i0 | Mean corpuscular volume | 9027 | 14044 |
| p30050_i0 | Mean corpuscular haemoglobin count | 9028 | 14045 |
| p30060_i0 | Mean corpuscular haemoglobin concentration | 9029 | 14047 |
| p30070_i0 | Red blood cell (Erythrocyte) distribution width | 9027 | 14044 |
| p30080_i0 | Platelet count | 9028 | 14045 |
| p30090_i0 | Platelet crit | 9030 | 14047 |
| p30100_i0 | Mean platelet (thrombocyte) volume | 9030 | 14048 |
| p30110_i0 | Platelet distribution width | 9030 | 14048 |
| p30120_i0 | Lymphocyte count | 9414 | 14500 |
| p30130_i0 | Monocyte count | 9414 | 14500 |
| p30140_i0 | Neutrophil count | 9414 | 14500 |
| p30150_i0 | Eosinophil count | 9414 | 14500 |
| p30160_i0 | Basophil count | 9414 | 14500 |
| p30170_i0 | Nucleated red blood cell count | 9417 | 14507 |
| p30180_i0 | Lymphocyte percentage | 9411 | 14497 |
| p30190_i0 | Monocyte percentage | 9411 | 14497 |
| p30200_i0 | Neutrophil percentage | 9411 | 14497 |
| p30210_i0 | Eosinophil percentage | 9411 | 14497 |
| p30220_i0 | Basophil percentage | 9411 | 14497 |
| p30230_i0 | Nucleated red blood cell percentage | 9419 | 14509 |
| p30240_i0 | Reticulocyte percentage | 12467 | 18556 |
| p30250_i0 | Reticulocyte count | 12466 | 18557 |
| p30260_i0 | Mean reticulocyte volume | 12467 | 18557 |
| p30270_i0 | Mean sphered cell volume | 12466 | 18556 |
| p30280_i0 | Immature reticulocyte fraction | 12467 | 18557 |
| p30290_i0 | High light scatter reticulocyte percentage | 12466 | 18556 |
| p30300_i0 | High light scatter reticulocyte count | 12466 | 18557 |
| p30510_i0 | Creatinine (enzymatic) in urine | 6953 | 10167 |
| p30520_i0 | Potassium in urine | 7396 | 10706 |
| p30530_i0 | Sodium in urine | 7156 | 10930 |
| p30600_i0 | Albumin | 28039 | 40770 |
| p3061_i0_a0 | Acceptability of each blow result | 20623 | 25843 |
| p30610_i0 | Alkaline phospate | 12983 | 18159 |
| p3062_i0_a0 | Forced vital capacity (FVC) | 20623 | 25843 |
| p30620_i0 | Alanine aminotransferase | 13138 | 18187 |
| p3063_i0_a0 | Forced expiratory volume in 1-second (FEV1) | 20623 | 25843 |
| p30630_i0 | Apoliprotein A | 28430 | 42888 |
| p3064_i0_a0 | Peak expiratory flow (PEF) | 20623 | 25843 |
| p30640_i0 | Apoliprotein B | 14343 | 19038 |
| p30650_i0 | Aspartate aminotransferase | 13786 | 19071 |
| p30660_i0 | Direct birirubin | 27089 | 72985 |
| p30670_i0 | Urea | 13123 | 18339 |
| p30680_i0 | Calcium | 28098 | 40841 |
| p30690_i0 | Cholesterol | 12979 | 18182 |
| p30700_i0 | Creatinine | 13094 | 18291 |
| p30710_i0 | C-reactive protein | 13505 | 18609 |
| p30720_i0 | Cystatin C | 13005 | 18186 |
| p30730_i0 | Gamma glutamyltransferase | 13110 | 18285 |
| p30740_i0 | Glucose | 28273 | 41020 |
| p30750_i0 | Glycated haemoglobin (HbA1c) | 14313 | 19846 |
| p30760_i0 | HDL cholesterol | 28148 | 40847 |
| p30770_i0 | IGF-1 | 14032 | 19560 |
| p30780_i0 | LDL direct | 13408 | 18585 |
| p3079_i0 | Pace-maker | 1253 | 1478 |
| p30790_i0 | Lipoprotein A | 52789 | 66913 |
| p30810_i0 | Phosphate | 28393 | 41203 |
| p30830_i0 | SHBG | 29480 | 43388 |
| p30840_i0 | Total bilirubin | 13853 | 19235 |
| p30850_i0 | Testosterone | 14899 | 58894 |
| p30860_i0 | Total protein | 28270 | 40986 |
| p30870_i0 | Triglycerides | 13193 | 18315 |
| p3088_i0 | Contra-indications for spirometry | 1258 | 1481 |
| p30880_i0 | Urate | 13201 | 18487 |
| p3089_i0 | Caffeine drink within last hour | 17790 | 20014 |
| p30890_i0 | Vitamin D | 19498 | 32459 |
| p3090_i0 | Used an inhaler for chest within last hour | 17790 | 20014 |
| p3140_i0 | Pregnant | x | 804 |
| p3143_i0 | Ankle spacing width | 94187 | 118094 |
| p3144_i0 | Heel Broadband ultrasound attenuation, direct entry | 94196 | 118099 |
| p3146_i0 | Speed of sound through heel | 94187 | 118094 |
| p3147_i0 | Heel quantitative ultrasound index (QUI), direct entry | 94187 | 118094 |
| p3148_i0 | Heel bone mineral density (BMD) | 94296 | 118113 |
| p3393_i0 | Hearing aid user | 74490 | 113405 |
| p3526_i0 | Mother's age at death | 91271 | 113308 |
| p4079_i0_a0 | Diastolic blood pressure, automated reading | 14304 | 18320 |
| p4080_i0_a0 | Systolic blood pressure, automated reading | 14311 | 18325 |
| p46_i0 | Hand grip strength (left) | 1359 | 1781 |
| p47_i0 | Hand grip strength (right) | 1375 | 1732 |
| p48_i0 | Waist circumference | 957 | 1029 |
| p49_i0 | Hip circumference | 990 | 1048 |
| p50_i0 | Standing height | 1218 | 1091 |
| p51_i0 | Seated hight | 2774 | 3087 |
| p6138_i0 | Qualifications | 4311 | 5149 |
| p6139_i0 | p6139_i0cooker | 0 | 0 |
| p6142_i0 | Current employment status | 2464 | 3018 |
| p6145_i0 | Illness, injury, bereavement, stress in last 2 years | 2003 | 2441 |
| p6146_i0 | Attendance/disability/mobility allowance | 419 | 447 |
| p6149_i0 | Mouth/teeth dental problems | 2003 | 2445 |
| p6150_i0 | Vascular/heart problems diagnosed by doctor | 424 | 453 |
| p6152_i0 | Blood clot, DVT, bronchitis, emphysema, asthma, rhinitis, eczema, allergy diagnosed by doctor | 425 | 453 |
| p6153_i0 | Medication for cholesterol, blood pressure, diabetes, or take exogenous hormones | x | 2460 |
| p6154_i0 | Medication for pain relief, constipation, heartburn | 2008 | 2452 |
| p6155_i0 | Vitamin and mineral supplements | 2008 | 2452 |
| p6159_i0 | Pain type(s) experienced in last month | 421 | 449 |
| p6160_i0 | Leisure/social activities | 403 | 432 |
| p6162 | Type of transport used (excluding work) | 2646 | 3323 |
| p6164_i0 | Types of physical activity in last 4 weeks | 2519 | 3225 |
| p6177_i0 | Medication for cholesterol, blood pressure, or diabetes | 2014 | x |
| p6179_i0 | Minerals and other dietary supplements | 2008 | 2453 |
| p670_i0 | Type of accomodation lived in | 642 | 654 |
| p680_i0 | Own or rent accommodation lived in | 2399 | 3035 |
| p699_i0 | Length of time at current adress | 1335 | 1167 |
| p709_i0 | Number in household | 1916 | 2229 |
| p728_i0 | Number of vehicles in household | 2067 | 2155 |
| p738_i0 | Average total household income before tax | 24388 | 48181 |
| p74_i0 | Fasting time | 607 | 536 |
| p757_i0 | Time employed in main current job | 80175 | 119373 |
| p767_i0 | Length of working week for main job | 81404 | 121188 |
| p777_i0 | Frequency of travelling from home to job workplace | 82241 | 121604 |
| p78_i0 | Heel bone mineral density (BMD) T-score, automated | 94187 | 118094 |
| p806_i0 | Job involves mainly walking or standing | 79874 | 118959 |
| p816_i0 | Job involves heavy manual or physical work | 79805 | 118982 |
| p826_i0 | Job involves shift work | 79998 | 119151 |
| p845_i0 | Age completed full-time education | 77467 | 89313 |
| p864_i0 | Number of days/week walked 10+ minutes | 3146 | 4663 |
| p874_i0 | Duration of walks | 27219 | 45046 |
| p884_i0 | Number of days/week of moderate physical activity 10+ minutes | 8827 | 16402 |
| p894_i0 | Duration of moderate activity | 50911 | 75149 |
| p904_i0 | Number of days/week of vigorous physical activity 10+ minutes | 9727 | 15719 |
| p914_i0 | Duration of vigurous activity | 87373 | 131658 |
| p924_i0 | Usual walking pace | 1311 | 1570 |
| p943_i0 | Frequency of stair climbing in last 4 weeks | 3822 | 4961 |
| p971_i0 | Frequency of walking for pleasure in last 4 weeks | 69158 | 74704 |
| p981_i0 | Duration walking for pleasure | 69201 | 74791 |

**Legend:** An x showcases that a variable was unavailable for that sex.

**Hyper parameter tuning in the ML models.**

The details regarding the range of chosen values to tune, as well as parameter definitions, are displayed here.

The RSF model has the following tuning parameters:

- mtry: Integer which defines the number of randomly sampled variables used as candidates to facilitate a split in a single decision tree. For each split in a tree, a new subsample is defined.
- min_node_sizes: Stopping criterion which defines how many participants can remain in a node before it becomes a leaf node i.e. is not split again. If a node is split in two daughter nodes, of which one is smaller than min_node_sizes, then this node is not split.
- Ntree: Number of trees to grow to create the ensemble of decision trees.

The XGBoost algorithm features the following tuning parameters:

- eta: Step-size used as learning rate between iterations. Used to prevent overfitting.
- max_depth: Maximum allowed depth of a decision tree.
- gamma: Minimum loss reduction required to facilitate an additional partition on a leaf node in the decision tree.
- Subsample: Subsample ratio of randomly selected rows of training data used to grow a decision tree.
- Colsample_by_tree: Subsample ratio of randomly selected features to use to grow a tree. This selection is preceded by the subsample process.
- Min_child_weight: minimum number of instance weight needed in a daughter node to become a leaf node. Shows similarities to min_node_sizes in the RSF algorithm.
- Nrounds: Similar to Ntree, defines the number of iterations used to grow the XGBoost model.

**Table S16: Explored hyperparameter values for Random Survival Forest and XGBoost.**

| Model | Hyperparameter | Tried values | Optimum values (men) | Optimum values (women) |
| --- | --- | --- | --- | --- |
| Random Survival Forest | Mtry | 1,2,3,6,10,21,42,84,168,336 | 3 | 21 |
|  | min_node_sizes | 3,10,20,50 | 3 | 50 |
|  | n_tree | 1000 | 1000 | 1000 |
| XGBoost | Eta | (2,6,10)/nrounds | 0.02 | 0.02 |
|  | max_depth | 4, 6, 8 | 4 | 4 |
|  | Gamma | 0, 0.5, 1 | 0 | 0 |
|  | Subsample | 0.5, 0.75, 1 | 0.5 | 0.75 |
|  | colsample_by_tree | 0.4, 0.6, 0.8, 1 | 1 | 0.4 |
|  | min_child_weight | 3/event_rate | 0.1513673 | 0.1497 |
|  | Nrounds | 500 | 500 | 500 |

**Table S17: Baseline characteristics of the subset population (N = 35,535), used for variable importance assessment.**

| **Characteristic** | **Men**, N = 21,065 | **Women**, N = 14,470 |
| --- | --- | --- |
| **Age (years)** | 59, (51, 64) | 59, (51, 64) |
| **Ethnic background** |  |  |
| Any other white background | 539 (2.6%) | 503 (3.5%) |
| British | 18,690 (89%) | 12,747 (88%) |
| Indian | 269 (1.3%) | 152 (1.1%) |
| Irish | 590 (2.8%) | 387 (2.7%) |
| Other | 977 (4.6%) | 681 (4.5%) |
| **Waist circumference (cm)** | 96, (90, 104) | 84, (76, 94) |
| **BMI (kg/m2)** | 27.5, (25.1, 30.3) | 26.6, (23.7, 30.4) |
| **Best FEV1 measure (L)** | 3.26 (2.73, 3.77) | 2.32 (1.94, 2.69) |
| **Systolic blood pressure (mmHg)** | 142, (131, 155) | 137, (124, 152) |
| **Diastolic blood pressure (mmHg)** | 84, (77, 91) | 81, (74, 88) |
| **Smoking status** |  |  |
| Current | 2,754 (13%) | 1,511 (10%) |
| Never | 10,060 (48%) | 8,262 (57%) |
| Previous | 8,251 (39%) | 4,697 (32%) |
| **Albumin (g/L)** | 45.39, (43.67, 47.13) | 44.78, (43.08, 46.49) |
| **Cystatin-C (mg/L)** | 0.92, (0.85, 1.03) | 0.87, (0.79, 0.98) |
| **CRP (mg/L)** | 1.37, (0.70, 2.76) | 1.55, (0.70, 3.38) |
| **Creatinine (mmol/L)** | 80, (72, 89) | 63, (57, 71) |
| **Cholesterol (mmol/L)** | 5.48, (4.74, 6.24) | 5.81, (5.06, 6.59) |
| **HDL cholesterol (mmol/L)** | 1.24, (1.06, 1.46) | 1.54, (1.30, 1.82) |
| **Haemoglobin concentration (g/dL)** | 15.00, (14.36, 15.68) | 13.50, (12.90, 14.12) |
| **HbA1c (mmol/mol)** | 35.4, (32.9, 38.3) | 35.5, (33.1, 38.2) |
| **RDW (%)** | 13.35, (12.92, 13.86) | 13.40, (12.91, 14.00) |
| **Presence other heart disease** | 714 (3.4%) | 389 (2.7%) |
| **Presence of diabetes** | 707 (3.4%) | 324 (2.2%) |
| **Blood pressure medication use** | 5,241 (25%) | 2,894 (20%) |

**Legend:** Continuous variables are presented as median (P25,P75). Categorical variables are presented as number (%). Abbreviations: BMI, body mass index; CRP, C-reactive protein; RDW, Red blood cell distribution width; FEV1, Forced expiratory volume in 1 second.

**Table S18: UK Biobank baseline characteristics, stratified by incident HF event status.**

| **Characteristic** | **No incident HF**, N = 471,260 | **Incident HF**, N = 7,107 |
| --- | --- | --- |
| **Age (years)** | 57, (50, 63) | 63, (59, 67) |
| **Sex** |  |  |
| Men | 207,848 (44%) | 4,213 (59%) |
| Women | 263,412 (56%) | 2,894 (41%) |
| **Ethnic background** |  |  |
| Any other white background | 15,672 (3.3%) | 155 (2.2%) |
| British | 415,627 (88%) | 6,336 (89%) |
| Indian | 5,436 (1.2%) | 103 (1.4%) |
| Irish | 12,375 (2.6%) | 194 (2.7%) |
| Other | 22,150 (4.7%) | 319 (4.5%) |
| **Waist circumference (cm)** | 89, (80, 98) | 98, (88, 108) |
| **BMI (kg/m2)** | 26.6, (24.1, 29.7) | 28.7, (25.6, 32.8) |
| **Best FEV1 measure (l)** | 2.74, (2.26, 3.33) | 2.39, (1.87, 3.01) |
| **Systolic blood pressure (mmHg)** | 138, (126, 152) | 146, (134, 161) |
| **Diastolic blood pressure (mmHg)** | 82, (75, 89) | 84, (76, 91) |
| **Smoking status** |  |  |
| Current | 49,133 (10%) | 1,152 (16%) |
| Never | 262,876 (56%) | 3,007 (42%) |
| Previous | 159,251 (34%) | 2,948 (42%) |
| **Albumin (g/L)** | 45.22, (43.51, 46.95) | 44.41, (42.59, 46.17) |
| **Cystatin-C (mg/L)** | 0.88, (0.80, 0.98) | 0.99, (0.89, 1.12) |
| **CRP (mg/L)** | 1.31, (0.65, 2.73) | 2.23, (1.05, 4.68) |
| **Creatinine (mmol/L)** | 70, (61, 80) | 75, (64, 86) |
| **Cholesterol (mmol/L)** | 5.70, (4.98, 6.48) | 5.41, (4.56, 6.28) |
| **HDL cholesterol (mmol/L)** | 1.41, (1.18, 1.70) | 1.29, (1.08, 1.57) |
| **Haemoglobin concentration (g/dL)** | 14.12, (13.30, 15.00) | 14.30, (13.39, 15.22) |
| **HbA1c (mmol/mol)** | 35.1, (32.7, 37.7) | 37.1, (34.2, 40.7) |
| **RDW (%)** | 13.33, (12.90, 13.85) | 13.60, (13.10, 14.30) |
| **Presence other heart disease** | 6,827 (1.5%) | 658 (9.3%) |
| **Presence of diabetes** | 7,242 (1.5%) | 549 (7.7%) |
| **Blood pressure medication use** | 85,433 (18%) | 3,083 (43%) |

**Legend:** Continuous variables are presented as median (P25,P75). Categorical variables are presented as number (%). Abbreviations: BMI, body mass index; CRP, C-reactive protein; RDW, Red blood cell distribution width; FEV1, Forced expiratory volume in 1 second.

**Table S19: Baseline characteristics of men, stratified by incident HF event status.**

| **Characteristic** | **No incident HF**, N = 207,848 | **Incident HF**, N = 4,213 |
| --- | --- | --- |
| **Age (years)** | 57, (49, 63) | 63, (59, 67) |
| **Ethnic background** |  |  |
| Any other white background | 5,865 (2.8%) | 80 (1.9%) |
| British | 183,734 (88%) | 3,786 (90%) |
| Indian | 2,643 (1.3%) | 61 (1.4%) |
| Irish | 5,766 (2.8%) | 102 (2.4%) |
| Other | 9,840 (4.7%) | 184 (4.4%) |
| **Waist circumference (cm)** | 96, (89, 103) | 101, (93, 110) |
| **BMI (kg/m2)** | 27.2, (24.9, 29.9) | 28.7, (25.9, 32.3) |
| **Best FEV1 measure** | 3.35, (2.85, 3.84) | 2.81, (2.25, 3.34) |
| **Systolic blood pressure (mmHg)** | 141, (130, 154) | 148, (135, 161) |
| **Diastolic blood pressure (mmHg)** | 84, (77, 91) | 85, (77, 92) |
| **Smoking status** |  |  |
| Current | 25,856 (12%) | 739 (18%) |
| Never | 104,822 (51%) | 1,583 (37%) |
| Previous | 77,170 (37%) | 1,891 (45%) |
| **Albumin (g/L)** | 45.57, (43.87, 47.27) | 44.59, (42.82, 46.34) |
| **Cystatin-C (mg/L)** | 0.91, (0.84, 1.00) | 1.00, (0.90, 1.13) |
| **CRP (mg/L)** | 1.26, (0.65, 2.50) | 2.05, (1.00, 4.19) |
| **Creatinine (mmol/L)** | 80, (72, 88) | 81, (72, 91) |
| **Cholesterol (mmol/L)** | 5.54, (4.82, 6.29) | 5.20, (4.40, 6.03) |
| **HDL cholesterol (mmol/L)** | 1.25, (1.07, 1.47) | 1.19, (1.01, 1.42) |
| **Haemoglobin concentration (g/dL)** | 15.03, (14.40, 15.68) | 14.90, (14.03, 15.66) |
| **HbA1c (mmol/mol)** | 35.1, (32.7, 37.8) | 37.1, (34.1, 40.9) |
| **RDW (%)** | 13.30, (12.90, 13.80) | 13.58, (13.10, 14.20) |
| **Presence other heart disease** | 3,921 (1.9%) | 404 (9.6%) |
| **Presence of diabetes** | 4,070 (2.0%) | 367 (8.7%) |
| **Blood pressure medication use** | 42,615 (21%) | 1,867 (44%) |

**Legend:** Continuous variables are presented as median (P25,P75). Categorical variables are presented as number (%). Abbreviations: BMI, body mass index; CRP, C-reactive protein; RDW, Red blood cell distribution width; FEV1, Forced expiratory volume in 1 second.

**Table S20: Baseline characteristics of women, stratified by incident HF event status.**

**Legend:** Continuous variables are presented as median (P25,P75). Categorical variables are presented as number (%). Abbreviations: BMI, body mass index; CRP, C-reactive protein; RDW, Red blood cell distribution width; FEV1, Forced expiratory volume in 1 second.

| **Characteristic** | **No incident HF**, N = 263,412 | **Incident HF**, N = 2,894 |
| --- | --- | --- |
| **Age (years)** | 57, (50, 63) | 64, (59, 67) |
| **Ethnic background** |  |  |
| Any other white background | 9,807 (3.7%) | 75 (2.6%) |
| British | 231,893 (88%) | 2,550 (89%) |
| Indian | 2,793 (1.1%) | 42 (1.1%) |
| Irish | 6,609 (2.5%) | 92 (3.2%) |
| Other | 12,310 (4.7%) | 135 (4.6%) |
| **Waist circumference (cm)** | 83, (75, 92) | 90, (81, 103) |
| **BMI (kg/m2)** | 26.1, (23.4, 29.6) | 28.8, (25.1, 33.7) |
| **Best FEV1 measure** | 2.41, (2.05, 2.77) | 1.97, (1.59, 2.31) |
| **Systolic blood pressure (mmHg)** | 135, (122, 150) | 145, (132, 160) |
| **Diastolic blood pressure (mmHg)** | 80, (73, 88) | 82, (75, 90) |
| **Smoking status** |  |  |
| Current | 23,276 (8.8%) | 413 (14%) |
| Never | 158,055 (60%) | 1,424 (49%) |
| Previous | 82,080 (31%) | 1,057 (37%) |
| **Albumin (g/L)** | 44.95, (43.26, 46.68) | 44.17, (42.29, 45.93) |
| **Cystatin-C (mg/L)** | 0.85, (0.77, 0.95) | 0.97, (0.86, 1.11) |
| **CRP (mg/L)** | 1.36, (0.64, 2.94) | 2.52, (1.14, 5.48) |
| **Creatinine (mmol/L)** | 63, (57, 70) | 65, (57, 74) |
| **Cholesterol (mmol/L)** | 5.84, (5.11, 6.62) | 5.73, (4.90, 6.56) |
| **HDL cholesterol (mmol/L)** | 1.56, (1.32, 1.83) | 1.47, (1.22, 1.75) |
| **Haemoglobin concentration (g/dL)** | 13.50, (12.90, 14.10) | 13.56, (12.88, 14.23) |
| **HbA1c (mmol/mol)** | 35.1, (32.7, 37.6) | 37.0, (34.3, 40.5) |
| **RDW (%)** | 13.36, (12.90, 13.90) | 13.69, (13.10, 14.40) |
| **Presence other heart disease** | 2,906 (1.1%) | 254 (8.8%) |
| **Presence of diabetes** | 3,169 (1.2%) | 182 (6.3%) |
| **Blood pressure medication** | 42,818 (16%) | 1,216 (42%) |

**Table S21: Performance metrices of UK Biobank data in PCP-HF score.**

| **C-indices** | **Full data** | **Subdata** |
| --- | --- | --- |
| **Men** | 0.73 | 0.74 |
| **Women** | 0.77 | 0.77 |

**Legend:** The PCP-score was calculated on the full train sets. Scores were derived using the formulas as defined in the PCP-HF literature. Performances are defined as C-indices.

**Table S22: P-values and importance scores for the 15 most important predictor variables in subset men.**

| **Scores** | **P-values** | | | **Permutation** | **Gain** |
| --- | --- | --- | --- | --- | --- |
| **Rank** | **Ordinary Cox** | **Randomized LASSO** | **LASSO** | **RSF** | **XGBoost** |
| 1 | 2.27e-23 | 2.97e-91 | 3.86e-89 | 0.0106 | 0.12 |
| 2 | 1.81e-16 | 4.94e-36 | 1.21e-34 | 0.0085 | 0.070 |
| 3 | 5.73e-07 | 6.78e-33 | 5.67e-32 | 0.0084 | 0.064 |
| 4 | 8.51e-07 | 1.14e-14 | 1.48e-12 | 0.0081 | 0.060 |
| 5 | 1.39e-05 | 1.98e-13 | 1.26e-10 | 0.0078 | 0.030 |
| 6 | 1.84e-05 | 1.07e-12 | 3.61e-10 | 0.0070 | 0.025 |
| 7 | 2.61e-05 | 3.34e-11 | 5.42e-10 | 0.0064 | 0.019 |
| 8 | 4.03e-05 | 1.3e-09 | 6.25e-09 | 0.0064 | 0.018 |
| 9 | 7.89e-05 | 1.36e-09 | 1.49e-08 | 0.0062 | 0.015 |
| 10 | 8.12e-05 | 5.07e-09 | 3.19e-08 | 0.0059 | 0.014 |
| 11 | 8.42e-05 | 7.35e-07 | 3.21e-06 | 0.0058 | 0.014 |
| 12 | 0.000105 | 4.27e-05 | 1.11e-05 | 0.0057 | 0.014 |
| 13 | 0.000161 | 7.32e-05 | 2.32e-05 | 0.0054 | 0.013 |
| 14 | 0.000216 | 0.001769 | 3.98e-05 | 0.0052 | 0.013 |
| 15 | 0.000751 | 0.010513 | 7.16e-05 | 0.0052 | 0.013 |

**Legend:** Rank signifies the row to which the row-values correspond to in Table 3.

**Table S23: P-values and importance scores for the 15 most important predictor variables in subset women.**

|  | **P-values** | | | **Permutation** | **Gain** |
| --- | --- | --- | --- | --- | --- |
| **Rank** | **Ordinary Cox** | **Randomized LASSO** | **LASSO** | **RSF** | **XGBoost** |
| 1 | 1.67e-19 | 3.9e-72 | 9.8e-74 | 0.048 | 0.11 |
| 2 | 3.26e-12 | 5.11e-35 | 2.65e-33 | 0.046 | 0.086 |
| 3 | 3.26e-09 | 4.42e-26 | 7.38e-27 | 0.041 | 0.083 |
| 4 | 3.8e-06 | 1.14e-15 | 2.35e-12 | 0.039 | 0.072 |
| 5 | 9.99e-05 | 3.07e-11 | 1.5e-10 | 0.032 | 0.031 |
| 6 | 0.00057 | 3.6e-11 | 1.38e-09 | 0.032 | 0.022 |
| 7 | 0.00071 | 5.25e-08 | 1.59e-05 | 0.027 | 0.021 |
| 8 | 0.001592 | 2.59e-07 | 1.61e-05 | 0.025 | 0.017 |
| 9 | 0.001877 | 1.93e-05 | 4.06e-05 | 0.025 | 0.016 |
| 10 | 0.00285 | 4.39e-05 | 0.000143 | 0.024 | 0.013 |
| 11 | 0.004364 | 0.000524 | 0.000167 | 0.024 | 0.012 |
| 12 | 0.004395 | 0.000987 | 0.001465 | 0.023 | 0.012 |
| 13 | 0.004717 | 0.001857 | 0.003856 | 0.023 | 0.011 |
| 14 | 0.006058 | 0.003322 | 0.00876 | 0.022 | 0.010 |
| 15 | 0.006361 | 0.007018 | 0.012043 | 0.019 | 0.0092 |

**Legend:** Rank signifies the row to which the row-values correspond to in Table 4.

**Table S24: Remaining numbers of predictors after LASSO stability selection in the full datasets and subsets.**

| Dataset | **Sex** | **Weakness (**$\boldsymbol{\alpha)}$ | **# variables** |
| --- | --- | --- | --- |
| Full train data | Men | 0.8 | 16 |
|  |  | 1 | 18 |
|  | Women | 0.8 | 15 |
|  |  | 1 | 17 |
| Subset train data | Men | 0.8 | 18 |
|  |  | 1 | 15 |
|  | Women | 0.8 | 18 |
|  |  | 1 | 18 |

**Legend:** The weakness column denotes randomized LASSO stability selection ( weakness = 0.8) or LASSO stability selection (weakness = 1). A cut-off of 0.8 was used ensuring that only those variables present in at least 80% of all stability selection iterations, were included.

**Table S25: Beta estimates of LASSO Cox models.**

| **Men** | **Betas** | **Women** | **Betas** |
| --- | --- | --- | --- |
| Baseline hazard for 10 year HF risk | 0,154611 | Baseline hazard for 10 year HF risk | 0,134647 |
| p47_i0 | -0,01216 | p48_i0 | 0,005003 |
| p48_i0 | 0,006597 | p135_i0 | 0,02268 |
| p137_i0 | 0,040223 | p137_i0 | 0,047953 |
| p20154_i0 | -0,0121 | p20154_i0 | -0,01576 |
| p21003_i0 | 0,05887 | p21003_i0 | 0,067147 |
| p30070_i0 | 0,111615 | p30070_i0 | 0,074308 |
| p30720_i0 | 0,259068 | p30720_i0 | 0,444079 |
| p30750_i0 | 0,010873 | Pres_other_heart_disease | 0,832793 |
| Pres_other_heart_disease | 0,769384 | Pres_respiratory_disease | 0,195715 |
| Pres_hypertensive_disease | 0,11156 | Pres_hypertensive_disease | -0,0125 |
| p6149_i0Dentures | 0,16515 | p6149_i0Dentures | 0,174367 |
| p6146_i0Blue_badge | 0,251851 | p6146_i0Blue_badge | 0,176515 |
| p4080_i0_a0 | 0,005327 | p4080_i0_a0 | 0,006373 |
| p30140_i0 | 0,051543 | p30140_i0 | 0,055785 |
| p30710_i0 | 0,006399 | p30710_i0 | 0,013313 |
| p30600_i0 | -0,03048 | Pres_muscoskeletal_disease | 0,151009 |
| p23107_i0 | -0,00349 | p6153_i0Blood_pressure_medication | 0,127917 |
|  |  | p23108_i0 | -0,00244 |

**Table S26: Beta estimates of randomized LASSO Cox models.**

| **Men** | **Beta** | **Women** | **Beta** |
| --- | --- | --- | --- |
| Baseline hazard for 10 year HF risk | 0,155676 | Baseline hazard for 10 year HF risk | 0,133251 |
| p47_i0 | -0,01189 | p135_i0 | 0,02788 |
| p48_i0 | 0,01121 | p137_i0 | 0,049202 |
| p137_i0 | 0,044195 | p20154_i0 | -0,01599 |
| p20154_i0 | -0,0121 | p21003_i0 | 0,066127 |
| p21003_i0 | 0,059598 | p30070_i0 | 0,086303 |
| p30070_i0 | 0,115417 | p30720_i0 | 0,461078 |
| p30720_i0 | 0,256394 | Pres_other_heart_disease | 0,820534 |
| p30750_i0 | 0,011354 | Pres_respiratory_disease | 0,19209 |
| Pres_other_heart_disease | 0,785254 | Pres_hypertensive_disease | 0,012023 |
| Pres_hypertensive_disease | 0,127836 | p6149_i0Dentures | 0,188893 |
| p6149_i0Dentures | 0,1596 | p6146_i0Blue_badge | 0,229104 |
| p6146_i0Blue_badge | 0,242374 | p4080_i0_a0 | 0,006904 |
| p4080_i0_a0 | 0,005566 | p30140_i0 | 0,057123 |
| p30710_i0 | 0,009257 | p30710_i0 | 0,015931 |
| p30600_i0 | -0,03406 | Pres_muscoskeletal_disease | 0,153888 |
|  |  | p6153_i0Blood_pressure_medication | 0,186357 |

**Table S27: Beta estimates of ordinary Cox models.**

| **Men** | **Betas** | **Women** | **Betas** |
| --- | --- | --- | --- |
| Baseline hazard for 10 year HF risk | 0,075123 | Baseline hazard for 10 year HF risk | 0,292314 |
| p46_i0 | -0,00528 | p46_i0 | 0,001737 |
| p47_i0 | -0,00535 | p47_i0 | -0,00884 |
| p48_i0 | 0,010957 | p48_i0 | 0,010131 |
| p49_i0 | -0,00845 | p49_i0 | -0,00979 |
| p50_i0 | -0,00368 | p50_i0 | 0,006931 |
| p51_i0 | -0,01081 | p51_i0 | 0,000105 |
| p74_i0 | 0,000413 | p74_i0 | 0,024628 |
| p78_i0 | 0,077188 | p78_i0 | 0,093619 |
| p120_i0Yes | -0,0037 | p120_i0Yes | -0,07063 |
| p134_i0 | 0,000917 | p134_i0 | 0,073478 |
| p135_i0 | -0,00919 | p135_i0 | -0,0086 |
| p136_i0 | 0,027244 | p136_i0 | 0,029061 |
| p137_i0 | 0,019723 | p137_i0 | 0,02732 |
| p670_i0A house or bungalow | -0,02666 | p670_i0A house or bungalow | 0,120826 |
| p670_i0Care home | -0,51212 | p670_i0Care home | 0,795245 |
| p670_i0Mobile or temporary structure (i.e. caravan) | -0,06643 | p670_i0Mobile or temporary structure (i.e. caravan) | 1,044664 |
| p670_i0None of the above | -0,33113 | p670_i0None of the above | 0,162165 |
| p670_i0Sheltered accommodation | -0,12773 | p670_i0Sheltered accommodation | 0,522089 |
| p680_i0None of the above | 0,475238 | p680_i0None of the above | 0,12091 |
| p680_i0Own outright (by you or someone in your household) | 0,187934 | p680_i0Own outright (by you or someone in your household) | -0,02059 |
| p680_i0Own with a mortgage | 0,214103 | p680_i0Own with a mortgage | 0,030693 |
| p680_i0Pay part rent and part mortgage (shared ownership) | 0,651239 | p680_i0Pay part rent and part mortgage (shared ownership) | -0,43663 |
| p680_i0Rent - from local authority, local council, housing association | 0,290669 | p680_i0Rent - from local authority, local council, housing association | 0,023276 |
| p680_i0Rent - from private landlord or letting agency | 0,051155 | p680_i0Rent - from private landlord or letting agency | 0,106715 |
| p699_i0 | 0,000118 | p699_i0 | 0,002483 |
| p709_i0 | 0,007108 | p709_i0 | -0,00535 |
| p728_i0None | -0,04519 | p728_i0None | 0,170149 |
| p728_i0One | 0,002767 | p728_i0One | 0,008115 |
| p728_i0Three | -0,02725 | p728_i0Three | 0,008826 |
| p728_i0Two | -0,03399 | p728_i0Two | 0,007628 |
| p738_i031,000 to 51,999 | 0,017051 | p738_i031,000 to 51,999 | -0,08729 |
| p738_i052,000 to 100,000 | -0,16025 | p738_i052,000 to 100,000 | -0,16067 |
| p738_i0Greater than 100,000 | -0,33374 | p738_i0Greater than 100,000 | -0,2879 |
| p738_i0Less than 18,000 | 0,049485 | p738_i0Less than 18,000 | 0,049833 |
| p757_i0 | -0,00181 | p757_i0 | -0,00256 |
| p767_i0 | 0,002916 | p767_i0 | -0,00029 |
| p777_i0 | -0,01122 | p777_i0 | -0,02302 |
| p806_i0Never/rarely | 0,08947 | p806_i0Never/rarely | -0,02365 |
| p806_i0Sometimes | 0,055035 | p806_i0Sometimes | 0,047328 |
| p806_i0Usually | 0,076707 | p806_i0Usually | 0,034392 |
| p816_i0Never/rarely | -0,08246 | p816_i0Never/rarely | 0,095252 |
| p816_i0Sometimes | -0,08718 | p816_i0Sometimes | 0,101713 |
| p816_i0Usually | -0,06104 | p816_i0Usually | 0,129882 |
| p826_i0Never/rarely | -0,04725 | p826_i0Never/rarely | -0,00488 |
| p826_i0Sometimes | -0,01803 | p826_i0Sometimes | -0,02261 |
| p826_i0Usually | -0,213 | p826_i0Usually | 0,188345 |
| p845_i0 | 0,002639 | p845_i0 | 0,013423 |
| p864_i01 | 0,361273 | p864_i01 | -0,1912 |
| p864_i02 | 0,368105 | p864_i02 | -0,30276 |
| p864_i03 | 0,197992 | p864_i03 | -0,29862 |
| p864_i04 | 0,279434 | p864_i04 | -0,33064 |
| p864_i05 | 0,228563 | p864_i05 | -0,34352 |
| p864_i06 | 0,302069 | p864_i06 | -0,29902 |
| p864_i07 | 0,26334 | p864_i07 | -0,39923 |
| p864_i0Unable to walk | 0,201044 | p864_i0Unable to walk | 0,062996 |
| p874_i0 | -0,00034 | p874_i0 | -0,00047 |
| p884_i0 | -0,0146 | p884_i0 | -0,02653 |
| p894_i0 | 8,79E-05 | p894_i0 | -7,2E-05 |
| p904_i0 | 0,033431 | p904_i0 | -0,03057 |
| p914_i0 | 2,67E-05 | p914_i0 | -0,0005 |
| p924_i0None of the above | 0,510568 | p924_i0None of the above | -0,26843 |
| p924_i0Slow pace | 0,167226 | p924_i0Slow pace | 0,107505 |
| p924_i0Steady average pace | 0,204678 | p924_i0Steady average pace | 0,122564 |
| p943_i011-15 times a day | -0,10177 | p943_i011-15 times a day | 0,02582 |
| p943_i016-20 times a day | 0,019864 | p943_i016-20 times a day | 0,079905 |
| p943_i06-10 times a day | -0,05086 | p943_i06-10 times a day | -0,05058 |
| p943_i0More than 20 times a day | 0,033677 | p943_i0More than 20 times a day | 0,056858 |
| p943_i0None | -0,06617 | p943_i0None | -0,05362 |
| p971_i02-3 times in the last 4 weeks | 0,019079 | p971_i02-3 times in the last 4 weeks | -0,08217 |
| p971_i04-5 times a week | 0,035089 | p971_i04-5 times a week | -0,09379 |
| p971_i0Every day | 0,031718 | p971_i0Every day | 0,083823 |
| p971_i0Once a week | 0,02278 | p971_i0Once a week | -0,05105 |
| p971_i0Once in the last 4 weeks | -0,06996 | p971_i0Once in the last 4 weeks | -0,06746 |
| p981_i0Between 1.5 and 2 hours | 0,068334 | p981_i0Between 1.5 and 2 hours | -0,16949 |
| p981_i0Between 15 and 30 minutes | 0,04647 | p981_i0Between 15 and 30 minutes | 0,008294 |
| p981_i0Between 2 and 3 hours | 0,02159 | p981_i0Between 2 and 3 hours | 0,095785 |
| p981_i0Between 30 minutes and 1 hour | 0,00492 | p981_i0Between 30 minutes and 1 hour | 0,000182 |
| p981_i0Less than 15 minutes | -0,07228 | p981_i0Less than 15 minutes | -0,17976 |
| p981_i0Over 3 hours | 0,011863 | p981_i0Over 3 hours | 0,152383 |
| p1031_i0About once a month | 0,004675 | p1031_i0About once a month | -0,00732 |
| p1031_i0About once a week | 0,014985 | p1031_i0About once a week | 0,089034 |
| p1031_i0Almost daily | -0,01384 | p1031_i0Almost daily | 0,023065 |
| p1031_i0Never or almost never | 0,050288 | p1031_i0Never or almost never | 0,065403 |
| p1031_i0Once every few months | 0,03284 | p1031_i0Once every few months | -0,01823 |
| p1050_i0 | 0,004364 | p1050_i0 | 0,021833 |
| p1060_i0 | 0,00507 | p1060_i0 | -0,0066 |
| p1070_i0 | -0,01114 | p1070_i0 | -0,01118 |
| p1080_i0 | 0,013636 | p1080_i0 | 0,019215 |
| p1090_i0 | 0,017239 | p1090_i0 | 0,00324 |
| p1100_i0Most of the time | 0,03844 | p1100_i0Most of the time | 0,127439 |
| p1100_i0Never/rarely | -0,06241 | p1100_i0Never/rarely | -0,17595 |
| p1100_i0Often | -0,01015 | p1100_i0Often | -0,34835 |
| p1100_i0Sometimes | -0,10913 | p1100_i0Sometimes | -0,16421 |
| p1110_i0More than eight years | 0,048463 | p1110_i0More than eight years | 0,00111 |
| p1110_i0Never used mobile phone at least once per week | 0,080528 | p1110_i0Never used mobile phone at least once per week | 0,071813 |
| p1110_i0One year or less | 0,009317 | p1110_i0One year or less | 0,106841 |
| p1110_i0Two to four years | 0,03547 | p1110_i0Two to four years | 0,151168 |
| p1120_i030-59 mins | 0,048221 | p1120_i030-59 mins | -0,02511 |
| p1120_i04-6 hours | -0,09837 | p1120_i04-6 hours | -0,07354 |
| p1120_i05-29 mins | 0,002749 | p1120_i05-29 mins | 0,008207 |
| p1120_i0Less than 5mins | 0,018573 | p1120_i0Less than 5mins | -0,08063 |
| p1120_i0More than 6 hours | 0,147181 | p1120_i0More than 6 hours | 0,210877 |
| p1130_i0Always or almost always | 0,128248 | p1130_i0Always or almost always | 0,752671 |
| p1130_i0Less than half the time | -0,04899 | p1130_i0Less than half the time | 0,332068 |
| p1130_i0More than half the time | 0,075515 | p1130_i0More than half the time | 0,510679 |
| p1130_i0Never or almost never | 0,036883 | p1130_i0Never or almost never | 0,297262 |
| p1140_i0No | -0,02104 | p1140_i0No | 0,031916 |
| p1140_i0Yes, use is now less frequent | 0,013551 | p1140_i0Yes, use is now less frequent | 0,061039 |
| p1140_i0Yes, use is now more frequent | -0,03221 | p1140_i0Yes, use is now more frequent | 0,021084 |
| p1150_i0Left | -0,01285 | p1150_i0Left | 0,043678 |
| p1150_i0Right | 0,002867 | p1150_i0Right | 0,10024 |
| p1160_i0 | -0,00762 | p1160_i0 | 0,002651 |
| p1170_i0Not at all easy | -0,00098 | p1170_i0Not at all easy | -0,05832 |
| p1170_i0Not very easy | -0,0799 | p1170_i0Not very easy | -0,01831 |
| p1170_i0Very easy | -0,00918 | p1170_i0Very easy | 0,007204 |
| p1180_i0Definitely an 'evening' person | 0,014053 | p1180_i0Definitely an 'evening' person | -0,03407 |
| p1180_i0More a 'morning' than 'evening' person | -0,10411 | p1180_i0More a 'morning' than 'evening' person | 0,028089 |
| p1180_i0More an 'evening' than a 'morning' person | -0,04928 | p1180_i0More an 'evening' than a 'morning' person | 0,015764 |
| p1190_i0Sometimes | -0,04716 | p1190_i0Sometimes | 0,003952 |
| p1190_i0Usually | 0,003225 | p1190_i0Usually | 0,055756 |
| p1200_i0Sometimes | 0,034342 | p1200_i0Sometimes | -0,05457 |
| p1200_i0Usually | 0,024817 | p1200_i0Usually | -0,08489 |
| p1210_i0Yes | 0,024703 | p1210_i0Yes | -0,04019 |
| p1220_i0Often | -0,03372 | p1220_i0Often | 0,333322 |
| p1220_i0Sometimes | -0,00473 | p1220_i0Sometimes | -0,02225 |
| p1239_i0Only occasionally | -0,03416 | p1239_i0Only occasionally | 0,002739 |
| p1239_i0Yes, on most or all days | -0,12219 | p1239_i0Yes, on most or all days | 0,035858 |
| p1249_i0Just tried once or twice | 0,038532 | p1249_i0Just tried once or twice | -0,053 |
| p1249_i0Smoked occasionally | 0,055413 | p1249_i0Smoked occasionally | 0,034533 |
| p1249_i0Smoked on most or all days | 0,043426 | p1249_i0Smoked on most or all days | 0,003186 |
| p1259_i0Yes, more than one household member smokes | 0,003123 | p1259_i0Yes, more than one household member smokes | -0,01838 |
| p1259_i0Yes, one household member smokes | 0,03948 | p1259_i0Yes, one household member smokes | -0,0339 |
| p1269_i0 | 0,003003 | p1269_i0 | -0,00813 |
| p1279_i0 | 0,007609 | p1279_i0 | -0,00935 |
| p1289_i0 | -0,01075 | p1289_i0 | -0,01023 |
| p1299_i0 | -0,01595 | p1299_i0 | -0,01025 |
| p1309_i0 | 0,0012 | p1309_i0 | -0,02507 |
| p1319_i0 | 0,007351 | p1319_i0 | 0,006362 |
| p1329_i05-6 times a week | -0,207 | p1329_i05-6 times a week | 0,396608 |
| p1329_i0Less than once a week | 0,082118 | p1329_i0Less than once a week | -0,08358 |
| p1329_i0Never | 0,036396 | p1329_i0Never | -0,05079 |
| p1329_i0Once a week | 0,095356 | p1329_i0Once a week | -0,08742 |
| p1329_i0Once or more daily | 0,296894 | p1329_i0Once or more daily | -1,93662 |
| p1339_i05 or more times a week | -0,10907 | p1339_i05 or more times a week | 0,262562 |
| p1339_i0Less than once a week | -0,05284 | p1339_i0Less than once a week | 0,164101 |
| p1339_i0Never | 0,113176 | p1339_i0Never | 0,138362 |
| p1339_i0Once a week | -0,00731 | p1339_i0Once a week | 0,078974 |
| p1349_i05 or more times a week | -0,01467 | p1349_i05 or more times a week | -0,25634 |
| p1349_i0Less than once a week | 0,036117 | p1349_i0Less than once a week | -0,01208 |
| p1349_i0Never | 0,097514 | p1349_i0Never | -0,03211 |
| p1349_i0Once a week | 0,043689 | p1349_i0Once a week | -0,08252 |
| p1359_i05 or more times a week | 0,142285 | p1359_i05 or more times a week | 0,189452 |
| p1359_i0Less than once a week | 0,109155 | p1359_i0Less than once a week | 0,038067 |
| p1359_i0Never | -0,04455 | p1359_i0Never | -0,04029 |
| p1359_i0Once a week | -0,00869 | p1359_i0Once a week | -0,00375 |
| p1369_i05 or more times a week | 0,271708 | p1369_i05 or more times a week | -1,08179 |
| p1369_i0Less than once a week | -0,08375 | p1369_i0Less than once a week | -0,03734 |
| p1369_i0Never | -0,18782 | p1369_i0Never | -0,017 |
| p1369_i0Once a week | -0,01778 | p1369_i0Once a week | -0,05497 |
| p1379_i0Never | 0,015466 | p1379_i0Never | 0,005755 |
| p1379_i0Once a week | -0,0751 | p1379_i0Once a week | 0,1265 |
| p1379_i0Two or more times a week | -0,14579 | p1379_i0Two or more times a week | -0,04043 |
| p1389_i0Never | -0,02819 | p1389_i0Never | -0,04498 |
| p1389_i0Once a week | -0,02044 | p1389_i0Once a week | 0,049738 |
| p1389_i0Two or more times a week | -0,08047 | p1389_i0Two or more times a week | 0,030648 |
| p1408_i05-6 times a week | -0,01654 | p1408_i05-6 times a week | 0,197217 |
| p1408_i0Less than once a week | 0,011747 | p1408_i0Less than once a week | -0,01636 |
| p1408_i0Never | -0,06275 | p1408_i0Never | 0,345994 |
| p1408_i0Once a week | -0,08159 | p1408_i0Once a week | 0,044257 |
| p1408_i0Once or more daily | 0,024444 | p1408_i0Once or more daily | 0,116385 |
| p1418_i0Never/rarely have milk | 0,144757 | p1418_i0Never/rarely have milk | -0,13972 |
| p1418_i0Other type of milk | 0,180663 | p1418_i0Other type of milk | -0,14804 |
| p1418_i0Semi-skimmed | 0,08439 | p1418_i0Semi-skimmed | -0,04417 |
| p1418_i0Skimmed | 0,075742 | p1418_i0Skimmed | 0,031786 |
| p1418_i0Soya | 0,059258 | p1418_i0Soya | -0,03923 |
| p1428_i0Flora Pro-Active/Benecol | -0,2742 | p1428_i0Flora Pro-Active/Benecol | -0,5836 |
| p1428_i0Never/rarely use spread | -0,13693 | p1428_i0Never/rarely use spread | 0,013208 |
| p1428_i0Other type of spread/margarine | -0,07996 | p1428_i0Other type of spread/margarine | -0,10164 |
| p1438_i0 | -0,00336 | p1438_i0 | 0,003898 |
| p1448_i0Other type of bread | -0,02615 | p1448_i0Other type of bread | -0,09465 |
| p1448_i0White | 0,038271 | p1448_i0White | -0,11403 |
| p1448_i0Wholemeal or wholegrain | 0,047243 | p1448_i0Wholemeal or wholegrain | -0,14594 |
| p1458_i0 | -0,00537 | p1458_i0 | -0,00147 |
| p1468_i0Bran cereal (e.g. All Bran, Branflakes) | 0,072361 | p1468_i0Bran cereal (e.g. All Bran, Branflakes) | -0,10883 |
| p1468_i0Muesli | 0,056583 | p1468_i0Muesli | 0,037447 |
| p1468_i0Oat cereal (e.g. Ready Brek, porridge) | 0,033921 | p1468_i0Oat cereal (e.g. Ready Brek, porridge) | -0,02902 |
| p1468_i0Other (e.g. Cornflakes, Frosties) | 0,043618 | p1468_i0Other (e.g. Cornflakes, Frosties) | -0,03399 |
| p1478_i0Never/rarely | -0,04724 | p1478_i0Never/rarely | 0,111007 |
| p1478_i0Sometimes | -0,07034 | p1478_i0Sometimes | 0,13972 |
| p1478_i0Usually | -0,02642 | p1478_i0Usually | 0,244843 |
| p1488_i0 | -0,00851 | p1488_i0 | 0,001936 |
| p1498_i0 | -0,00227 | p1498_i0 | 0,023736 |
| p1508_i0Ground coffee (include espresso, filter etc) | 0,038478 | p1508_i0Ground coffee (include espresso, filter etc) | -0,1647 |
| p1508_i0Instant coffee | -0,04727 | p1508_i0Instant coffee | -0,03205 |
| p1508_i0Other type of coffee | 0,077374 | p1508_i0Other type of coffee | 0,146797 |
| p1518_i0Hot | -0,02709 | p1518_i0Hot | -0,09226 |
| p1518_i0Very hot | -0,06032 | p1518_i0Very hot | 0,002144 |
| p1518_i0Warm | -0,0063 | p1518_i0Warm | 0,116939 |
| p1528_i0 | 0,002346 | p1528_i0 | -0,01033 |
| p1538_i0Yes, because of illness | -0,07077 | p1538_i0Yes, because of illness | -0,10705 |
| p1538_i0Yes, because of other reasons | -0,03845 | p1538_i0Yes, because of other reasons | -0,12231 |
| p1548_i0Often | 0,113198 | p1548_i0Often | -0,05881 |
| p1548_i0Sometimes | 0,081559 | p1548_i0Sometimes | 0,019188 |
| p1558_i0Never | -0,08616 | p1558_i0Never | -0,02098 |
| p1558_i0Once or twice a week | 0,038042 | p1558_i0Once or twice a week | 0,039863 |
| p1558_i0One to three times a month | -0,07176 | p1558_i0One to three times a month | -0,10513 |
| p1558_i0Special occasions only | 0,089538 | p1558_i0Special occasions only | 0,008224 |
| p1558_i0Three or four times a week | 0,003248 | p1558_i0Three or four times a week | -0,11014 |
| p1568_i0 | -0,00683 | p1568_i0 | 0,006279 |
| p1578_i0 | -0,0003 | p1578_i0 | 0,001384 |
| p1588_i0 | 0,001739 | p1588_i0 | 0,001997 |
| p1598_i0 | 0,005284 | p1598_i0 | -0,01052 |
| p1608_i0 | 0,014034 | p1608_i0 | 0,018937 |
| p1618_i0No | 0,048357 | p1618_i0No | -0,02432 |
| p1618_i0Yes | -0,03381 | p1618_i0Yes | 0,017286 |
| p1628_i0Less nowadays | 0,114625 | p1628_i0Less nowadays | -0,0192 |
| p1628_i0More nowadays | 0,042894 | p1628_i0More nowadays | -0,02825 |
| p1647_i0England | -0,00625 | p1647_i0England | 0,0214 |
| p1647_i0Northern Ireland | 0,285327 | p1647_i0Northern Ireland | -0,15339 |
| p1647_i0Republic of Ireland | -0,03453 | p1647_i0Republic of Ireland | 0,115373 |
| p1647_i0Scotland | -0,0582 | p1647_i0Scotland | 0,108327 |
| p1647_i0Wales | 0,075456 | p1647_i0Wales | -0,17256 |
| p1677_i0Yes | 0,01443 | p1677_i0Yes | 0,012351 |
| p1687_i0Plumper | 0,014433 | p1687_i0Plumper | 0,034102 |
| p1687_i0Thinner | -0,03093 | p1687_i0Thinner | 0,06942 |
| p1697_i0Shorter | -0,02373 | p1697_i0Shorter | 0,080023 |
| p1697_i0Taller | -0,02376 | p1697_i0Taller | -0,00482 |
| p1707_i0Right-handed | 0,004688 | p1707_i0Right-handed | -0,13446 |
| p1707_i0Use both right and left hands equally | -0,0146 | p1707_i0Use both right and left hands equally | -0,35236 |
| p1717_i0Brown | -0,26628 | p1717_i0Brown | -0,12294 |
| p1717_i0Dark olive | -0,18945 | p1717_i0Dark olive | -0,6065 |
| p1717_i0Fair | -0,10974 | p1717_i0Fair | -0,35822 |
| p1717_i0Light olive | -0,11229 | p1717_i0Light olive | -0,29451 |
| p1717_i0Very fair | -0,12274 | p1717_i0Very fair | -0,38891 |
| p1727_i0Get moderately tanned | 0,073824 | p1727_i0Get moderately tanned | -0,02407 |
| p1727_i0Get very tanned | 0,0009 | p1727_i0Get very tanned | -0,10006 |
| p1727_i0Never tan, only burn | 0,06593 | p1727_i0Never tan, only burn | -0,01148 |
| p1737_i0 | -0,00589 | p1737_i0 | 0,00855 |
| p1747_i0Blonde | -0,0601 | p1747_i0Blonde | -0,02366 |
| p1747_i0Dark brown | 0,039484 | p1747_i0Dark brown | -0,00091 |
| p1747_i0Light brown | -0,0658 | p1747_i0Light brown | 0,046847 |
| p1747_i0Other | -0,17299 | p1747_i0Other | 0,231479 |
| p1747_i0Red | -0,0663 | p1747_i0Red | -0,10189 |
| p1757_i0Older than you are | -0,07656 | p1757_i0Older than you are | 0,047881 |
| p1757_i0Younger than you are | -0,03351 | p1757_i0Younger than you are | -0,07485 |
| p1767_i0Yes | 0,190532 | p1767_i0Yes | -0,33934 |
| p1777_i0Yes | -0,21181 | p1777_i0Yes | -0,17424 |
| p1787_i0Yes | -0,0656 | p1787_i0Yes | -0,0143 |
| p1797_i0Yes | -0,00736 | p1797_i0Yes | -0,04417 |
| p1807_i0 | 0,000814 | p1807_i0 | -4,9E-06 |
| p1835_i0Yes | 0,013339 | p1835_i0Yes | -0,01053 |
| p1873_i0 | -0,0011 | p1873_i0 | -0,00018 |
| p1883_i0 | 0,007553 | p1883_i0 | -0,01567 |
| p1920_i0Yes | 0,078346 | p1920_i0Yes | -0,03103 |
| p1930_i0Yes | 0,010195 | p1930_i0Yes | -0,05149 |
| p1940_i0Yes | -0,06029 | p1940_i0Yes | 0,042809 |
| p1950_i0Yes | -0,0412 | p1950_i0Yes | 0,071313 |
| p1960_i0Yes | -0,00601 | p1960_i0Yes | 0,011762 |
| p1970_i0Yes | 0,066861 | p1970_i0Yes | -0,01588 |
| p1980_i0Yes | -0,09069 | p1980_i0Yes | 0,032785 |
| p1990_i0Yes | 0,008509 | p1990_i0Yes | -0,05618 |
| p2000_i0Yes | 0,021251 | p2000_i0Yes | 0,015197 |
| p2010_i0Yes | 0,056887 | p2010_i0Yes | -0,04612 |
| p2020_i0Yes | -0,12009 | p2020_i0Yes | 0,036044 |
| p2030_i0Yes | 0,078344 | p2030_i0Yes | 0,004443 |
| p2040_i0Yes | -0,04621 | p2040_i0Yes | -0,0144 |
| p2050_i0Nearly every day | -0,03424 | p2050_i0Nearly every day | -0,26155 |
| p2050_i0Not at all | 0,02956 | p2050_i0Not at all | 0,052069 |
| p2050_i0Several days | -0,06482 | p2050_i0Several days | 0,002923 |
| p2060_i0Nearly every day | 0,092013 | p2060_i0Nearly every day | -0,22656 |
| p2060_i0Not at all | 0,05935 | p2060_i0Not at all | -0,13234 |
| p2060_i0Several days | 0,042271 | p2060_i0Several days | -0,06381 |
| p2070_i0Nearly every day | -0,12987 | p2070_i0Nearly every day | -0,4082 |
| p2070_i0Not at all | 0,003435 | p2070_i0Not at all | -0,22531 |
| p2070_i0Several days | -0,00232 | p2070_i0Several days | -0,23806 |
| p2080_i0Nearly every day | 0,090208 | p2080_i0Nearly every day | 0,067707 |
| p2080_i0Not at all | -0,03962 | p2080_i0Not at all | -0,04345 |
| p2080_i0Several days | 0,01409 | p2080_i0Several days | 0,00866 |
| p2090_i0Yes | 0,00804 | p2090_i0Yes | 0,065617 |
| p2100_i0Yes | 0,060321 | p2100_i0Yes | 0,107987 |
| p2110_i0About once a month | 0,019456 | p2110_i0About once a month | 0,076806 |
| p2110_i0About once a week | 0,011743 | p2110_i0About once a week | 0,203373 |
| p2110_i0Almost daily | 0,020858 | p2110_i0Almost daily | 0,114148 |
| p2110_i0Never or almost never | -0,01124 | p2110_i0Never or almost never | 0,2773 |
| p2110_i0Once every few months | -0,16614 | p2110_i0Once every few months | 0,128876 |
| p2129_i0Skip this section | 0,030866 | p2129_i0Skip this section | 0,140232 |
| p2139_i0 | -0,00171 | p2139_i0 | -0,0003 |
| p2149_i0 | 0,00015 | p2149_i0 | 0,003177 |
| p2159_i0Yes | -0,05423 | p2159_i0Yes | 0,47194 |
| p2178_i0Fair | 0,233134 | p2178_i0Fair | 0,234112 |
| p2178_i0Good | 0,104019 | p2178_i0Good | 0,1625 |
| p2178_i0Poor | 0,404998 | p2178_i0Poor | 0,286212 |
| p2188_i0Yes | 0,057156 | p2188_i0Yes | 0,142343 |
| p2207_i0Yes | -0,02232 | p2207_i0Yes | -0,04829 |
| p2217_i0 | 0,001066 | p2217_i0 | -0,00305 |
| p2227_i0Yes | 0,02539 | p2227_i0Yes | 0,004107 |
| p2237_i0Often | 0,043659 | p2237_i0Often | 0,026223 |
| p2237_i0Sometimes | 0,00779 | p2237_i0Sometimes | 0,025822 |
| p2247_i0Prefer not to answer | -0,7172 | p2247_i0Prefer not to answer | -0,18662 |
| p2247_i0Yes | 0,033567 | p2247_i0Yes | -0,11419 |
| p2257_i0Yes | -0,12656 | p2257_i0Yes | 0,093045 |
| p2267_i0Most of the time | 0,085496 | p2267_i0Most of the time | -0,0448 |
| p2267_i0Never/rarely | 0,096844 | p2267_i0Never/rarely | 0,167194 |
| p2267_i0Sometimes | 0,120476 | p2267_i0Sometimes | 0,057619 |
| p2277_i0 | 0,006146 | p2277_i0 | -0,00194 |
| p2296_i0No falls | 0,023977 | p2296_i0No falls | -0,09611 |
| p2296_i0Only one fall | 0,032465 | p2296_i0Only one fall | -0,14308 |
| p2306_i0Yes - gained weight | -0,03057 | p2306_i0Yes - gained weight | -0,02503 |
| p2306_i0Yes - lost weight | 0,054871 | p2306_i0Yes - lost weight | 0,030598 |
| p2316_i0Yes | 0,116459 | p2316_i0Yes | 0,14209 |
| p2335_i0Yes | 0,112978 | p2335_i0Yes | 0,115438 |
| p2345_i0Yes | -0,03867 | p2345_i0Yes | 0,055069 |
| p2443_i0Yes | -0,02187 | p2443_i0Yes | 0,256231 |
| p2453_i0Yes - you will be asked about this later by an interviewer | 0,169709 | p2453_i0Yes - you will be asked about this later by an interviewer | 0,178296 |
| p2463_i0Yes | -0,0113 | p2463_i0Yes | -0,16932 |
| p2473_i0Yes - you will be asked about this later by an interviewer | 0,131701 | p2473_i0Yes - you will be asked about this later by an interviewer | 0,224845 |
| p2492_i0Yes - you will be asked about this later by an interviewer | 0,102821 | p2492_i0Yes - you will be asked about this later by an interviewer | 0,291319 |
| p3061_i0_a0 | -0,00021 | p2674_i0Yes | 0,030664 |
| p3062_i0_a0 | 0,02463 | p2694_i0Yes | -0,07334 |
| p3063_i0_a0 | -0,03912 | p2714_i0 | 0,000724 |
| p3064_i0_a0 | 8,14E-06 | p2724_i0Not sure - had a hysterectomy | -0,0293 |
| p3089_i0Yes | -0,189 | p2724_i0Not sure - other reason | -0,3693 |
| p3090_i0Yes | -0,24385 | p2724_i0Yes | -0,07479 |
| p3143_i0 | -0,01142 | p2734_i0 | 0,010835 |
| p3144_i0 | -0,00127 | p2774_i0Yes | -0,13054 |
| p3146_i0 | 0,000912 | p2784_i0Yes | -0,0465 |
| p3147_i0 | -0,00771 | p2814_i0Yes | 0,079803 |
| p3148_i0 | 0,480926 | p2834_i0Not sure | -0,13114 |
| p3393_i0Yes | 0,055242 | p2834_i0Yes | -0,15044 |
| p3526_i0 | -0,00233 | p2844_i0Yes - you will be asked about this later by an interviewer | 0,028929 |
| p4079_i0_a0 | -0,0002 | p3061_i0_a0 | 0,000854 |
| p4080_i0_a0 | 0,006209 | p3062_i0_a0 | 0,022604 |
| p6138_i0[A levels/AS levels or equivalent] | -0,32466 | p3063_i0_a0 | -0,03658 |
| p6138_i0[College or University degree] | -0,22513 | p3064_i0_a0 | 0,000189 |
| p6138_i0[CSEs or equivalent] | -0,10309 | p3089_i0Yes | 0,266048 |
| p6138_i0[NVQ or HND or HNC or equivalent] | -0,11508 | p3090_i0Yes | -0,08374 |
| p6138_i0[Other professional qualifications eg: nursing, teaching] | -0,13135 | p3140_i0Unsure | 1,075526 |
| p6142_i0[Doing unpaid or voluntary work] | 0,577274 | p3140_i0Yes | -0,48156 |
| p6142_i0[Full or part-time student] | -0,02986 | p3143_i0 | -0,00183 |
| p6142_i0[In paid employment] | 0,258824 | p3144_i0 | 0,001334 |
| p6142_i0[Retired] | 0,223042 | p3146_i0 | 0,007763 |
| p6142_i0[Unable to work because of sickness or disability] | 0,347953 | p3147_i0 | -0,00014 |
| p6142_i0[Unemployed] | 0,442962 | p3148_i0 | -3,52551 |
| p20015_i0 | -0,00011 | p3393_i0Yes | -0,12904 |
| p20022_i0 | -0,05523 | p3526_i0 | -0,00234 |
| p20116_i0Never | -0,31322 | p4079_i0_a0 | -0,00274 |
| p20116_i0Previous | -0,29654 | p4080_i0_a0 | 0,008728 |
| p20117_i0Never | 0,092707 | p6138_i0[A levels/AS levels or equivalent] | -0,0847 |
| p20117_i0Previous | 0,151719 | p6138_i0[College or University degree] | -0,10154 |
| p20127_i0 | -0,00766 | p6138_i0[CSEs or equivalent] | 0,054563 |
| p20150_i0 | -0,09734 | p6138_i0[NVQ or HND or HNC or equivalent] | 0,21427 |
| p20151_i0 | 0,019173 | p6138_i0[Other professional qualifications eg: nursing, teaching] | -0,05027 |
| p20153_i0 | 0,015858 | p6142_i0[Doing unpaid or voluntary work] | -0,25929 |
| p20154_i0 | 0,004366 | p6142_i0[Full or part-time student] | -0,05229 |
| p20160_i0Yes | 0,05607 | p6142_i0[In paid employment] | -0,11894 |
| p20256 | 0,156805 | p6142_i0[Retired] | -0,086 |
| p20257 | 0,029283 | p6142_i0[Unable to work because of sickness or disability] | 0,079388 |
| p20258 | -0,02121 | p6142_i0[Unemployed] | 0,501733 |
| p21000_i0British | 0,109904 | p20015_i0 | -0,01576 |
| p21000_i0Indian | 0,098513 | p20022_i0 | -0,04085 |
| p21000_i0Irish | -0,16426 | p20116_i0Never | -0,26622 |
| p21000_i0Other | 0,066029 | p20116_i0Previous | -0,17204 |
| p21001_i0 | 0,069212 | p20117_i0Never | 0,240903 |
| p21002_i0 | 2,35E-05 | p20117_i0Previous | -0,00698 |
| p21003_i0 | 0,069928 | p20127_i0 | -0,0168 |
| p21022 | -0,00012 | p20150_i0 | 0,035967 |
| p22033_i0 | -0,00046 | p20151_i0 | 0,005259 |
| p22034_i0 | 0,000445 | p20153_i0 | 0,349204 |
| p22037_i0 | -5,3E-05 | p20154_i0 | -0,00971 |
| p22038_i0 | -5E-05 | p20160_i0Yes | 0,024569 |
| p22039_i0 | -6,9E-05 | p20256 | -0,01999 |
| p22040_i0 | 4,02E-05 | p20257 | 0,06775 |
| p23098_i0 | -0,0574 | p20258 | -0,01496 |
| p23099_i0 | 0,152967 | p21000_i0British | 0,055072 |
| p23100_i0 | -0,00707 | p21000_i0Indian | 0,146606 |
| p23101_i0 | 0,099143 | p21000_i0Irish | -0,02979 |
| p23102_i0 | -0,00775 | p21000_i0Other | -0,03152 |
| p23104_i0 | -0,07604 | p21001_i0 | -0,05868 |
| p23105_i0 | 0,000165 | p21002_i0 | 0,039086 |
| p23106_i0 | 0,002415 | p21003_i0 | 0,081532 |
| p23107_i0 | -0,01059 | p21022 | -0,0048 |
| p23108_i0 | 0,006806 | p22033_i0 | 0,02004 |
| p23109_i0 | 0,000182 | p22034_i0 | -0,00068 |
| p23110_i0 | -0,00162 | p22037_i0 | 0,000174 |
| p23111_i0 | 0,079175 | p22038_i0 | 0,000116 |
| p23112_i0 | -0,27872 | p22039_i0 | 0,000111 |
| p23113_i0 | -0,00679 | p22040_i0 | -0,0001 |
| p23114_i0 | 0,013108 | p23098_i0 | 0,002864 |
| p23115_i0 | -0,16383 | p23099_i0 | -0,10738 |
| p23116_i0 | 0,527724 | p23100_i0 | 0,124093 |
| p23117_i0 | 0,486884 | p23101_i0 | -0,05781 |
| p23118_i0 | -0,50061 | p23102_i0 | 0,018805 |
| p23119_i0 | 0,035364 | p23104_i0 | 0,167502 |
| p23120_i0 | -0,6072 | p23105_i0 | 0,002505 |
| p23121_i0 | -0,47242 | p23106_i0 | 0,000923 |
| p23122_i0 | 0,313817 | p23107_i0 | 0,008883 |
| p23123_i0 | -0,02792 | p23108_i0 | -0,00818 |
| p23124_i0 | 0,389416 | p23109_i0 | -0,00154 |
| p23125_i0 | -0,10226 | p23110_i0 | 0,001636 |
| p23126_i0 | 0,026081 | p23111_i0 | -0,09372 |
| p23127_i0 | -0,07455 | p23112_i0 | -0,25232 |
| p23128_i0 | 0,036609 | p23113_i0 | 0,280878 |
| p23129_i0 | 0,085486 | p23114_i0 | -0,48643 |
| p23130_i0 | -0,09145 | p23115_i0 | 0,15303 |
| p24003 | -0,00311 | p23116_i0 | -0,5709 |
| p24004 | 0,006646 | p23117_i0 | -0,03707 |
| p24005 | -0,0195 | p23118_i0 | -0,02464 |
| p24006 | -0,01242 | p23119_i0 | 0,045048 |
| p24007 | -0,32288 | p23120_i0 | -0,66269 |
| p24008 | 0,026795 | p23121_i0 | -1,18081 |
| p24009 | -5,4E-06 | p23122_i0 | 1,345647 |
| p24010 | -0,23624 | p23123_i0 | -0,06086 |
| p24011 | 7,84E-07 | p23124_i0 | 0,506009 |
| p24012 | 0,607511 | p23125_i0 | -0,38009 |
| p24013 | -4,2E-08 | p23126_i0 | -0,27117 |
| p24014Yes | -0,16012 | p23127_i0 | 0,063461 |
| p24015 | 8,55E-05 | p23128_i0 | -0,2063 |
| p24016 | -0,00221 | p23129_i0 | -1,01759 |
| p24017 | 0,014337 | p23130_i0 | 0,773422 |
| p24018 | 0,005473 | p24003 | 0,002072 |
| p24019 | 0,000361 | p24004 | -0,00198 |
| p24020 | -0,10945 | p24005 | 0,046567 |
| p24021 | 0,253564 | p24006 | -0,05488 |
| p24022 | -0,1266 | p24007 | -0,01101 |
| p24023 | 0,264391 | p24008 | -0,03774 |
| p24024 | -0,26731 | p24009 | -7,7E-06 |
| p24500_i0 | 0,004351 | p24010 | 0,170511 |
| p24501_i0 | -0,00034 | p24011 | -1,3E-06 |
| p24502_i0 | 0,009073 | p24012 | -1,38206 |
| p24503_i0 | -0,00129 | p24013 | 3,18E-08 |
| p24504_i0 | 0,003201 | p24014Yes | 0,14267 |
| p24505_i0 | 7,3E-05 | p24015 | -0,00048 |
| p24506_i0 | 0,000447 | p24016 | -0,02687 |
| p24507_i0 | 0,001736 | p24017 | 0,04234 |
| p24508_i0 | -0,00097 | p24018 | -0,01047 |
| p26410 | 0,016851 | p24019 | 0,030516 |
| p26411 | -0,52082 | p24020 | 0,903905 |
| p26412 | -2,69217 | p24021 | -0,82021 |
| p26413 | 0,000293 | p24022 | 0,281035 |
| p26414 | -0,00102 | p24023 | -0,5165 |
| p26415 | -0,00597 | p24024 | 0,144821 |
| p26416 | 0,013435 | p24500_i0 | -0,00938 |
| p26417 | -0,00307 | p24501_i0 | -0,01173 |
| p30000_i0 | -0,27744 | p24502_i0 | 0,011911 |
| p30010_i0 | 0,342407 | p24503_i0 | -0,00083 |
| p30020_i0 | -0,04528 | p24504_i0 | 0,001452 |
| p30030_i0 | -0,03917 | p24505_i0 | -0,0101 |
| p30040_i0 | 0,008273 | p24506_i0 | 0,006909 |
| p30050_i0 | 0,040892 | p24507_i0 | -0,00016 |
| p30060_i0 | -0,0118 | p24508_i0 | -0,00084 |
| p30070_i0 | 0,087743 | p26410 | -0,00579 |
| p30080_i0 | 0,001281 | p26411 | 0,345198 |
| p30090_i0 | -1,43763 | p26412 | -0,40714 |
| p30100_i0 | 0,051029 | p26413 | 0,079005 |
| p30110_i0 | 0,079909 | p26414 | 0,004265 |
| p30120_i0 | 0,291415 | p26415 | 7,11E-05 |
| p30130_i0 | 0,112633 | p26416 | -0,05795 |
| p30140_i0 | 0,321765 | p26417 | -0,00143 |
| p30150_i0 | -0,03199 | p30000_i0 | -0,12923 |
| p30160_i0 | 0,327556 | p30010_i0 | 0,695767 |
| p30170_i0 | 0,126248 | p30020_i0 | -0,53006 |
| p30180_i0 | -0,06312 | p30030_i0 | 0,114477 |
| p30190_i0 | -0,04401 | p30040_i0 | 0,003416 |
| p30200_i0 | -0,05843 | p30050_i0 | 0,091426 |
| p30210_i0 | -0,02948 | p30060_i0 | 0,121599 |
| p30220_i0 | -0,02256 | p30070_i0 | 0,049689 |
| p30230_i0 | -0,07544 | p30080_i0 | -0,0004 |
| p30240_i0 | -0,26683 | p30090_i0 | -0,60868 |
| p30250_i0 | 3,578223 | p30100_i0 | 0,048422 |
| p30260_i0 | -0,00568 | p30110_i0 | -0,08936 |
| p30270_i0 | 0,011931 | p30120_i0 | 0,110878 |
| p30280_i0 | -1,3694 | p30130_i0 | 0,170632 |
| p30290_i0 | 0,490493 | p30140_i0 | 0,146863 |
| p30300_i0 | 2,068286 | p30150_i0 | 0,341244 |
| p30510_i0 | 5,52E-06 | p30160_i0 | 2,202412 |
| p30520_i0 | 0,0003 | p30170_i0 | 1,548431 |
| p30530_i0 | -0,00104 | p30180_i0 | 0,002181 |
| p30600_i0 | -0,01814 | p30190_i0 | 0,01114 |
| p30610_i0 | -0,00064 | p30200_i0 | 0,007311 |
| p30620_i0 | -0,00789 | p30210_i0 | -0,03898 |
| p30630_i0 | -0,32131 | p30220_i0 | -0,13136 |
| p30640_i0 | -0,38651 | p30230_i0 | -0,03494 |
| p30650_i0 | 0,007216 | p30240_i0 | -0,37591 |
| p30660_i0 | 0,045592 | p30250_i0 | 5,558086 |
| p30670_i0 | 0,016753 | p30260_i0 | -0,00141 |
| p30680_i0 | 0,402574 | p30270_i0 | 0,015929 |
| p30690_i0 | 0,302419 | p30280_i0 | -0,89809 |
| p30700_i0 | -0,00445 | p30290_i0 | 0,733775 |
| p30710_i0 | 0,005074 | p30300_i0 | -5,15885 |
| p30720_i0 | 0,490639 | p30510_i0 | 1,09E-05 |
| p30730_i0 | 0,000544 | p30520_i0 | -0,00012 |
| p30740_i0 | 0,015332 | p30530_i0 | -0,00183 |
| p30750_i0 | 0,012093 | p30600_i0 | -0,01555 |
| p30760_i0 | -0,10207 | p30610_i0 | 0,001179 |
| p30770_i0 | 0,006327 | p30620_i0 | -0,00734 |
| p30780_i0 | -0,18152 | p30630_i0 | -0,25206 |
| p30790_i0 | 0,001442 | p30640_i0 | -0,60204 |
| p30810_i0 | 0,270257 | p30650_i0 | 0,004536 |
| p30830_i0 | 0,003777 | p30660_i0 | 0,066705 |
| p30840_i0 | 0,007357 | p30670_i0 | 0,024916 |
| p30850_i0 | 0,000348 | p30680_i0 | -0,33143 |
| p30860_i0 | -0,0055 | p30690_i0 | -0,09163 |
| p30870_i0 | -0,06063 | p30700_i0 | -0,00252 |
| p30880_i0 | 0,001066 | p30710_i0 | 0,008255 |
| p30890_i0 | -0,00165 | p30720_i0 | 0,417704 |
| p3079_i0Yes | -0,58644 | p30730_i0 | 0,000722 |
| p3088_i0Unsure | 0,072162 | p30740_i0 | 0,044278 |
| p3088_i0Yes | 0,03637 | p30750_i0 | -0,00041 |
| p6145_i0Death_of_a_close_relative | 0,006185 | p30760_i0 | 0,335202 |
| p6145_i0Financial_difficulties | -0,00556 | p30770_i0 | -0,00251 |
| p6145_i0Marital_separation_divorce | 0,155078 | p30780_i0 | 0,257598 |
| p6145_i0Death_of_a_spouse_or_partner | 0,192409 | p30790_i0 | 0,000763 |
| p6145_i0Serious_illness__injury_or_assault_to_yourself | -0,04334 | p30810_i0 | 0,449163 |
| p6145_i0Serious_illness__injury_or_assault_of_a_close_relative | -0,06416 | p30830_i0 | 0,000898 |
| p6146_i0Disability_living_allowance | 0,030958 | p30840_i0 | -0,01345 |
| p6146_i0Blue_badge | 0,084575 | p30850_i0 | 0,012798 |
| p6146_i0Attendance_allowance | 0,189431 | p30860_i0 | 0,015399 |
| p6149_i0Dentures | 0,034459 | p30870_i0 | -0,01653 |
| p6149_i0Bleeding_gums | -0,0377 | p30880_i0 | 0,000589 |
| p6149_i0Mouth_ulcers | -0,07961 | p30890_i0 | 0,001084 |
| p6149_i0Toothache | 0,097055 | p3079_i0Yes | 0,403713 |
| p6149_i0Loose_teeth | -0,06187 | p3088_i0Unsure | -0,30942 |
| p6149_i0Painful_gums | -0,016 | p3088_i0Yes | 0,04753 |
| p6150_i0High_blood_pressure | 0,090438 | p6145_i0Death_of_a_close_relative | 0,083591 |
| p6150_i0Stroke | 0,017362 | p6145_i0Financial_difficulties | 0,082937 |
| p6150_i0Angina | 0,226314 | p6145_i0Marital_separation_divorce | -0,13197 |
| p6152_i0Asthma | -0,16118 | p6145_i0Death_of_a_spouse_or_partner | -0,01073 |
| p6152_i0Blood_clot_in_the_leg_DVT | 0,038599 | p6145_i0Serious_illness__injury_or_assault_to_yourself | 0,015506 |
| p6152_i0Emphysema_chronic_bronchitis | 0,017064 | p6145_i0Serious_illness__injury_or_assault_of_a_close_relative | -0,11113 |
| p6152_i0Blood_clot_in_the_lung | -0,22728 | p6146_i0Disability_living_allowance | -0,27476 |
| p6152_i0Hayfever__allergic_rhinitis_or_eczema | -0,03166 | p6146_i0Blue_badge | 0,153705 |
| p6154_i0Paracetamol | -0,0375 | p6146_i0Attendance_allowance | 0,15365 |
| p6154_i0Aspirin | 0,09181 | p6149_i0Dentures | 0,046114 |
| p6154_i0Ibuprofen_eg_Nurofen | -0,04069 | p6149_i0Bleeding_gums | -0,10502 |
| p6154_i0Omeprazole_eg_Zanprol | -0,17162 | p6149_i0Mouth_ulcers | -0,06152 |
| p6154_i0Ranitidine_eg_Zantac | 0,057683 | p6149_i0Toothache | 0,01325 |
| p6154_i0Laxatives_eg_Dulcolax__Senokot | -0,03148 | p6149_i0Loose_teeth | 0,007656 |
| p6155_i0Multivitamins___minerals | -0,06443 | p6149_i0Painful_gums | 0,130856 |
| p6155_i0Vitamin_C | -0,14408 | p6150_i0High_blood_pressure | 0,05192 |
| p6155_i0Vitamin_D | 0,053805 | p6150_i0Stroke | 0,239846 |
| p6155_i0Vitamin_B | 0,058506 | p6150_i0Angina | -0,02927 |
| p6155_i0Folic_acid_or_Folate_Vit_B9 | 0,032956 | p6152_i0Asthma | -0,04587 |
| p6155_i0Vitamin_E | 0,146451 | p6152_i0Blood_clot_in_the_leg_DVT | 0,079063 |
| p6155_i0Vitamin_A | 0,058981 | p6152_i0Emphysema_chronic_bronchitis | -0,244 |
| p6159_i0Back_pain | -0,01595 | p6152_i0Blood_clot_in_the_lung | 0,014469 |
| p6159_i0Knee_pain | 0,005575 | p6152_i0Hayfever__allergic_rhinitis_or_eczema | -0,03937 |
| p6159_i0Headache | -0,09897 | p6153_i0Blood_pressure_medication | 0,071971 |
| p6159_i0Neck_or_shoulder_pain | -0,01923 | p6153_i0Hormone_replacement_therapy | 0,056898 |
| p6159_i0Stomach_or_abdominal_pain | -0,08897 | p6153_i0Cholesterol_lowering_medication | -0,14632 |
| p6159_i0Hip_pain | -0,04119 | p6153_i0Oral_contraceptive_pill_or_minipill | 0,020121 |
| p6159_i0Pain_all_over_the_body | -0,13143 | p6153_i0Insulin | 0,019898 |
| p6159_i0Facial_pain | -0,07817 | p6154_i0Paracetamol | -0,02628 |
| p6160_i0Sports_club_or_gym | 0,047826 | p6154_i0Aspirin | 0,035257 |
| p6160_i0Pub_or_social_club | 0,055743 | p6154_i0Ibuprofen_eg_Nurofen | -0,22823 |
| p6160_i0Other_group_activity | 0,070677 | p6154_i0Omeprazole_eg_Zanprol | -0,01617 |
| p6160_i0Religious_group | 0,044831 | p6154_i0Ranitidine_eg_Zantac | 0,035937 |
| p6160_i0Adult_education_class | 0,158506 | p6154_i0Laxatives_eg_Dulcolax__Senokot | -0,10119 |
| p6162_i0Car_motor_vehicle | -0,04266 | p6155_i0Multivitamins___minerals | -0,07041 |
| p6162_i0Walk | 0,048322 | p6155_i0Vitamin_C | 0,155767 |
| p6162_i0Public_transport | -0,07289 | p6155_i0Vitamin_D | -0,06738 |
| p6162_i0Cycle | 0,107099 | p6155_i0Vitamin_B | -0,13782 |
| p6164_i0Walking_for_pleasure_not_as_a_means_of_transport | -0,03761 | p6155_i0Folic_acid_or_Folate_Vit_B9 | 0,116154 |
| p6164_i0Strenuous_sports | -0,05487 | p6155_i0Vitamin_E | 0,056475 |
| p6164_i0Light_DIY_eg_pruning__watering_the_lawn | 0,052089 | p6155_i0Vitamin_A | -0,25531 |
| p6164_i0Other_exercises_eg_swimming__cycling__keep_fit__bowling | -0,03283 | p6159_i0Back_pain | 0,025809 |
| p6164_i0Heavy_DIY_eg_weeding__lawn_mowing__carpentry__digging | -0,01484 | p6159_i0Knee_pain | -0,02354 |
| p6177_i0Blood_pressure_medication | 0,032572 | p6159_i0Headache | 0,005992 |
| p6177_i0Cholesterol_lowering_medication | -0,03379 | p6159_i0Neck_or_shoulder_pain | 0,001561 |
| p6177_i0Insulin | 0,096697 | p6159_i0Stomach_or_abdominal_pain | -0,05819 |
| p6179_i0Fish_oil_including_cod_liver_oil | 0,015363 | p6159_i0Hip_pain | -0,07189 |
| p6179_i0Glucosamine | 0,037209 | p6159_i0Pain_all_over_the_body | -0,25651 |
| p6179_i0Calcium | 0,040677 | p6159_i0Facial_pain | -0,10741 |
| p6179_i0Iron | -0,04937 | p6160_i0Sports_club_or_gym | -0,08586 |
| p6179_i0Zinc | -0,15997 | p6160_i0Pub_or_social_club | 0,00675 |
| p6179_i0Selenium | -0,17212 | p6160_i0Other_group_activity | 0,010924 |
| p6139_i0gas | -0,08717 | p6160_i0Religious_group | -0,14991 |
| p6139_i0cooker | 0,042974 | p6160_i0Adult_education_class | -0,0483 |
| p6139_i0fuel | -0,09686 | p6162_i0Car_motor_vehicle | 0,07242 |
| Pres_infections_parasitic_diseases | 0,04311 | p6162_i0Walk | -0,06613 |
| Pres_respiratory_disease | 0,086315 | p6162_i0Public_transport | -0,00807 |
| Pres_digestive_disease | 0,048956 | p6162_i0Cycle | -0,11987 |
| Pres_skin_disease | -0,02851 | p6164_i0Walking_for_pleasure_not_as_a_means_of_transport | -0,07218 |
| Pres_muscoskeletal_disease | -0,03057 | p6164_i0Strenuous_sports | -0,29597 |
| Pres_genitourinary_disease | -0,03491 | p6164_i0Light_DIY_eg_pruning__watering_the_lawn | -0,01821 |
| Pres_neoplasms | 0,045556 | p6164_i0Other_exercises_eg_swimming__cycling__keep_fit__bowling | 0,060822 |
| Pres_blood_bloodorgan_immune_disease | 0,203195 | p6164_i0Heavy_DIY_eg_weeding__lawn_mowing__carpentry__digging | -0,10637 |
| Pres_other_endocrine_metabolic_disease | -0,01776 | p6179_i0Fish_oil_including_cod_liver_oil | 0,080749 |
| Pres_mental_disease | 0,10432 | p6179_i0Glucosamine | 0,15334 |
| Pres_nervous_system_disease | 0,091779 | p6179_i0Calcium | -0,1303 |
| Pres_eye_adnea_disease | 0,015288 | p6179_i0Iron | 0,002241 |
| Pres_ear_mastoid_disease | 0,166156 | p6179_i0Zinc | 0,00364 |
| Pres_other_heart_disease | 0,721175 | p6179_i0Selenium | 0,21513 |
| Pres_diabetes | -0,03185 | p6139_i0gas | -0,17844 |
| Pres_hypertensive_disease | -0,01674 | p6139_i0cooker | 0,085443 |
| Pres_obesity | -0,11036 | p6139_i0fuel | 0,02495 |
| Pres_other_circulatory_diseases | 0,091518 | Pres_infections_parasitic_diseases | 0,011406 |
| Pres_thyroid_disorder | -0,0906 | Pres_respiratory_disease | 0,163888 |
|  |  | Pres_digestive_disease | -0,01732 |
|  |  | Pres_skin_disease | 0,115249 |
|  |  | Pres_muscoskeletal_disease | 0,056604 |
|  |  | Pres_genitourinary_disease | 0,069494 |
|  |  | Pres_pregnancy_complications | -0,13002 |
|  |  | Pres_neoplasms | 0,093708 |
|  |  | Pres_blood_bloodorgan_immune_disease | 0,245213 |
|  |  | Pres_other_endocrine_metabolic_disease | 0,11593 |
|  |  | Pres_mental_disease | -0,09224 |
|  |  | Pres_nervous_system_disease | -0,035 |
|  |  | Pres_eye_adnea_disease | 0,08539 |
|  |  | Pres_ear_mastoid_disease | -0,26398 |
|  |  | Pres_other_heart_disease | 0,856347 |
|  |  | Pres_diabetes | -0,50277 |
|  |  | Pres_hypertensive_disease | 0,002141 |
|  |  | Pres_obesity | -0,28914 |
|  |  | Pres_other_circulatory_diseases | 0,117303 |
|  |  | Pres_thyroid_disorder | -0,14698 |
|  |  |  |  |

**Table S28: Performance metrices of ML models trained on variables remaining after LASSO stability selection.**

| **Model** | **Sex** | **Full data** | **Subdata** |
| --- | --- | --- | --- |
| RSF | Men | 0.78 | 0.76 |
|  | Women | 0.81 | 0.78 |
| XGBoost | Men | 0.78 | 0.77 |
|  | Women | 0.82 | 0.78 |

**Legend:** Performance metrices are obtained through external validation of the hold-out set on the train datasets. Performance metrices are defined as C-indices. RSF; random survival forest, XGBoost, eXtreme Gradient Boosting.

**Figure S1: Overview of data-flow.**


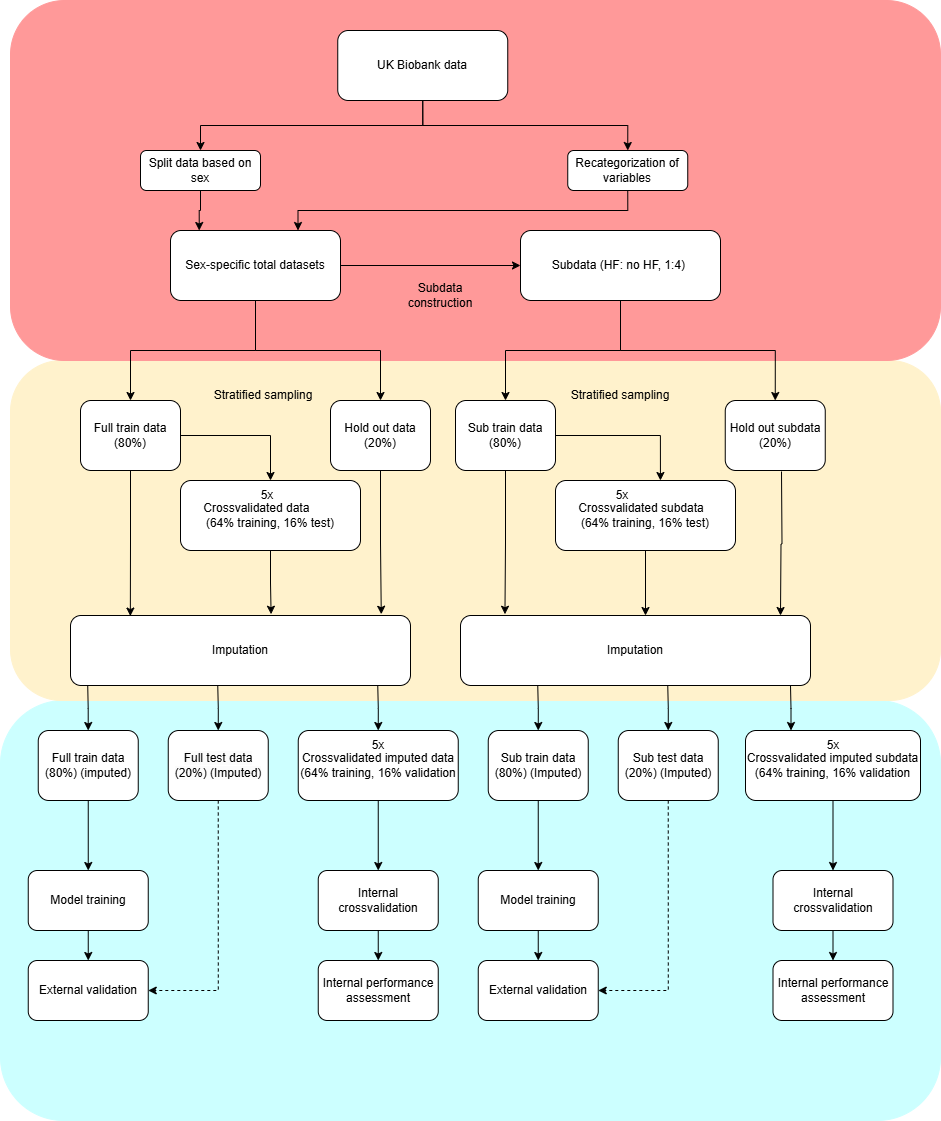


**Legend:** The overview of data-flow is split up in: preprocessing (red), imputation (orange), and both internal- and external crossvalidation (blue).

**Figure S2: Net benefit curves for men in the subset.**


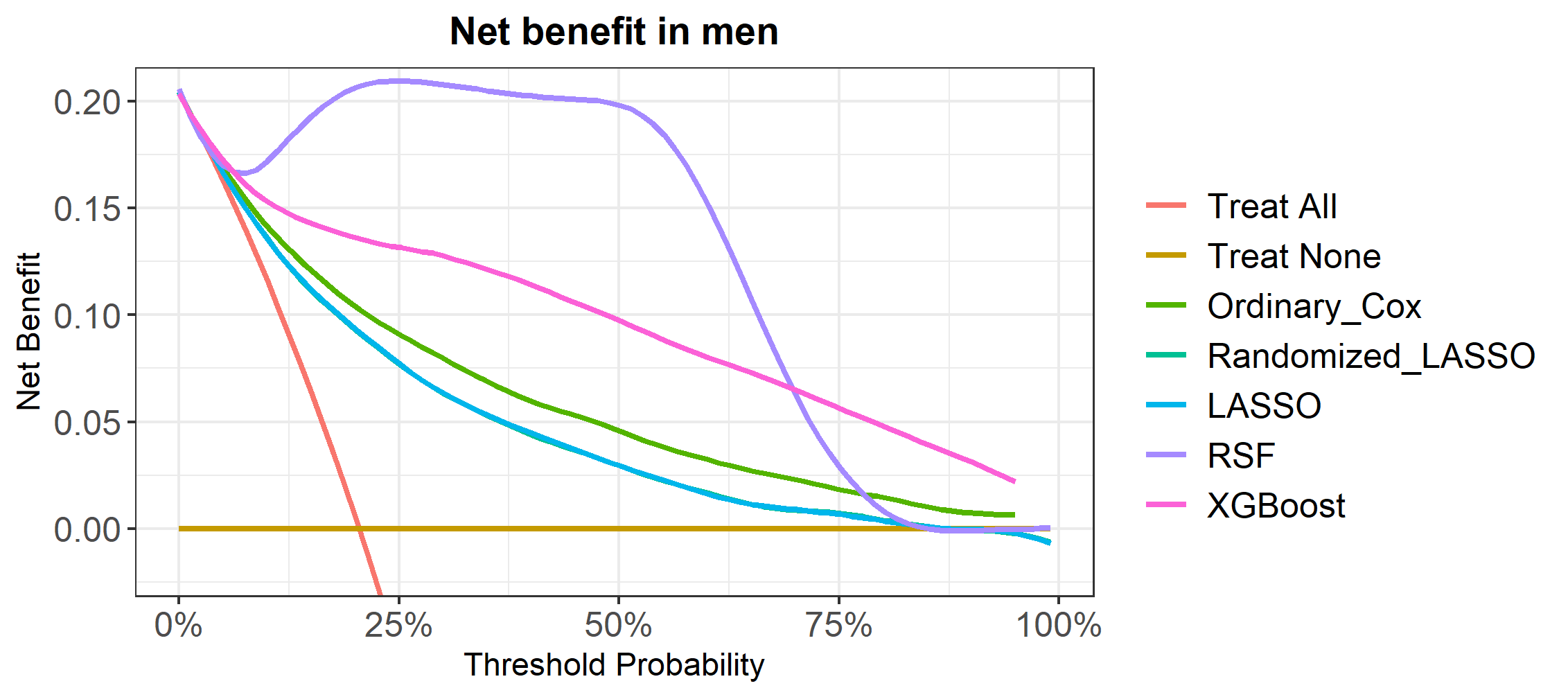


**Legend:** Decision curves showcasing net benefit for each prediction model.

**Figure S3: Net benefit curves for women in the subset.**


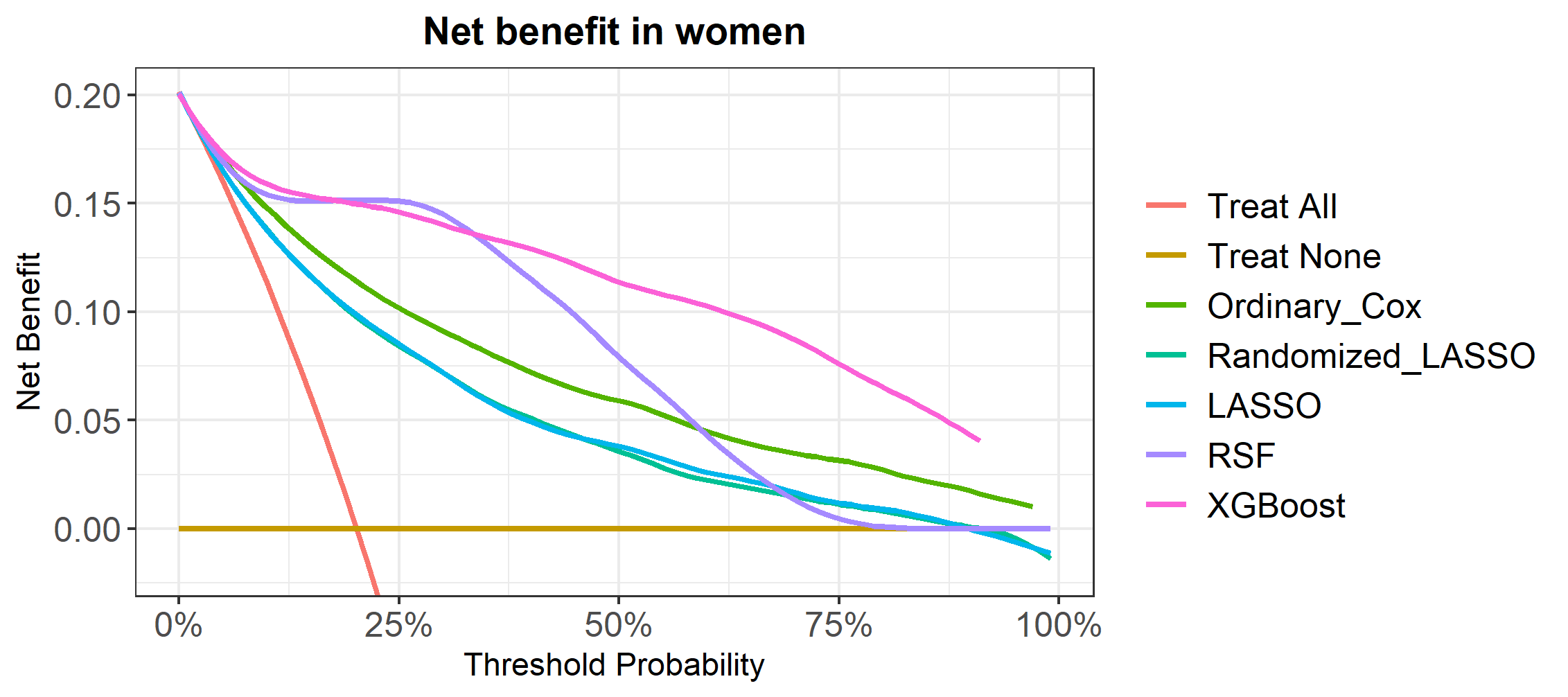


**Legend:** Decision curves showcasing net benefit for each prediction model.

**Figure S4:** **Calibration plots for subset men.**

**
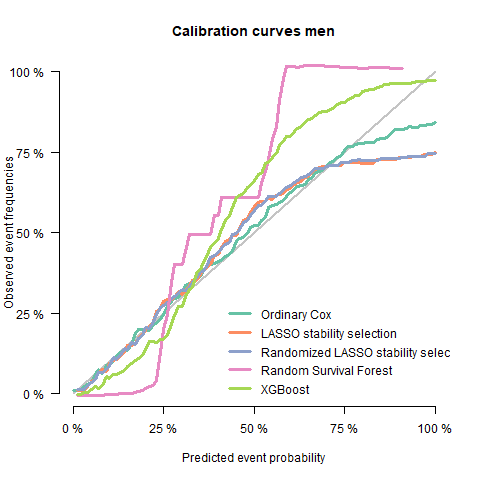
**

**Legend:** Calibration plots for each predictor model in subset men. Both RSF and XGBoost tend to underestimate the risk of HF, although performance is worse in RSF. The Cox PH models perform well but overestimate the probability of an event after ~ 70% predicted event probability.

**Figure S5: Calibration plots for subset women.**

**
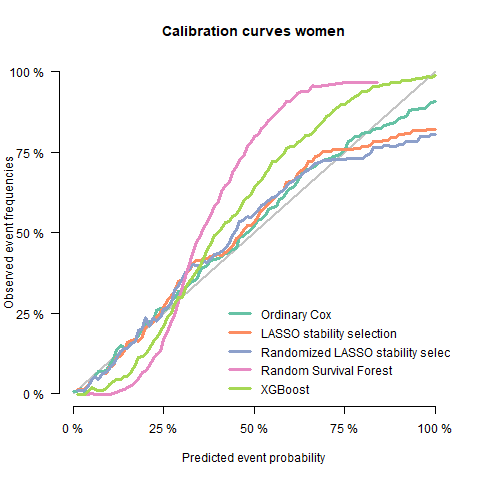
**

**Legend:** Calibration plots for each predictor model in subset women. RSF overestimates when predicted event probability is low and underestimates when predicted event probability is above ~35%. Overall, XGBoost shows slight underestimation, and overall, the CoxPH models show good calibration, with slight overestimation at predicted event probability above ~75%.

**Figure S6: Permutation importance for RSF, stratified by sex, in the subset data.**

**
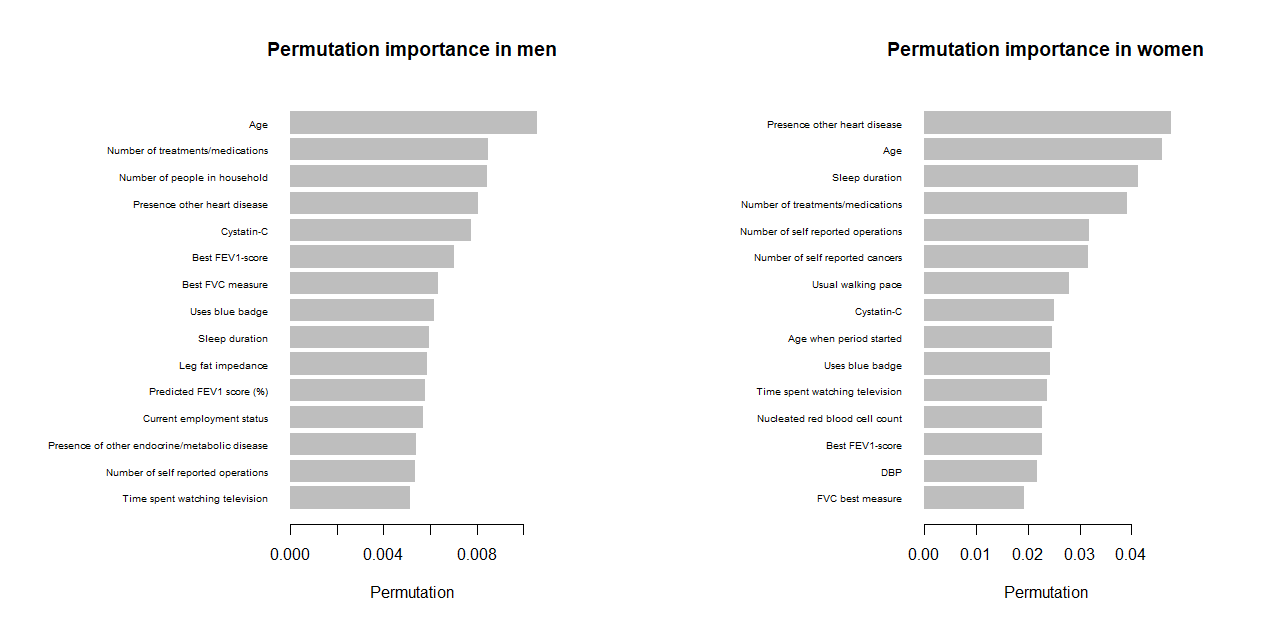
**

**Legend:** Permutation importance of RSF models in the subset data, limited to the 15 most important predictors. Permutation scores are overall higher for women than men. The absolute impact of age on prediction accuracy is more pronounced in men than women.

**Figure S7: Gain importance for XGBoost, stratified by sex, in the subset data.**

**
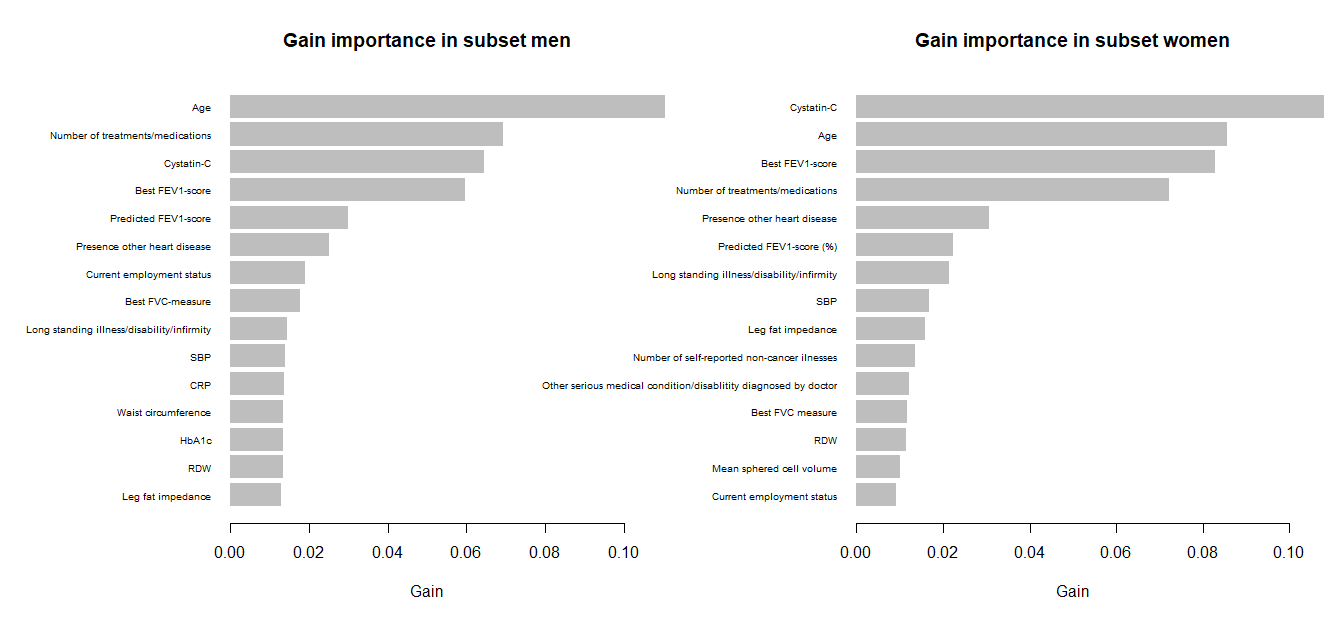
**

**Legend:** Gain importance of XGBoost models in the subset data, limited to the 15 most important predictors. Gain values are comparable between sexes. Age had a stronger effect on prediction accuracy in men than women, while this hold for cystatin-c in women.

**Figure S8: Correlation plots of high-risk variables as identified in men.**

**
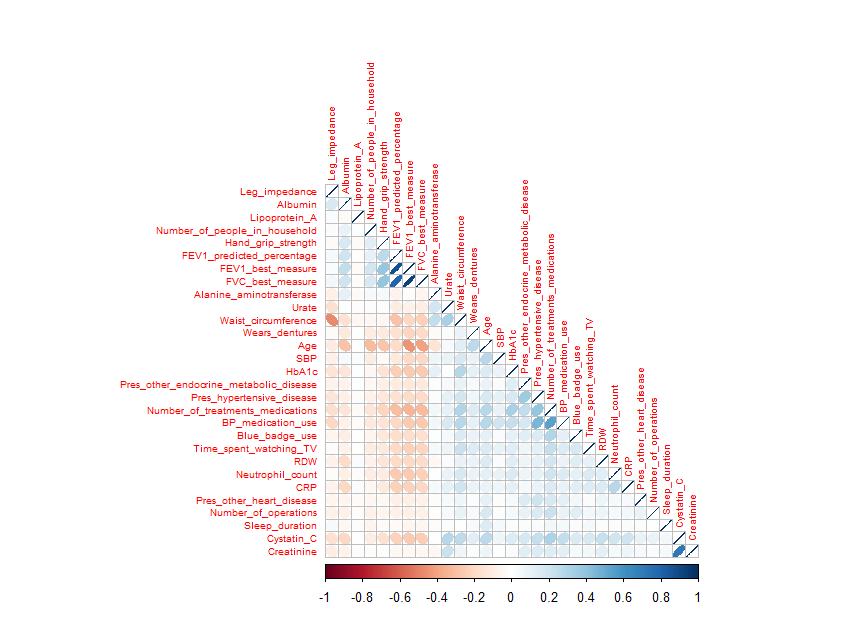
**

**Legend:** Correlation for continuous high-risk variables, identified in men for any of the prediction models.

**Figure S9: Correlation plots of high-risk variables as identified in women.**


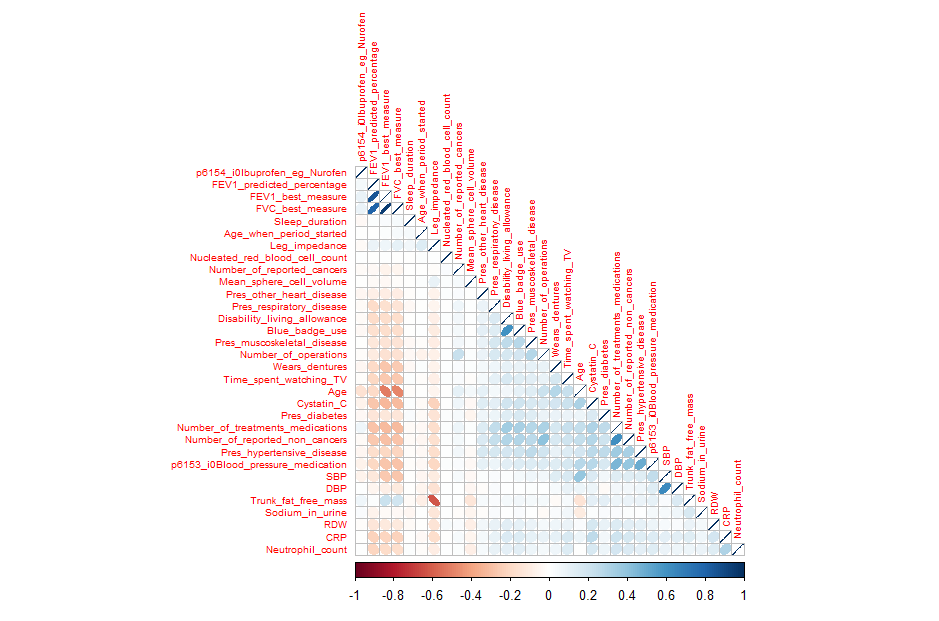


**Legend:** Correlation for continuous high-risk variables, identified in women for any of the prediction models.
